# Effect of feeding patterns on growth and nutritional status of children aged 0-24 months: A Chinese cohort study

**DOI:** 10.1371/journal.pone.0224968

**Published:** 2019-11-19

**Authors:** Qianling Tian, Xiao Gao, Tingting Sha, Cheng Chen, Ling Li, Qiong He, Gang Cheng, Xialing Wu, Fan Yang, Yan Yan

**Affiliations:** Department of Epidemiology and Health Statistics, Xiangya School of Public Health, Central South University, Changsha, Hunan, China; University 9 of July, BRAZIL

## Abstract

**Objective:**

This study was aimed to examine the effect of feeding patterns on growth and nutritional status of children aged 0~24 months.

**Methods:**

We conducted a cohort study with an initial sample of 927 children. Considering the follow-up losses, 903, 897, 895, 897, 883, 827 and 750 children were followed up at 1, 3, 6, 8, 12, 18 and 24 months, respectively. Children were grouped according to exclusive breastfeeding (EBF) duration in the first 6 months: (1) never EBF; (2) EBF ≤ 3 months: EBF ≤ 3 months and stopped BF after 3 months or EBF ≤ 3 months and BF = 6 months or EBF ≤ 3 months and BF after 3 months, had formula and/or solids; (3) EBF for 3 ~ 6 months: BF < 3 months and EBF for 3 ~ 6 months or EBF for 3 ~ 6 months and BF < 3 months, had formula and/or solids; (4) EBF = 6 months. We used Z-scores to evaluate the growth and nutritional status of children, used the generalized estimation equation to compare the difference between feeding patterns.

**Results:**

The generalized estimation equation results showed that Weight-for-age Z-score (WAZ), Length-for-age Z-score (LAZ), and Weight-for-length Z-score (WLZ) in different feeding patterns had statistical significance. The WAZ in EBF for 6 months group was higher in the first 8 months, in never EBF group was higher after 12 months old; the LAZ in EBF for 6 month group was lower than other groups; the WLZ in EBF for 6 months group was higher than EBF for 3 ~ 6 months group. The EBF ≤ 3 months group had higher underweight, stunting, and wasting rates. The EBF for 6 months had a higher stunting rate; the never EBF and EBF for 6 months groups had higher overweight and obesity rates.

**Conclusions:**

In conclusion, different feeding patterns affect growth and nutritional status in children, so proper guidelines should be implemented to improve nutritional status and promote the growth of children.

## Introduction

Human milk is a critical source of nutrients for the first 6 months of life and a significant component of nutritional requirements for the first 2 years of life[1,2]. According to the World Health Organization (WHO), EBF in the first 6 months of life prevents more than one million deaths[3]. The position of the Academy of Nutrition and Dietetics is that exclusive breastfeeding provides optimal nutrition and health protection for the first six months of life and that breastfeeding with complementary foods from 6 months until at least 12 months of age is the ideal feeding pattern for children[4]. Some researchers found that breastfeeding can significantly reduce the risk of obesity in children[5–9] and lipidomic profiling of breast-fed and formula-fed children has striking differences[10]. Compared to children who did not breastfeed, children who were exclusively breastfed until four-month-old, followed by mixed breastfeeding had better communication, social interaction, and cognition[11]. The WHO and the United Nations Children’s Fund recommended that children initiated breastfeeding within the first hour of birth[12] and were exclusively breastfed for the first 6 months of life, from the age of 6 months, children should begin eating safe and adequate complementary food while continuing to breastfeed for up to two years and beyond[13]. The early nutritional status of the children is related to their feeding pattern; a study demonstrated that the Infant and Child Feeding Index had a significant association with height, weight, height-for-age Z-score and weight-for-age Z-score[14].

In 2006, the WHO released a new growth standard for children, which was conducted in six countries from 1997 to 2003[15]. At first, China was considered as the study site in East Asiahad[16], for China performs growth surveys every ten years, the Chinese government decided not to participate in the Multicentre Growth Reference Study. Between May and October 2005, China conducted the fourth survey in 9 cities; the current growth references were established based on this survey[17]. For current growth references in China were from cross-section data, and that was nearly ten years ago, a study used data from six birth cohorts of China Birth Cohort Consortium to provide an update on how healthy children from birth to 24 months are growing in modern China[18].

In this article, first, we analyzed the data from a cohort study conducted at Changsha, China to acknowledge the growth status of children. Second, we compared the growth data and the nutritional status in different feeding patterns (divided by EBF duration at the first 6 months) to examine the effect of feeding patterns on children’s growth and nutrition status in Changsha, China.

## Materials and methods

### Design and participants

The data in this study were from a 24-months birth cohort study of Chinese mother-child pairs, the purpose of which was to explore child growth with a panel data model. This study was approved by the Independent Ethics Committee Institute of Clinical Pharmacology, Central South University, Changsha, China. We used some data such as length, weight, feeding pattern in the cohort study to explore the growth of children and the effect of different feeding patterns on the children’s growth and nutrition status. The Community Health Service Centers of Xinhelu, Dongfenglu, and Sifangping streets of Kaifu District in Changsha, China were selected as the investigation sites. The cluster sampling method was used to select the child who was born in these Community Health Service Centers during 2015. From Jan 1, 2015, to Dec 31, 2015, a total of 1,286 infants were born in the three streets. The inclusion criteria of this 24-month prospective cohort study were as follows: (1) mothers and their children who were living on the three streets above and have completed records at the any Community Health Service Centers. (2) agreed to engage in our study and sign the informed consent. (3) those who were singleton births. (4) mothers who had no mental illnesses or brain diseases. (5) Children who had no congenital diseases. Excluded the mother-child pairs who did not meet the inclusion criteria, finally, 976 eligible mother-child pairs were enrolled in the prospective cohort study. In this study, the exclusion criteria for the child were as follows: (1) gestational age < 37 weeks (n = 44); (2) gestational age > 42 weeks (n = 5). Due to this was a cohort study, some of the children were lost to follow-up, so the sample size was different for different months, the sample size at 0, 1, 3, 6, 8, 12, 18, and 24 month-old were 927, 903, 897, 895, 897, 883, 827, and 750 respectively.

### Feeding patterns

According to the EBF duration at the first 6 months, children were divided into 4 groups: (1) never EBF; (2) EBF ≤ 3 months: EBF ≤ 3 months and stopped BF after 3 months or EBF ≤ 3 months and BF = 6 months or EBF ≤ 3 months and BF after 3 months, had formula and/or solids; (3) EBF for 3 ~ 6 months: BF < 3 months and EBF for 3 ~ 6 months or EBF for 3 ~ 6 months and BF < 3 months, had formula and/or solids; (4) EBF = 6 months. We followed up the children at 1, 3, 6, 8, 12, 18, and 24 months of age. The feeding patterns of the children in different months were collected by asking their mothers/guardians what feeding method their children were in during this period and whether/when they had added formula milk or solids to them. According to the mothers’ answer in different months, we recorded the children’s feeding pattern in the questionnaire in different follow-up age. At the first 6 months, only 1, 3, 6 month-old feeding patterns were available, so we divided the children into never EBF, EBF ≤ 3 months, EBF for 3 ~ 6 months, and EBF for 6 months. EBF was defined as 0 ~ 6 month-old children shall not accept any other food, drink, and even water beside breast milk[13].

### Data collection

#### Physical measurement

Length (to the nearest 0.1 cm) was measured without shoes using the Pediatric Length Board, weight (to the nearest 0.1 kg) was measured without shoes and in light clothing using a portable electronic scale, both length and weight were measured at seven target points twice to increase the reliability. The data of weight and length were collected by the doctors of Community Health Service Centers at regular checkups during their 0 ~ 24 month-old. The birth weight and birth length were from the children's maternity handbook.

#### Calculation of Z-scores

We used the WHO Anthro software to calculate the Z-scores of weight-for-length (WLZ), weight-for-age (WAZ), length-for-age (LAZ) according to children’s sex, birth date, and checkup date. Based on the WHO growth standards, stunting was defined LAZ < -2, underweight was defined as WAZ < -2; wasting was defined as weight lighter than the corresponding weight of WLZ of -2 for particular length and sex; overweight means as weight heavier than the corresponding weight of WLZ of 1 for specific length and sex; obesity was defined as weight heavier than the corresponding weight of WLZ of 2 for particular length and sex.

#### Families and children factors

The factors of families and children were collected using a self-made questionnaire by face-to-face interviews with the mothers/guardians. The questionnaire was made by our team and had been discussed with experts several times. In the baseline survey, we collected the children’s sex, maternal and paternal age, maternal and paternal education level, family socioeconomic, gestational weeks, delivery mode, and initiation of breastfeeding. Children’s feeding patterns, feeding quantity, feeding times, time to add formula and solids, and other information were collected at seven target ages (1, 3, 6, 8, 12, 18, and 24 month-old).

### Statistical analyses

The data were checked manually for completeness and input via EpiData version 3.1 (EpiData Association, Odense, Denmark) by two investigators. All statistical analyses were performed using SPSS version 20 (IBM, New York, USA). Continuous variables were described using mean ± standard deviation (SD), while categorical variables were using percentage. One sample t-test, One-way ANOVA, generalized estimating equation, and chi-square test were used to compare the general characteristics, children’s growth status and nutritional status in different feeding groups. *P* values < 0.05 were considered significant.

## Results

### Characteristic of children and mothers by feeding patterns

As showed in **Table 1**, the birth weight in every feeding groups was 3.32 to 3.40 kg, and the birth length were all 50 cm. Most (above 80%) maternal and paternal education levels were university or college; the maternal age, sex of the child, family income per capita, and the mode of delivery can affect feeding patterns (*P <* 0.05) (**Table 1**).

**Table 1:** Basic data of children and their parents in different feeding groups, according to clinical and socioeconomic and demographic characteristics. Kaifu District in Changsha, China, 2015.

	1 (n = 117)	2 (n = 243)	3 (n = 433)	4 (n = 134)	*P*
Birth weight (Mean ± SD) (kg)	3.35 ± 0.41	3.32 ± 0.41	3.39 ± 0.40	3.40 ± 0.36	0.127
Birth length (Mean ± SD) (cm)	50.0 ± 0.6	50.0 ± 0.6	50.0 ± 0.7	50.0 ± 0.5	0.766
Sex n (%)					
Boy	58 (49.6) 59.3	138 (56.8)	206 (47.6)	79 (59.0)	
Girl	59 (50.4)	105 (43.2)	227 (52.4)	55 (41.0)	0.037
Maternal age (years) n (%)	30.4				
18 ~ 25	2 (1.7)	9 (3.7)	24 (5.5)	9 (6.7)	
25 ~ 30	45 (38.5)	101 (41.6)	223 (51.5)	62 (46.3)	
30 ~ 35	49 (41.9)	100 (41.2)	135 (31.2)	53 (39.6)	
≥ 35	21 (17.9)	33 (13.6)	51 (11.8)	10 (7.5)	0.010
Paternal age (years) n (%)	32.6			32.0	
18 ~ 25	4 (3.4)	4 (1.6)	12 (2.8)	3 (2.2)	
25 ~ 30	31 (26.5)	67 (27.6)	151 (34.9)	41 (30.6)	
30 ~ 35	42 (35.9)	104 (42.8)	164 (37.9)	54 (40.3)	
≥ 35	40 (34.2)	68 (28.0)	106 (24.5)	36 (26.9)	0.374
Maternal Education (years) n (%)					
≤ 9	6 (5.1) 6	12 (4.9)	15 (3.5)	5 (3.7)	
10 ~ 12	16 (13.7) 12.1	30 (12.3) 11.8	52 (12.0)	15 (14.2) 14.2	
> 12%	95 (81.2) 81.9	201 (82.7) 84.9	366 (84.5)	110 (82.1) 82.1	0.931
Paternal education (years) n (%)					
≤ 9	4 (3.4) 6.5	14 (5.8)	10 (2.3)	8 (6.0)	
10 ~ 12	16 (13.7) 12.6	32 (13.2) 12.9	49 (11.3)	14 (10.4) 10.4	
12	97 (82.9) 80.9	197 (81.1) 85.5	374 (86.4)	112 (83.6) 83.6	0.231
Mode of delivery n (%)					
Vaginal delivery	53 (45.3)	138 (56.8)	288 (66.5)	84 (62.7)	
C-section	64 (54.7)	105 (43.2)	145 (33.5)	50 (37.3)	0.000
Family income per capita (yuan/month) n (%)					
< 2000	10 (8.5) 2	6 (2.5)	11 (2.5)	7 (5.2)	
2001 ~ 5000	68 (58.1) 53.8	131 (53.9) 55.4	224 (51.7)	65 (48.5)	
5001 ~ 10000	33 (28.2) ) 40.7	96 (39.5) 36.0	174 (40.2)	60 (44.8) 44.8	
> 10000	6 (5.1) 3.5	10 (4.1)	24 (5.5)	2 (1.5)	0.013

1 represent never had EBF; 2 represent EBF ≤ 3 months: EBF ≤ 3 months and stopped BF after 3 months or EBF ≤ 3 months and BF = 6 months or EBF ≤ 3 months and BF after 3 months, had formula and/or solids; 3 represent EBF for 3 ~ 6 months: BF < 3 months and EBF for 3 ~ 6 months or EBF for 3 ~ 6 months and BF < 3 months, had formula and/or solids; 4 represent EBF = 6 months.

### Growth of children

**Table 2** showed the weight, length, and BMI from birth to 24 months in our study, China recent research results, China growth references, and WHO growth standards.

**Table 2:** The weight, length, and BMI of children in our study, China recent research results, Chinese growth references, and WHO growth standards, by sex and age. Kaifu District, Changsha, Hunan, China, 2015.

Age (month)	boy												girl											
	weight				length				BMI				weight				length				BMI			
	1	2	3	4	1	2	3	4	1	2	3	4	1	2	3	4	1	2	3	4	1	2	3	4
0	3.42	3.39	3.32*	3.3^#^	50.0	50.3^†^	50.4*	49.9^#^	13.7	13.4^†^	13.07*	13.4^#^	3.32	3.30	3.21*	3.2^#^	50.0	49.9^†^	49.7^#^	491^#^	13.3	13.3	13.00*	13.3
1	4.66	4.70^†^	4.51*	4.5^#^	55.2	54.9^†^	54.8*	54.7^#^	15.3	15.6^†^	14.91*	14.9^#^	4.45	4.51^†^	4.20*	4.2^#^	54.3	54.2	53.7^#^	53.7^#^	15.0	15.4^†^	14.52*	14.6^#^
3	6.86	6.87	6.70*	6.4^#^	62.4	62.2	62.0*	61.4^#^	17.6	17.7	17.48*	16.9^#^	6.45	6.46	6.13*	5.8^#^	61.2	61.0^†^	60.6^#^	59.8^#^	17.2	17.2	16.69*	16.4^#^
6	8.39	8.61^†^	8.41	7.9^#^	68.4	68.8^†^	68.4	67.6^#^	18.0	18.2^†^	17.96	17.3^#^	7.93	8.06^†^	7.77*	7.3^#^	66.9	67.5^†^	66.8	65.7^#^	17.7	17.7	17.41*	16.9^#^
8	9.16	9.34^†^	9.05*	8.6^#^	71.5	71.9^†^	71.2*	70.6^#^	18.0	18.1^†^	17.79*	17.3^#^	8.61	8.81^†^	8.41*	7.9^#^	70.1	70.6^†^	69.6^#^	68.7^#^	17.6	17.6	17.31*	16.8^#^
12	10.01	10.29^†^	10.05	9.6^#^	76.1	76.6^†^	76.5*	75.7^#^	17.3	17.6^†^	17.19	16.8^#^	9.53	9.74^†^	9.40*	8.9^#^	74.9	75.4^†^	75.0	74.0^#^	17.0	17.2^†^	16.74*	16.4^#^
18	11.27	11.62^†^	11.29	10.9^#^	82.6	83.1^†^	82.7	82.3^#^	16.6	16.8^†^	16.47	16.1^#^	10.73	11.03^†^	10.65	10.2^#^	81.4	81.8^†^	81.5	80.7^#^	16.2	16.4^†^	16.03*	15.7^#^
24	12.51	12.85^†^	12.54	12.2^#^	88.3	89.0^†^	88.5	87.8^#^	16.1	16.2^†^	16.07	15.7^#^	11.98	12.28^†^	11.92	11.5^#^	87.2	87.8^†^	87.2	86.4^#^	15.7	16.0^†^	15.67	15.4^#^

The number 1, 2, 3, and 4 represent our study, China recent research results (2015 data), China growth references (2005 data), and WHO growth standards, respectively. The reference group was our study

^†^ *p* < 0.05

* *p* < 0.05

^#^ *p <* 0.05.

The weight of children in our study was heavier than the WHO growth standard (*P <* 0.05), but lighter (*P <* 0.05) than Chinese recent research result except for 0 and 3 months old. Compared to China growth reference, the difference of weight had statistical significance in boys 0 ~ 8 month-old (except 6 month-old), and girls 0~12 month-old, weight in our study was heavier than China growth reference, after 12 months old, the difference had no statistical significance.

Compared to the 2015 research result, in our study, the length in boys was shorter at birth, in girls was higher at birth (*P <* 0.05). After 6 month-old, the length of children in the 2015 six birth cohorts study was higher than in our study (*P <* 0.05). Compared to 2005 China nine cities research result, length in our study was higher before 8 month-old (except 6 month-old). In other months, length in two studies had no statistical difference. In the Comparison with the WHO growth standard, from birth to 24 months, children in our study were higher.

The BMI of 2015 research were higher than our study in 0, 1, 6, 8, 18, and 24 month-old in boys and 1, 12, 18, and 24 month-old in girls (*P <* 0.05). Compared to the WHO growth standard, BMI in our study was higher (*P <* 0.05) (**Table 2**).

### The relationship between feeding and growth

The children were divided into 4 groups based on feeding patterns of the first 6 months. At the first 3 months, the weight in group 3 (EBF for 3 ~ 6 months: BF < 3 months and EBF for 3 ~ 6 months or EBF for 3 ~ 6 months and BF < 3 months, had formula and/or solids) was heavier than group 1 (never EBF) and group 2 (EBF ≤ 3 months: EBF ≤ 3 months and stopped BF after 3 months or EBF ≤ 3 months and BF = 6 months or EBF ≤ 3 months and BF after 3 months, had formula and/or solids), after 8 months, it was lighter than group 1 and group 2. The weight in group 4 (EBF = 6 months) was heavier than group 3 but lighter than group 1 and group 2 when age was over 12 months. Used the generalized estimation equation to compare the difference of weight among 4 groups, after controlling the confounders (child sex, age, maternal age, maternal education, paternal age, paternal education, mode of delivery, family income per capita, birth weight, and birth length), the results showed the weight in group 1 was heavier than group 3 (*P <* 0.05) (**Table 3**).

**Table 3:** The weight, length, and BMI of children in different feeding groups and different months. Kaifu District, Changsha, China, 2015.

Age (month)	Weight (Mean ± SD)				Length (Mean ± SD)				BMI (Mean ± SD)			
	1	2	3	4	1	2	3	4	1	2	3	4
0	3.35 ± 0.41	3.32 ± 0.41	3.39 ± 0.40	3.40 ± 0.40	50.0 ± 0.6	50.0 ± 0.6	50.0 ± 0.7	50.0 ± 0.5	13.4 ± 1.5	13.3 ± 1.6	13.6 ± 1.5	13.6 ± 1.4
1	4.49 ± 0.48	4.51 ± 0.53	4.60 ± 0.51	4.56 ± 0.46	54.5 ± 1.7	54.5 ± 1.8	55.0 ± 1.8	54.6 ± 1.7	15.1 ± 1.3	15.2 ± 1.5	15.2 ± 1.3	15.3 ± 1.2
3	6.58 ± 0.68	6.59 ± 0.74	6.69 ± 0.74	6.79 ± 0.75	61.5 ± 2.0	61.7 ± 2.1	61. 9 ± 2.1	61.9 ± 2.1	17.4 ± 1.2	17.3 ± 1.6	17.4 ± 1.6	17.7 ± 1.5
6	8.15 ± 0.87	8.17 ± 0.85	8.15 ± 0.88	8.26 ± 0.89	67.5 ± 2.0	67.7 ± 2.2	67.7 ± 2.2	67.7 ± 2.4	17.9 ± 1.5	17.8 ± 1.5	17.8 ± 1.4	18.1 ± 1.6
8	8.92 ± 0.85	8.89 ± 0.96	8.85 ± 0.96	9.03 ± 0.94	70.9 ± 2.3	70.9 ± 2.3	70.7 ± 2.4	70.7 ± 2.4	17.8 ± 1.3	17.7 ± 1.5	17.7 ± 1.4	18.1 ± 1.6
12	9.99 ± 0.93	9.83 ± 1.02	9.66 ± 1.02	9.88 ± 1.09	75.9 ± 2.4	75.6 ± 2.5	75.4 ± 2.6	75.4 ± 2.2	17.4 ± 1.3	17.2 ± 1.4	17.0 ± 1.5	17.3 ± 1.4
18	11.06 ± 1.07	11.15 ± 1.24	10.92 ± 1.14	11.02 ± 1.08	82.1 ± 2.7	82.1 ± 3.0	82.1 ± 2.8	81.7 ± 2.7	16.5 ± 1.4	16.5 ± 1.4	16.2 ± 1.3	16.6 ± 1.4
24	12.48 ± 1.11	12.32 ± 1.35	12.18 ± 1.25	12.20 ± 1.22	87.8 ± 2.7	88.0 ± 3.1	87.7 ± 3.1	87.5 ± 2.9	16.3 ± 1.3	15.9 ± 1.2	15.8 ± 1.1	16.0 ± 1.5
*P*	&					@	@					&

1 represent never had EBF; 2 represent EBF ≤ 3 months: EBF ≤ 3 months and stopped BF after 3 months or EBF ≤ 3 months and BF = 6 months or EBF ≤ 3 months and BF after 3 months, had formula and/or solids; 3 represent EBF for 3 ~ 6 months: BF < 3 months and EBF for 3 ~ 6 months or EBF for 3 ~ 6 months and BF < 3 months, had formula and/or solids; 4 represent EBF = 6 months.

& Compared with group 3, it was higher, *P*<0.05

@ Compared with group 4, it was higher, *P*<0.05

Used the generalized estimation equation to compare the difference of length among 4 groups, after controlling the confounders (child sex, age, maternal age, maternal education, paternal age, paternal education, mode of delivery, family income per capita, birth weight, and birth length), the results showed the length in group 2 and group 3 were higher than group 4 (*P <* 0.05) (**Table 3**).

At the first 8 months, the BMI in group 4 was higher than other groups; after 12 months, the BMI in group 3 was lower than group 1 and group 4. Used the generalized estimation equation to compare the difference of BMI among 4 groups, after controlling the confounders (child sex, age, maternal age, maternal education, paternal age, paternal education, mode of delivery, family income per capita, birth weight, and birth length), the results showed the BMI in group 4 was higher than group 3 (*P <* 0.05) (**Table 3**).

#### Z-scores

**Table 4** presented the WAZ, LAZ, and WLZ in different feeding patterns and different months. At the first 8 months, WAZ in EBF for more than 3 months groups were higher than EBF for less than 3 months groups in total, after 12 month-old, EBF for less than 3 months groups were higher than EBF for more than 3 months groups. The results of the generalized estimation equation showed the feeding patterns had a significant difference in the effect of WAZ on children (*P <* 0.05), WAZ in group 1 and group 2 were higher than in group 3 (**Table 4**).

**Table 4:** The WAZ, LAZ, and WLZ in different feeding groups and different months. Kaifu District, Changsha, China, 2015.

Age (Month)	WAZ (Mean ± SD)				LAZ (Mean ± SD)				WLZ (Mean ± SD)			
	1	2	3	4	1	2	3	4	1	2	3	4
0	0.09 ± 0.84	0.03 ± 0.86	0.19 ± 0.83	0.18 ± 0.75	0.28 ± 0.37	0.22 ± 0.37	0.25 ± 0.40	0.20 ± 0.30	-0.01± 1.16	-0.13 ± 1.15	0.10 ± 1.13	0.16 ±1.07
1	0.02 ± 0.78	-0.04 ± 0.83	0.13 ± 0.84	0.16 ± 0.79	-0.05 ± 0.84	-0.14 ± 0.92	0.14 ± 0.91	-0.03 ± 0.90	0.11 ± 0.94	0.12 ± 1.09	0.02 ± 1.01	0.23 ± 0.97
3	0.37 ± 0.85	0.37 ± 0.89	0.53 ± 0.89	0.56 ± 0.90	0.16 ± 1.01	0.21 ± 0.99	0.33 ± 0.99	0.24 ±0.98	0.41 ± 0.79	0.34 ± 1.01	0.43 ± 0.95	0.51 ± 0.96
6	0.46 ± 0.84	0.45 ± 0.91	0.46 ± 0.91	0.54 ± 0.92	0.26 ± 0.90	0.28 ± 0.94	0.29 ± 0.94	0.25 ± 0.95	0.51 ± 0.89	0.50 ± 0.95	0.48 ± 0.92	0.61 ± 0.96
8	0.51 ± 0.81	0.48 ± 0.91	0.50 ± 0.90	0.59 ± 0.90	0.41 ± 0.93	0.33 ± 0.98	0.33 ± 0.95	0.23 ± 0.97	0.54 ± 0.91	0.48 ± 0.95	0.50 ± 0.89	0.70 ± 0.95
12	0.53 ± 0.81	0.37 ± 0.86	0.24 ± 0.87	0.33 ± 0.86	0.30 ± 0.95	0.15 ± 0.99	0.13 ± 0.98	-0.02 ± 0.93	0.53 ± 0.87	0.41 ± 0.91	0.25 ± 0.93	0.44 ± 0.87
18	0.33 ± 0.82	0.33 ± 0.94	0.22 ± 0.86	0.26 ± 0.90	0.16 ± 0.93	0.12 ± 1.07	0.19 ± 0.98	-0.08 ± 0.95	0.37 ± 0.89	0.36 ± 0.98	0.19 ± 0.88	0.38 ± 0.98
24	0.41 ± 0.72	0.22 ± 0.90	0.17 ± 0.85	0.14 ± 0.80	0.19 ± 0.82	0.16 ± 0.99	0.17 ± 0.99	-0.02 ± 0.96	0.37 ± 0.90	0.11 ± 0.91	0.07 ± 0.87	0.16 ± 0.93
*P*	&	&			@	@	@		&	&		&

1 represent never had EBF; 2 represent EBF ≤ 3 months: EBF ≤ 3 months and stopped BF after 3 months or EBF ≤ 3 months and BF = 6 months or EBF ≤ 3 months and BF after 3 months, had formula and/or solids; 3 represent EBF for 3 ~ 6 months: BF < 3 months and EBF for 3 ~ 6 months or EBF for 3 ~ 6 months and BF < 3 months, had formula and/or solids; 4 represent EBF = 6 months.

& Compared with group 3, it was higher, *P <* 0.05

@ Compared with group 4, it was higher, *P <* 0.05

The generalized estimation equation results showed the LAZ in different feeding groups had statistical difference too (*P <* 0.05), LAZ in group 1, group 2, and group 3 were higher than group 4. The LAZ in group 4 was lower than 0 when age was over 12 months old (**Table 4**). At the first 8 months, the WLZ increased with time, after 8 months, it decreased with time. The generalized estimation equation results showed the WLZ had a difference in different feeding groups, group 1, group 2, and group 4 were higher than group 3 (*P <* 0.05) (Table 4).

### Nutritional status of the children

**Fig 1:**
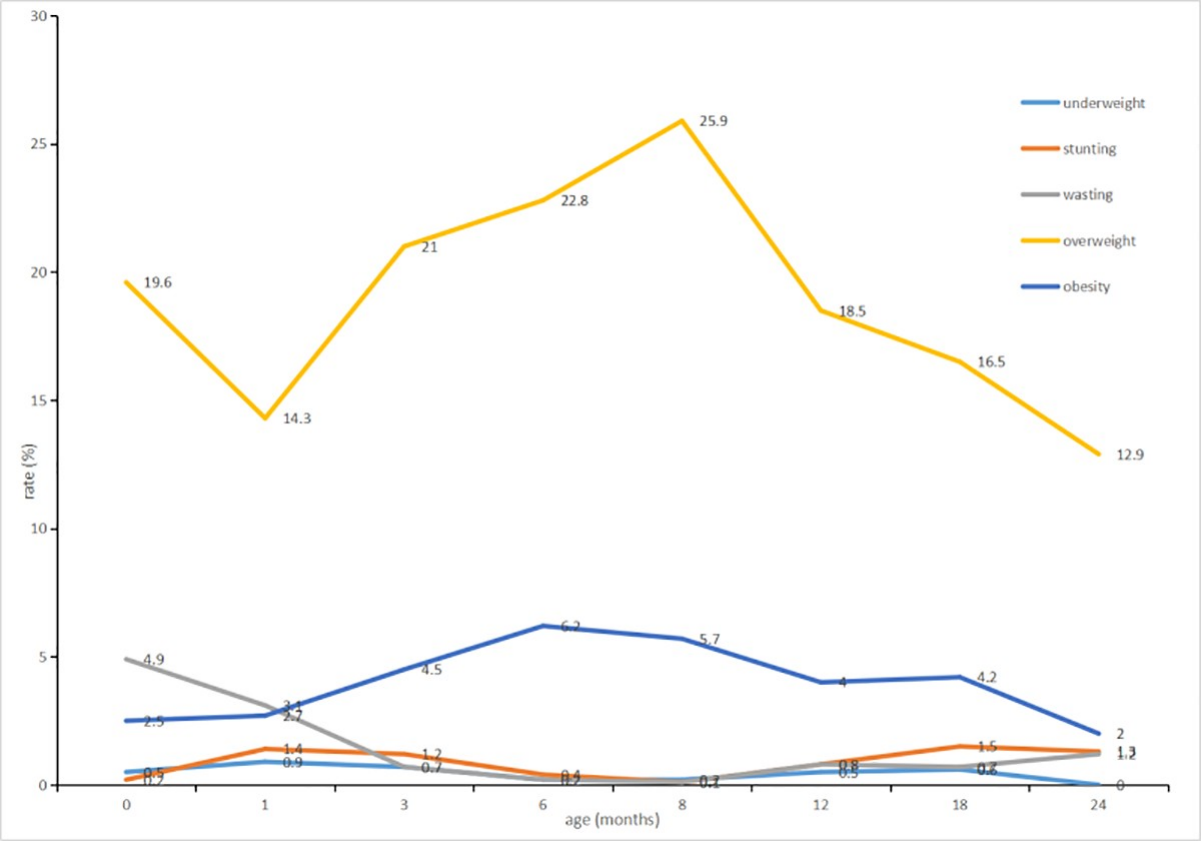
The nutritional status of children from 0 to 24 months. Kaifu District, Changsha, Hunan, China, 2015.

The rate of overweight and obesity in our study increased in the first 8 months, then decreased with time. The highest overweight rate was 25.9% at 8 months old, and the highest obesity rate was 6.2% at 6 months old in our study. The underweight rate was low and did not change significantly over time. The rate of stunting and wasting had decreased in the first 8 months but had shown a rising trend in age after 8 month-old (**Fig 1**).

The underweight, stunting, and wasting rates in our study were not very high. The overweight rate in group 1 was higher than in other groups when age was over 8 months old. At the first 8 months, the obesity rate in group 4 was higher than in other groups, but after 12 months, the obesity rate in group 1 was at a higher level. The stunting rate in group 2 and 4 had an increased tendency when age was over 12 months. The rates of underweight, stunting, and wasting in group 2 were high than in group 1 and group 3. The generalized estimation equation results showed that different feeding patterns had statistical significance on children's nutritional status. The underweight rate in group 2 was higher than in group 1. The overweight rate in group 1, 2, and 4 were higher than group 3 (*P <* 0.05), and the obesity rate in group 4 was higher than group 3 (*P <* 0.05) (**Table 5**).

**Table 5:** The rates of nutritional status in different feeding patterns and months. Kaifu District, Changsha, Hunan, China, 2015.

Age (Month)	Underweight % (n)				Stunting % (n)				Wasting % (n)				Overweight % (n)				Obesity % (n)			
	1	2	3	4	1	2	3	4	1	2	3	4	1	2	3	4	1	2	3	4
0	0.9(1)	0.8(2)	0.2(1)	0.7(1)	0.0(0)	0.0(0)	0.5(2)	0.0(0)	6.1(7)	6.8(16)	4.3(18)	2.3(3)	22.8(26)	17.7(42)	19.1(81)	21.8(29)	1.8(2)	1.7(4)	3.5(15)	1.5(2)
1	0.0(0)	1.3(3)	0.9(4)	0.8(1)	0.9(1)	1.3(3)	1.9(8)	0.8(1)	2.6(3)	4.3(10)	3.1(13)	1.5(2)	10.4(12)	17.9(42)	12.7(54)	16.2(21)	3.4(4)	2.1(5)	2.4(10)	3.8(5)
3	0.9(1)	1.3(3)	0.5(2)	0.0(0)	1.7(2)	13(3)	1.0(4)	1.6(2)	0.0(0)	1.3(3)	0.5(2)	0.8(1)	23.1(27)	19.7(45)	21.4(89)	20.3(26)	0.9(1)	3.9(9)	5.3(22)	6.3(8)
6	0.0(0)	0.4(1)	0.2(1)	0.0(0)	0.9(1)	0.9(2)	0.2(1)	0.0(0)	0.9(1)	0.4(1)	0.0(0)	0.0(0)	22.8(26)	21.7(50)	22.0(91)	27.5(36)	5.3(6)	7.4(17)	5.3(22)	7.6(10)
8	0.0(0)	0.4(1)	0.0(0)	0.8(1)	0.0(0)	0.0(0)	0.2(1)	0.0(0)	0.0(0)	0.4(1)	0.0(0)	0.0(0)	28.7(33)	25.9(60)	24.4(100)	28.0(37)	4.3(5)	5.6(13)	4.9(25)	9.8(13)
12	0.0(0)	0.9(2)	0.5(2)	0.0(0)	0.0(0)	1.3(3)	0.5(2)	1.5(2)	0.0(0)	0.4(1)	1.5(6)	0.0(0)	22.9(25)	22.4(51)	14.3(59)	21.4(28)	5.5(6)	3.9(9)	3.6(15)	3.8(5)
18	0.0(0)	0.9(2)	0.5(2)	0.8(1)	1.0(1)	1.9(4)	1.3(5)	1.6(2)	1.0(1)	0.9(2)	0.5(2)	0.8(1)	23.8(24)	18.8(40)	12.9(49)	17.7(23)	4.0(4)	3.8(8)	3.7(14)	6.9(9)
24	0.0(0)	0.0(0)	0.0(0)	0.0(0)	0.0(0)	2.1(4)	0.9(3)	2.6(3)	2.2(2)	1.0(2)	1.2(4)	0.9(1)	15.6(14)	13.5(26)	11.0(38)	15.5(18)	4.4(4)	1.6(3)	1.4(5)	2.6(3)
*P*		%											&	&		&				&

1 represent never had EBF; 2 represent EBF ≤ 3 months: EBF ≤ 3 months and stopped BF after 3 months or EBF ≤ 3 months and BF = 6 months or EBF ≤ 3 months and BF after 3 months, had formula and/or solids; 3 represent EBF for 3 ~ 6 months: BF < 3 months and EBF for 3 ~ 6 months or EBF for 3 ~ 6 months and BF < 3 months, had formula and/or solids; 4 represent EBF = 6 months.

% Compared with group 1, it was higher, *P* < 0.05

& Compared with group 3, it was higher, *P* < 0.05

## Discussion

This study was a cohort study conducted in a district of Changsha, Hunan, China. The results represent the growth status of children from 0 to 24 months in this district. The collection of feeding patterns is essential to this study, so at the follow-up stage, we asked the children’s mothers/guardians about the feeding information as detailed as possible to get more credible results.

Overall, compared to China recent research, the weight in our study was lighter than it, the length in our study was shorter than it, and the average BMI in our study was lower than it. Compared to China growth reference, the weight in our study was heavier than it, the average length was higher in the first 8 months, and the average BMI was bigger than it. Compared to the WHO growth standard, the weight, length, and BMI in this study were all higher, which also found in other studies[17–19].

In the comparison of children growth data based on feeding patterns, we found, children in EBF for less 3 months groups were lighter than EBF more than 3 months groups at first 3 months, however, after 12 months, EBF less than 3 months groups were heavier. The EBF may have a near-term impact on children’s weight, but in a long time, the shorter the EBF duration, the heavier the child. In terms of EBF for more than 3 months, EBF = 6 months was heavier than EBF for 3 ~ 6 months. The difference in length was that EBF = 6 months group was shorter than other groups from 12 months, children who added formula might grow faster than children who have not added, this is the same as the results of Brazilian infants[20].

The generalized estimation equation results showed that different feeding patterns affected the weight, length, WAZ, LAZ, and WLZ. From 0 to 24 months old, the WAZ in never EBF and EBF ≤ 3 months groups were higher than EBF for 3 ~ 6 months group, the LAZ in never EBF, EBF ≤ 3 months, and EBF for 3 ~ 6 months groups were higher than EBF = 6 months group, and the WLZ in never EBF, EBF ≤ 3 months, and EBF = 6 months groups were higher than EBF for 3 ~ 6 months group. Compared to children who are other feeding patterns, after 12 months old, EBF for 6 months group have a higher stunting rate, that may be caused by lower dietary diversity index[21]. Haschke F et al.[22] had found children who are exclusively breastfed 4–6 months or receive low protein follow-up formulas (high-quality protein) grow slower during the first 2–3 years than children fed high-protein formulas, this is consistent with our result.

The rate of overweight in never EBF, EBF ≤ 3 months, and EBF for 6 months groups were higher than EBF for 3 ~ 6 months, that means there was no linear relationship between the duration of EBF and overweight. Long or short EBF duration can increase the risk of children becoming overweight. Children who were EBF for 6 months have a higher overweight rate at the first 8 months, but children who were EBF for less than 3 months have a higher overweight rate at the age of 8 to 24 months. The rate of obesity was higher at EBF for 6 months group from 0 to 24 months old, but at 24 months, the never EBF group have a higher obesity rate. In the short term, EBF can increase the overweight and obesity rates of child, in the long term, never EBF child is more likely to be overweight and obesity than exclusively breastfed children[23]. At 24 months, the highest overweight rate is 15.6%, and the highest obesity rate is 4.4%, which were higher than that in Chongqing urban area several years ago[24] and the 2005 China national nutrition and health survey data[25]. The prevalence of overweight and obesity is becoming a serious public health problem for our country. In our study, we used the WHO standard to assess the nutritional status of children, which may overestimate the rate of overweight and obesity, for the weight and length of children in China are heavier and higher than WHO growth standards[19]. Children who undernourished in wasting and underweight have declined over the years, and the nutritional deprivation of children has been alleviated in China over time[26]; this is a trend in China so that the rate of malnutrition in our study is low.

According to the results of our study, the effect of EBF should be reexamined. EBF for 6 months is not the best feeding pattern for all children. The optimal EBF duration will be different based on the individual characteristics of the neonates and mothers, as well as social, economic, and geographical factors[27,28]. The research results in our study provide a new understanding of EBF. The results indicate that EBF for 6 months can increase the obesity rate in early childhood, and the stunting rate was also higher after 12 months old. According to the generalized estimation equation results, the never EBF group had higher overweight and obesity rates in later childhood, while the EBF for 6 months group had higher overweight and obesity rates at the earlier childhood; the EBF ≤ 3 months group had the higher underweight, stunting, and wasting rates. The stunting rate in EBF for 6 months group was higher after 12 months, and the LAZ was lower than 0 after 12 months.

To sum up, EBF for 6 months is not the best feeding patterns, EBF for 3 ~ 6 months may more conducive to the overall growth of children. The effect of feeding patterns on children’s growth and nutritional status is not static; we should discuss it based on age. Given these differences found in this study, there still need studies to focus on children’s feeding patterns. The related organization should review recommendations on feeding. With the development of the social economy, the malnutrition rate has dropped, but overweight and obesity rates are rising. It is more and more critical to instruct parents on how to feed their babies properly. The findings of this study provide a reference for future research on child feeding.

The limitations of this study are as follows. First, the sample size is not very large, so need a study with a larger sample size to support. Second, due to this study was a cohort study, some samples were lost to follow-up randomly, so we do not know their growth status. Third, we used WHO standards to evaluate the nutritional status of children, which may be not appropriate for Chinese children, some studies found the WHO growth standard did not make suitable for any region and age[19,29,30].

## Conclusion

To conclude, our study suggests that different feeding patterns affect the growth and nutritional status in children. Firstly, the WAZ, LAZ, and WLZ in different feeding groups have a significant difference; never EBF has a greater effect on weight than other feeding patterns after 12 months. EBF for 6 months have less effect on length than other three groups. Secondly, EBF less than 3 months and EBF for 6 months may increase the rate of overweight and obesity, EBF ≤ 3 months can increase the rates of underweight, stunting, and wasting. To improve the growth and nutritional status of children, instructions for parents on how to feed their babies properly become more and more impendency. Parents should select an appropriate pattern to feed their children.

## Supporting information

S1 Text Data underlying the findings reported in this article (in English). (XLS)

S2 Text The questionnaire used in our study. (ZIP)
