# Supplementary material for: Effect of feeding patterns on growth and nutritional status of children aged 0-24 months: A Chinese cohort study
Source: PLoS One. 2019 Nov 19;14(11):e0224968. doi: 10.1371/journal.pone.0224968 (PMC6863544; doi:10.1371/journal.pone.0224968)
Supplement: S2 Text — (ZIP) [file pone.0224968.s002.zip › 1 month-old.docx]

**2015 Kaifu District Community Maternal and child health information collection（1 month-old）**

**number： □□□□□□**

Kaifu District Community Health Service center

Residential Address： District(country) Street(road) Community

Mother’s name： Telephone number：

Father’s name： Telephone number：

Name of child： Gender of child：

Date of birth of the child：

Information collection time： year month day Information collector：

Contents

[Inquiry Section 3](#_Toc416447862)

[Tab B:Pregnancy conditions 3](#_Toc416447863)

[Tab D:History of exposure to maternal risk factors 4](#_Toc416447864)

[Tab E :Maternal diet and nutrition during pregnancy 4](#_Toc416447865)

[Tab F:Postpartum survey form 6](#_Toc416447866)

[Tab G:Edingburgh postpartum depression scale 7](#_Toc416447867)

Tab [J:Follow-up record form for children aged 1 7](#_Toc416447868)

[Tab K:Illness of children 8](#_Toc416447869)

[Excerpt(self-filling)section 9](#_Toc416447870)

[Tab L:Living environment of pregnant families during pregnancy (Note：★indicates the part that needs to be asked) 9](#_Toc416447871)

[Tab M:Record of birth inspection during pregnancy 10](#_Toc416447872)

[Tab A:Basic information 11](#_Toc416447873)

[Tab B:Pregnancy conditions 13](#_Toc416447874)

[Tab C:Family history 13](#_Toc416447875)

[Tab H:Neonatal situation questionnaire 14](#_Toc416447876)

# Inquiries Section

# Tab B：Pregnancy conditions

| **B01** | Current number of family residents | | |
| --- | --- | --- | --- |
| **B02** | Household per capita income(yuan/month):⑴RMB 2000 and below ⑵RMB 2001-RMB 5000 ⑶RMB5001-RMB10000 ⑷RMB10001-RMB15000 ⑸RMB15000 and above | | |
| **B03** | Your weight before giving birth： Kg | | |
| **B04** | Have you ever had an abortion?（1）No**（skip to B06）** （2）Yes, please specify the number of times ___ | | |
| **B05** | In the event of an abortion，reason is： ⑴Planned parenthood ⑵Subjective reasons ⑶Fetal abnormalities ⑷Maternal diseases ⑸else | | |
| **B06** | Are you prepared for this pregnancy： ⑴No ⑵Yes | | |
| **B07** | Have you been screened for cervical cancer before this pregnancy？⑴No ⑵Yes, please specify | | |
| **B08** | Is there vomiting during your pregnancy？： ⑴No ⑵Yes，duration： day |  |  |
| **B09** | Is there vaginal bleeding during your pregnancy?： ⑴No **(skip to B11)**  ⑵Yes，duration： day |  |  |
| **B10** | During pregnancy, when you have vaginal bleeding?**（can be more selected）：** ⑴Early pregnancy ⑵Mid-pregnancy ⑶Late pregnancy |  |  |
| **B11** | Which of the following pregnancy complications do you suffer during pregnancy?**（can be more selected）：**  （1）No ⑵Anemia ⑶Diabetes ⑷Essential hypertension ⑸Hepatitis B ⑹Congenital heart disease ⑺Rheumatic heart disease ⑻Hypertensive heart disease ⑼Chronic nephritis ⑽Acute pyelonephritis ⑾Hyperthyroidism ⑿Other nephritis ⒀Else，please specify (99)Unknown | | |
| **B12** | Which of the following pregnancy complications do you suffer during pregnancy?**（can be more selected）：**  （1）No（2）Multiple pregnancies（3）Pregnancy hypertension disease（4）Anterior placenta（5）Amniotic fluid abnormalities（6）Fetal growth restriction（7）Fetal abnormalities（8）Gestational diabetes（9）Postponed pregnancy（10）Expired pregnancy（11）Threatened Abortion（12）Late threatened abortion (13) Stillbirth（14）Fetal macrosomia（15）Moderate anemia（16）ABO hemolysis（17）Pregnancy liver losing （18）Else，please specify （99）Unknown | | |

# Tab F：Postpartum survey form

| **F01** | Whether the birth was suffering from complications**（can be more selected）**： ⑴No ⑵Postpartum hemorrhage ⑶Amniotic fluid embolism ⑷Hysterorrhexis ⑸Abnormal umbilical cord ⑹Else | |
| --- | --- | --- |
| **F02** | Whether the perineum is complete after delivery of pregnant women： ⑴Completion ⑵incision ⑶Laceration | |
| **F03** | Is there no hysterectomy during childbirth?：⑴No ⑵Yes（please indicate the reason） | |
| **F04** | Your current weight： **Kg** | |
| **F05** | Does the child father smoke now?： ⑴No smoking ⑵1-4 cigarettes a day ⑶5-9 cigarettes a day⑷10-15 cigarettes a day ⑸15-20 cigarettes a day（6）More than 20 per day | |
| **F06** | Does the child father drink now? ⑴No ⑵Yes | |
| **F07** | Currently, your average number of days of passive smoking per week（more than 15 minutes a day）： ⑴0 day ⑵1-2 days ⑶3-5 days ⑷6-7days | |
| **F08** | Currently, the average number of days a child is passive smoking per week（more than 15 minutes a day）：⑴0day ⑵1-2 days ⑶3-5days ⑷6-7days | |
| **F09** | The first time a baby sucks a nipple after birth：⑴Within 30 minutes of delivery ⑵30-59 minutes ⑶1-2 hours ⑷3-24 hours ⑸＞24 hours， |  |
| **F10** | Whether the baby used a bottle before sucking the nipple： ⑴No ⑵Yes |  |
| **F11** | How long does the child begin to breastfeed after birth⑴within 30 minutes of delivery ⑵0.5-2 hours ⑶2-24 hours ⑷24-48 hours ⑸＞48 hours， |  |

# Tab J：Follow-up record form for children 1 month-old

| **J01** | Check date： year month day |
| --- | --- |
| **J02** | Real term age： month-old day |
| **J03** | Feeding style：（1）Exclusive breastfeeding（2）Mixed feeding （3）Formula feeding **(skip to J07)** |
| **J04** | Number of breast milk： （ times/day） |
| **J05** | Is the baby now out of breast milk？（1）No **(skip to J07)** （2）Yes，the month age of breast milk disconnection is month-old day |
| **J06** | What the reason why you weaned your child? **（can be more selected）**⑴Job requirements ⑵Fall ill ⑶No breast milk ⑷Feel the hassle of breastfeeding ⑸Worry about figure or image ⑹Think formula powder id more nutritious ⑺The child is ill ⑻Children refuse to suck ⑼Else，please specify ______ |
| **J07** | Do you add formula milk to your child?（1）No**（skip to J09）**（2）Yes，formula milk condition： （ times/day）， mL at every time |
| **J08** | The first time you add formula milk to your child is： month-old day |
| **J09** | Do you child currently use a bottle with nipple to drink water, milk or juice? ⑴No ⑵Yes |
| **J10** | Your child’s sleep：⑴Normal ⑵Abnormal，such as difficult to fall asleep, frequent night wake, sleep rhythm disorder） |
| **J11** | Sleep time: （ hours/day） |
| **J12** | Outdoor activities： （ hours/day） |
| **J13** | Take vitamin D：（ **IU/**day） |
| **J14** | weight： （  **Kg**） |
| **J15** | length： （ **cm**） |
| **J16** | Head circumference： （ **cm**） |
| **J17** | Number of teeth： （ ） |
| **J18** | Former fontanel： （ **cm**× **cm**） |
| **J19** | Physical examination：⑴Normal ⑵Abnormal，please specify ______ |
| **J20** | Externalia**（can be more selected）**：⑴Normal ⑵Cryptorchidism ⑶Sheath effusion ⑷Phimosis ⑸Else |
| **J21** | Arms and legs：⑴Normal ⑵Horseshoe inside and outside flip ⑶Multi-finger toe ⑷O-legged ⑸Type x legs ⑹Else |
| **J22** | Suspected rickets symptoms**（can be more selected）**：⑴No ⑵Night terrors ⑶Hyperhidrosis ⑷Irritable |
| **J23** | Rickets signs（**can be more selected）**：⑴No ⑵Skull softening ⑶Ping-pong head ⑷Square cranial ⑸Rib beads ⑹Rib flip ⑺Rib soft ditch ⑻Pigeon breast ⑼Funnel chest ⑽Bracelet ⑾Lower limb deformity ⑿Spinal curvature ⒀O-legged ⒁Type X legs ⒂Else |

# Tab M：Record of birth insprction during pregnancy

| **M01** | Number of follow-up during pregnancy times | | | | | | | | | |
| --- | --- | --- | --- | --- | --- | --- | --- | --- | --- | --- |
|  |  | **M02**  **First time** | **M03**  **Second time** | **M04**  **The third time** | **M05**  **The fourth time** | **M06**  **The fifth time** | **M07**  **The sixth time** | **M08**  **The seventh time** | **M09**  **The eighth time** | **M10**  **The ninth time** |
| **01** | Follow-up date： |  |  |  |  |  |  |  |  |  |
| **02** | Pregnancy week at follow-up： |  |  |  |  |  |  |  |  |  |
| **03** | Weight at follow-up(**Kg)**： |  |  |  |  |  |  |  |  |  |
| **04** | Fetal heart during follow-up (times/minutes)： |  |  |  |  |  |  |  |  |  |
| **05** | Blood pressure(**mmHg**)： |  |  |  |  |  |  |  |  |  |
| **06** | Uterine height(**cm**)： |  |  |  |  |  |  |  |  |  |
| **07** | Abdominal circumference(**cm**)： |  |  |  |  |  |  |  |  |  |
| **08** | Pre-pregnancy risk factors： |  |  |  |  |  |  |  |  |  |
| **09** | High risk score： |  |  |  |  |  |  |  |  |  |
| **10** | Fetal position： |  |  |  |  |  |  |  |  |  |
| **11** | Fetal presentation ⑴Not clear ⑵Head ⑶Hip position ⑷Shoulder |  |  |  |  |  |  |  |  |  |
| **12** | Fetal presentation to the basin degree：  ⑴Float ⑵Semi-fixed ⑶Fixed |  |  |  |  |  |  |  |  |  |
| **15** | Hemoglobin (**g/L**)： |  |  |  |  |  |  |  |  |  |
| **16** | Blood glucose (**mmol/L**)： |  |  |  |  |  |  |  |  |  |

# Tab A：Bsic informations

| **A01** | Mother’s name： | | |
| --- | --- | --- | --- |
| **A02** | Mother’s birth date： year month day | | |
| **A03** | Age： year-old | | |
| **A04** | Mother’s identity card number |  |  |
| **A05** | Mother’s national： | | |
| **A06** | Mother’s account address： province city | | |
| **A07** | Mother’s residential address： city district(country) street community | | |
| **A08** | Telephone number： |  |  |
| **A09** | Mother’s educational level： ⑴Primary and below ⑵Junior high school ⑶Senior/vocational/secondary school ⑷Universities/colleges ⑸Master and above ⑹Else（please specify）： | | |
| **A10** | Mother’s occupation：⑴National public servant ⑵Teacher ⑶Doctor ⑷Nurse ⑸Other professional and technical personnel ⑹Office worker ⑺Enterprise management personnel ⑻Worker ⑼Peasant ⑽Student ⑾Active army ⑿Professional ⒀Self-employed ⒁Unemployed people ⒂Retired persons ⒃Else（please specify）： | | |
| **A12** | Mother’s height： **cm** | | |
| **A15** | Father’s name： | | |
| **A16** | Father’s birth date： year month day | | |
| **A17** | Age： year-old | | |
| **A18** | Father’s identity card number： | | |
| **A19** | Father’s national： | | |
| **A20** | Father’s educational level： ⑴Primary and below ⑵Junior high school ⑶Senior/vocational/secondary school ⑷Universities/colleges ⑸Master and above ⑹Else（please specify）： | | |
| **A21** | Father’s occupation：⑴National public servant ⑵Teacher ⑶Doctor ⑷Nurse ⑸Other professional and technical personnel ⑹Office worker ⑺Enterprise management personnel ⑻Worker ⑼Peasant ⑽Student ⑾Active army ⑿Professional ⒀Self-employed ⒁Unemployed people ⒂Retired persons ⒃Else（please specify）： | | |
| **A22★** | Father’s height： **cm** | | |
| **A23★** | Father’s weight： **Kg** | | |

# Tab B：Pregnancy conditions

| **B18★** | Do you have a past history before you become pregnant?**（can be more selected）**： ⑴No ⑵Hypertension ⑶Diabetes ⑷Coronary disease ⑸Chronic obstructive pulmonary disease ⑹Stroke ⑺Severe mental illness ⑻Tuberculosis ⑼Hepatitis ⑽Cancer, please specify ⑾Other statutory infectious disease ⑿Allergic disease ⒀Occupational disease ⒁Else |
| --- | --- |
| **B19** | Did you have any surgery before this pregnancy?：⑴No ⑵Yes（please specify the number and type） |
| **B20** | Do you have a history of trauma before this pregnancy?：⑴No ⑵Yes（please specify the number and reason） |
| **B21** | Do you have a history of blood transfusion before this pregnancy?： ⑴No ⑵Yes（reason） |
| **B22** | Do you have a history of drug allergies before this pregnancy?：⑴No ⑵Yes（please specify the number and type） |
| **B23** | The way you conceived this time： ⑴Natural conception ⑵Artificial assisted reproductive technology |
| **B24** | Your pregnant times ；production times |
| **B25** | Lat menstrual date： year month day |
| **B26** | Exected date of childbirth： year month day |

# Tab C：Family history

| **C01★** | Past history of child’s father**（can be more selected）**： ⑴No ⑵Heart disease ⑶Tuberculosis ⑷Hepatopathy ⑸Nephropathy ⑹Chronic hypertension ⑺Anaemia ⑻Hematopathy ⑼Mental disease ⑽Diabetes ⑾Abnormal thyroid function ⑿History of drug addiction ⒀Allergic disease ⒁Else, please specify . |
| --- | --- |
| **C02** | Does the father of your husband suffer from the following disease?**（can be more selected）**：   1. No ⑵Hypertension ⑶Diabetes ⑷Coronary disease ⑸Chronic obstructive pulmonary disease ⑹Stroke ⑺Severe mental illness ⑻Tuberculosis ⑼Hepatitis ⑽Cancer, please specify ⑾Congenital malformations, please specify ⑿Else, please specify |
| **C03** | Does the mother of your husband suffer from the following disease?**（can be more selected）**：   1. No ⑵Hypertension ⑶Diabetes ⑷Coronary disease ⑸Chronic obstructive pulmonary disease ⑹Stroke ⑺Severe mental illness ⑻Tuberculosis ⑼Hepatitis ⑽Cancer, please specify ⑾Congenital malformations, please specify ⑿Else, please specify |
| **C04** | Does the father of you suffer from the following disease?**（can be more selected）**：   1. No ⑵Hypertension ⑶Diabetes ⑷Coronary disease ⑸Chronic obstructive pulmonary disease ⑹Stroke ⑺Severe mental illness ⑻Tuberculosis ⑼Hepatitis ⑽Cancer, please specify ⑾Congenital malformations, please specify ⑿Else, please specify |
| **C05** | Does the mother of you suffer from the following disease?**（can be more selected）**：   1. No ⑵Hypertension ⑶Diabetes ⑷Coronary disease ⑸Chronic obstructive pulmonary disease ⑹Stroke ⑺Severe mental illness ⑻Tuberculosis ⑼Hepatitis ⑽Cancer, please specify ⑾Congenital malformations, please specify ⑿Else, please specify |
| **C06** | Do you have a family history of genetic disease?： ⑴No ⑵Yes（please indicate the name of the disease） |
| **C07** | Does the child’s father have a family history of genetic disease： ⑴No ⑵Yes（please indicate the name of the disease） |
| **C08** | Are you close to your child’s father?： ⑴No ⑵Yes（please specify） . |

# Tab H：Neonatal situation questionnaire

| **H01** | Neonatal pregnancy week at birth： weeks day |  |
| --- | --- | --- |
| **H03** | Neonatal weight at birth： **Kg** |  |
| **H04** | Newborn birth length： **cm** |  |
| **H05** | The delivery method of newborns is：⑴Natural delivery（with side cutting） ⑵Natural delivery（no side cutting）⑶Cesarean （4）Accouche |  |
| **H08** | Does the newborn have deformities?:⑴No ⑵Yes，（please specify）：­­­­_______ |  |
| **H09** | Neonatal disease screening results：（1）Normal ⑵Abnormal，please specify：­­­­ _____ |  |
| **H10** | Neonatal hearing screening： ⑴Pass ⑵ ⑶Fail to pass （99）Unknown |  |
| **H11** | Neonatal asphyxia（Apgar score）： ⑴Not done ⑵Yes， score（99）Unknown |  |
