# Supplementary material for: Effect of feeding patterns on growth and nutritional status of children aged 0-24 months: A Chinese cohort study
Source: PLoS One. 2019 Nov 19;14(11):e0224968. doi: 10.1371/journal.pone.0224968 (PMC6863544; doi:10.1371/journal.pone.0224968)
Supplement: S2 Text — (ZIP) [file pone.0224968.s002.zip › 1╘┬┴Σ.docx]

2015年开福区社区母婴健康信息采集

**编号： □□□□□□**

开福区 社区卫生服务中心

居住地址： 区（县） 街道（路） 社区

母亲姓名： 联系电话：

父亲姓名： 联系电话：

子女姓名： 子女性别：

子女出生日期：

信息收集时间： 年 月 日 信息收集者：

目录

[询问部分 3](#_Toc416447862)

[表B：孕期情况 3](#_Toc416447863)

[表D: 孕产妇危险因素接触史 4](#_Toc416447864)

[表E 孕产妇孕期膳食与营养 4](#_Toc416447865)

[表F：产后情况调查表 6](#_Toc416447866)

[表G：爱丁堡产后抑郁量表（过去分娩至今的感受） 7](#_Toc416447867)

[表J：1月龄儿童随访记录表 7](#_Toc416447868)

[表K:儿童患病情况 8](#_Toc416447869)

[摘录（自填）部分 9](#_Toc416447870)

[表L：孕产妇孕期家庭居住环境情况 (注：★表示需要询问的部分) 9](#_Toc416447871)

[表M：孕期产检记录 10](#_Toc416447872)

[表A：基本信息 11](#_Toc416447873)

[表B：孕期情况 13](#_Toc416447874)

[表C：家族史 13](#_Toc416447875)

[表H：新生儿情况调查表 14](#_Toc416447876)

# 询问部分

# 表B：孕期情况

| **B01** | 目前家庭居住人数 人 | | | | | | | | | | |
| --- | --- | --- | --- | --- | --- | --- | --- | --- | --- | --- | --- |
| **B02** | 家庭人均收入(元/月)：⑴2000元及以下 ⑵2001元-5000元 ⑶5001元-10000元 ⑷ 10001元-15000元 ⑸15000元以上 | | | | | | | | | | |
| **B03** | 您分娩前的体重： Kg | | | | | | | | | | |
| **B04** | 您是否有过人工流产？（1）否**（跳至B06）** （2）是,请注明次数 ___次 | | | | | | | | | | |
| **B05** | 若有人工流产，原因是： ⑴计划生育 ⑵主观原因 ⑶胎儿异常 ⑷母体疾病 ⑸其他 | | | | | | | | | | |
| **B06** | 您本次怀孕是否有准备： ⑴否 ⑵是 | | | | | | | | | | |
| **B07** | 您本次怀孕前是否进行过宫颈癌的筛查？⑴否 ⑵是,请注明 | | | | | | | | | | |
| **B08** | 您怀孕期是否出现呕吐？： ⑴否 ⑵是，持续时间： 天 | | | | | | | |  | |  |
| **B09** | 您孕期是否出现阴道流血： ⑴否**(跳至B11)**  ⑵是，持续时间： 天 | | | | | | | |  | |  |
| **B10** | 您孕期出现阴道流血的孕期**（可多选）：** ⑴孕早期 ⑵孕中期 ⑶孕晚期 | | | | | | | |  | |  |
| **B11** | 您孕期患有以下哪些妊娠合并症**（可多选）：**  （1）无 ⑵贫血 ⑶糖尿病 ⑷原发性高血压 ⑸乙型肝炎 ⑹先天性心脏病 ⑺风湿性心脏病 ⑻高血压性心脏病 ⑼慢性肾炎 ⑽急性肾盂肾炎 ⑾甲亢 ⑿其他肾炎 ⒀其他，请注明 (99)不详 | | | | | | | | | | |
| **B12** | 您孕期患有以下哪些妊娠并发症**（可多选）：**  （1）无（2）多胎妊娠（3）妊娠高血压疾病（4）前置胎盘（5）羊水异常（6）胎儿生长受限（7）胎儿异常（8）妊娠期糖尿病（9）延期妊娠（10）过期妊娠（11）先兆早产（12）晚期先兆流产（13）死胎（14）巨大儿（15）中度贫血（16）ABO溶血（17）妊娠肝损 （18）其他，请注明 （99）不详 | | | | | | | | | | |
| **B13** | 您怀孕期间是否有过因病就医行为？ ⑴否**（跳至表D）**  ⑵是 | | | | | | | | | | |
| **B14** | 就诊次数 | 01.  就诊  孕 月 | 02.  就诊单位名称 | 03.  就诊单位类型：⑴药店⑵诊所⑶医院（4）其他 | 04.  最终诊断 | 05.  主要治疗药物 | 06.  其他处理 | 07.  疾病转归：⑴痊愈 ⑵好转⑶无变化 ⑷恶化 ⑸其他 | |  |  |
| **B15** | 第1次 |  |  |  |  |  |  |  | |  |  |
| **B16** | 第2次 |  |  |  |  |  |  |  | |  |  |
| **B17** | 第3次 |  |  |  |  |  |  |  | |  |  |

# 表D: 孕产妇危险因素接触史

| **D01** | 喂养有猫狗等宠物？（1）否 （2）是 | | |
| --- | --- | --- | --- |
| **D02** | 您孕期是否锻炼: ⑴否**（跳至D04）** ⑵是，若有则平均每天锻炼 分钟 | | |
| **D03** | 您最常用的锻炼方式是哪一种:  ⑴步行锻炼 ⑵中等强度体育锻炼（如慢跑、慢速游泳、太极拳、乒乓球、扇子舞、扭秧歌等）  ⑶大强度体育锻炼（如中速跑步、中速游泳、足球、篮球、羽毛球等） | | |
| **D04** | 您孕期家庭中的吸烟人数： 人 | | |
| **D05** | 孕期儿童父亲是否吸烟：  (1)否 ⑵每天吸1-4支 ⑶每天吸5-9支⑷每天吸10-15支 ⑸每天吸15-20支（6）每天20支以上 | | |
| **D06** | 您怀孕期是否有被动吸烟（每天超过15分钟）： （1）否 （2）是 | | |
| **D07** | 您孕期是否吸烟: ⑴否 ⑵每天吸1-4支 ⑶每天吸5-9支⑷每天吸10-15支 ⑸每天吸15-20支（6）每天20支以上 | | |
| **D08** | 您怀孕前半年内，是否饮酒？（1）否 （2）是 | | |
| **D09** | 儿童母亲怀孕前半年内，儿童父亲是否饮酒： ⑴否 ⑵是 （99）不详 | | |
| **D10** | 您孕期是否有在工作或生活中经常接触或一次大量接触以下选项**（可多选）**：   1. 否 ⑵农药 ⑶有机溶剂 ⑷噪音 ⑸电脑 ⑹手机 ⑺其他，请注明 （99）不详 | | |
| **D11** | 孕期您是否穿孕妇防护服： ⑴否 ⑵是 |  |  |

# 表E 孕产妇孕期膳食与营养

| **E01** | 您在**怀孕期间**为了胎儿健康是否愿意改变不良饮食习惯？（1）否 （2）是 | | |
| --- | --- | --- | --- |
| **E02** | 您在**孕期**为了胎儿健康是否愿意学习一些孕期相关营养知识？（1）否 （2）是 | | |
| **E03** | 您在**孕期**获得营养知识的途径为**（可多选）**： ⑴医务工作者 ⑵营养师 ⑶电视广播网络 ⑷营养书籍 ⑸长辈亲朋 ⑹营养知识讲座宣传栏 ⑺报刊杂志 ⑻其他，请注明 。 | | |
| **E04** | 您在**孕期**选择食物时最先考虑**（可多选）**？ ⑴根据自己的口味或爱好 ⑵考虑食物的营养价值 ⑶自身的营养需要 ⑷食物价格 ⑸其他，请注明 . | | |
| **E05** | 您在**孕期**是否注意提醒自己吃以下对胎儿有益的食物？   1. 否 ⑵奶类及制品（3）肉类（4）蛋类（5）坚果类（6）蔬菜水果（7）水产品（8）动物内脏（9）豆类 | | |
| **E06** | 您是否服用叶酸？（1）否（2）是，孕前 月至孕后 月 | | |
| **E07** | 您在**孕期**补充了哪些营养补充剂？**（可多选）**：   1. 未食用 ⑵钙补充剂 ⑶维生素补充剂 ⑷鱼肝油 ⑸铁补充剂 ⑹孕妇专用奶粉 ⑺其他，请注明 | | |
| **E08** | 您在孕期自感营养状况： ⑴较差 ⑵中等 ⑶良好 | | |
| **E09** | 您在现在选择食物时最先考虑**（可多选）**？  ⑴根据自己的口味或爱好 ⑵考虑食物的营养价值 ⑶自身的营养需要 ⑷食物价格 ⑸其他,请注明 | | |
| **E10** | 我们想了解您在**孕中晚期及产后1月的**食物摄入频率，见下表,请将选项填在对应的空格里。 | | |
|  | **食物名称 食用频率** | **孕中晚期**  **E11** | **孕后1月**  **E12** |
| **01** | 谷薯类： ⑴基本不吃 ⑵每月吃1~2次 ⑶每周吃1~2次 ⑷每周吃3~4次 ⑸每周吃5次及以上 |  |  |
| **02** | 奶类及制品: ⑴基本不吃 ⑵每月吃1~2次 ⑶每周吃1~2次 ⑷每周吃3~4次 ⑸每周吃5次及以上 |  |  |
| **03** | 蛋类: ⑴基本不吃 ⑵每月吃1~2次 ⑶每周吃1~2次 ⑷每周吃3~4次 ⑸每周吃5次及以上 |  |  |
| **04** | 新鲜水果: ⑴基本不吃 ⑵每月吃1~2次 ⑶每周吃1~2次 ⑷每周吃3~4次 ⑸每周吃5次及以上 |  |  |
| **05** | 新鲜蔬菜: ⑴基本不吃 ⑵每月吃1~2次 ⑶每周吃1~2次 ⑷每周吃3~4次 ⑸每周吃5次及以上 |  |  |
| **06** | 禽畜肉类: ⑴基本不吃 ⑵每月吃1~2次 ⑶每周吃1~2次 ⑷每周吃3~4次 ⑸每周吃5次及以上 |  |  |
| **07** | 动物内脏: ⑴基本不吃 ⑵每月吃1~2次 ⑶每周吃1~2次 ⑷每周吃3~4次 ⑸每周吃5次及以上 |  |  |
| **08** | 豆类及制品： ⑴基本不吃 ⑵每月吃1~2次 ⑶每周吃1~2次 ⑷每周吃3~4次 ⑸每周吃5次及以上 |  |  |
| **09** | 坚果: ⑴基本不吃 ⑵每月吃1~2次 ⑶每周吃1~2次 ⑷每周吃3~4次 ⑸每周吃5次及以上 |  |  |
| **10** | 水产品: ⑴基本不吃 ⑵每月吃1~2次 ⑶每周吃1~2次 ⑷每周吃3~4次 ⑸每周吃5次及以上 |  |  |
| **11** | 菌藻类: ⑴基本不吃 ⑵每月吃1~2次 ⑶每周吃1~2次 ⑷每周吃3~4次 ⑸每周吃5次及以上 |  |  |
| **12** | 血制品: ⑴基本不吃 ⑵每月吃1~2次 ⑶每周吃1~2次 ⑷每周吃3~4次 ⑸每周吃5次及以上 |  |  |
| **13** | 酒类: ⑴基本不吃 ⑵每月吃1~2次 ⑶每周吃1~2次 ⑷每周吃3~4次 ⑸每周吃5次及以上 |  |  |
| **14** | 饮料: ⑴基本不吃 ⑵每月吃1~2次 ⑶每周吃1~2次 ⑷每周吃3~4次 ⑸每周吃5次及以上 |  |  |
| **15** | 油炸食品: ⑴基本不吃 ⑵每月吃1~2次 ⑶每周吃1~2次 ⑷每周吃3~4次 ⑸每周吃5次及以上 |  |  |
| **16** | 腌制食品: ⑴基本不吃 ⑵每月吃1~2次 ⑶每周吃1~2次 ⑷每周吃3~4次 ⑸每周吃5次及以上 |  |  |

# 表F：产后情况调查表

| **F01** | 产时是否患有并发症**（可多选）**： ⑴否 ⑵产后出血 ⑶羊水栓塞 ⑷子宫破裂 ⑸脐带异常 ⑹其他 | |
| --- | --- | --- |
| **F02** | 孕妇分娩以后会阴是否完整： ⑴完整 ⑵切开 ⑶裂伤 | |
| **F03** | 分娩期间是否有无子宫切除：⑴否 ⑵有（请注明原因） | |
| **F04** | 您现在的体重： **Kg** | |
| **F05** | 儿童父亲现在吸烟吗： ⑴不吸烟 ⑵每天吸1-4支 ⑶每天吸5-9支⑷每天吸10-15支 ⑸每天吸15-20支（6）每天20支以上 | |
| **F06** | 儿童父亲现在是否饮酒？ ⑴否 ⑵是 | |
| **F07** | 目前，您平均每周被动吸烟的天数（每天超过15分钟）： ⑴0天 ⑵1-2天 ⑶3-5天 ⑷6-7天 | |
| **F08** | 目前，孩子平均每周被动吸烟的天数（每天超过15分钟）：⑴0天 ⑵1-2天 ⑶3-5天 ⑷6-7天 | |
| **F09** | 婴儿出生后首次吸吮奶头时间：⑴分娩后30分钟内 ⑵30-59分钟 ⑶1-2小时 ⑷3-24小时 ⑸＞24小时，请填写第 天 |  |
| **F10** | 婴儿吸吮奶头之前是否使用过奶瓶（奶嘴）： ⑴否 ⑵是 |  |
| **F11** | 孩子出生后多长时间开始喂母乳？⑴分娩后30分钟内 ⑵0.5-2小时 ⑶2-24小时 ⑷24-48小时 ⑸＞48小时，请填写第 天 |  |
| **F12** | 婴儿的主要看护人 ⑴父母亲 ⑵（外）祖父母 ⑶保姆（钟点工）⑷亲属或朋友 ⑸其他（99）不详 | |

# 表G：爱丁堡产后抑郁量表（过去分娩至今的感受）

| **3G01** | 我开心,也能看到事物有趣的一面 | （1）像以前一样 （2）不如以前多 （3）明显比以前少 （4）完全不能 |
| --- | --- | --- |
| **3G02** | 我对未来保持乐观态度 | （1）像以前一样 （2）不如以前多 （3）明显比以前少 （4）完全不能 |
| **3G03** | 当事情出错时，我毫无必要地责备我自己 | （1）从来没有 （2）偶尔这样 （3）有时候这样 （4）经常这样 |
| **3G04** | 我无缘无故感到焦虑和担心 | （1）从来没有 （2）偶尔这样 （3）有时候这样 （4）经常这样 |
| **3G05** | 我无缘无故感到惊慌和害怕 | （1）从来没有 （2）偶尔这样 （3）有时候这样 （4）经常这样 |
| **3G06** | 事情发展到我无法应付的地步 | （1）从来没有 （2）偶尔这样 （3）有时候这样 （4）经常这样 |
| **3G07** | 我因心情不好而影响睡眠 | （1）从来没有 （2）偶尔这样 （3）有时候这样 （4）经常这样 |
| **3G08** | 我感到难过和悲伤 | （1）从来没有 （2）偶尔这样 （3）有时候这样 （4）经常这样 |
| **3G09** | 我因心情不好而哭泣 | （1）从来没有 （2）偶尔这样 （3）有时候这样 （4）经常这样 |
| **3G10** | 我有伤害自己的想法 | （1）从来没有 （2）偶尔这样 （3）有时候这样 （4）经常这样 |

# 表J：1月龄儿童随访记录表

| **J01** | 检查日期： 年 月 日 |
| --- | --- |
| **J02** | 实足月龄： 月龄 天 |
| **J03** | 喂养方式：（1）纯母乳喂养（2）混合喂养 （3）人工喂养**(跳至J07)** |
| **J04** | 母乳次数： （ 次/日） |
| **J05** | 婴儿现在是否已断母乳？（1）否**(跳至J07)** （2）是，断母乳的月龄是 月 天 |
| **J06** | 您给孩子断奶的原因是什么？ **（可多选）** ⑴工作需求 ⑵生病 ⑶无母乳 ⑷觉得母乳喂养麻烦 ⑸担心影响身材或形象 ⑹认为配方粉更有营养 ⑺孩子生病 ⑻孩子拒绝吸吮 ⑼其他，请注明 ______ |
| **J07** | 您是否给孩子添加配方奶？（1）否**（跳至J09）**（2）是，配方奶情况： （ 次/天），每次 mL |
| **J08** | 您首次给孩子添加配方奶或鲜奶的时间为： 月龄 天 |
| **J09** | 目前孩子是否使用带奶嘴的奶瓶喝水、奶类或果汁等？ ⑴否 ⑵是 |
| **J10** | 孩子的睡眠：（⑴正常 ⑵异常，如入睡困难，频繁夜醒，睡眠节律紊乱） |
| **J11** | 睡眠时间: （ 小时/日） |
| **J12** | 户外活动： （ 小时/日） |
| **J13** | 服用维生素**D**：（ **IU/**日） |
| **J14** | 体重： （  **Kg**） |
| **J15** | 身长： （ **cm**） |
| **J16** | 头围： （ **cm**） |
| **J17** | 孩子的出牙数： （ 颗） |
| **J18** | 前囟： （ **cm**× **cm**） |
| **J19** | 体格检查：⑴正常 ⑵异常，请注明 ______ |
| **J20** | 外生殖器**（可多选）**：⑴正常 ⑵隐睾 ⑶鞘膜积液 ⑷包茎 ⑸其他 |
| **J21** | 四肢：⑴正常 ⑵马蹄内外翻 ⑶多指趾 ⑷O型腿 ⑸X型腿 ⑹其他 |
| **J22** | 可疑佝偻病症状**（可多选）**：⑴无 ⑵夜惊 ⑶多汗 ⑷烦躁 |
| **J23** | 佝偻病体征（**可多选）**：⑴否 ⑵颅骨软化 ⑶乒乓头 ⑷方颅 ⑸肋串珠 ⑹肋外翻 ⑺肋软沟 ⑻鸡胸 ⑼漏斗胸 ⑽手镯 ⑾下肢畸形 ⑿脊柱弯曲 ⒀ O型腿 ⒁X型腿 ⒂其他 |

# 表K:儿童患病情况

| **K01** | 您的孩子自出生到1月龄对以下哪些物质有过敏情况**（可多选）：** ⑴无 （2）牛奶 （3）奶酪 （4）全蛋 （5）尘螨 （6）家尘  （7）牛肉 （8）鱼虾蟹贝蛤 （9）狗毛鸡毛 （10）蟑螂 （11）橙（12）各类真菌 （13）黄豆 （14）各类花粉 （15）花生  （16）青胡椒 （17）小麦 （18）鸡肉 （19）蘑菇 （20）其他（请注明） （99）不详 | | | | | | | | |
| --- | --- | --- | --- | --- | --- | --- | --- | --- | --- |
| **K02** | 您的孩子自出生至1月龄有无药物过敏情况**（可多选）**：（1）无**（跳至K04）** （2）有,请注明 | | | | | | | |  |
| **K03** | 您的孩子发生药物过敏反应的给药途径：（1）静脉滴注 （2）皮下注射 （3）静脉注射（4）肌肉注射（5）口服给药（99）不详 | | | | | | | |  |
| **K04** | 您的孩子自出生至1月龄患过以下哪些过敏性疾病：  （1）无（2）过敏性皮炎（3）过敏性鼻炎（4）过敏性哮喘（5）过敏性紫癜（6）过敏性休克 （7）其他 | | | | | | | |  |
| **K05** | 您的孩子自出生至1月龄患过以下哪些传染病：  ⑴无 ⑵水痘 （3）腮腺炎 （4）风疹 （5）麻疹 （6）手足口病（7）其他传染病,请注明 （99）不详 | | | | | | | |  |
| **K06** | 您的孩子自出生至1月龄是否患过下列疾病**（可多选）：**   1. 无**（跳至K09）** ⑵感冒 ⑶腹泻 ⑷支气管炎 ⑸肺炎 ⑹哮喘 ⑺佝偻病 ⑻贫血 ⑼体弱儿 ⑽其他(请注明) | | | | | | | |  |
| **K07** | 若患过感冒，感冒次数为： 次 | | | | | | | |  |
| **K08** | 若患过腹泻，腹泻次数为： 次 | | | | | | | |  |
| **K09** | 您的孩子出生以来是否有因病就医行为： ⑴否**（跳至K14）** ⑵是 | | | | | | | |  |
|  | 就诊次数 | 01.  就诊月龄 | 02.  就诊单位名称 | 03.  就诊单位类型：⑴药店⑵诊所⑶医院（4）其他 | 04.  最终诊断 | 05.  主要治疗药物 | 06.  其他处理 | 07.  疾病转归：⑴痊愈 ⑵好转⑶无变化 ⑷恶化 ⑸其他 |  |
| **K10** | 第1次 |  |  |  |  |  |  |  |  |
| **K11** | 第2次 |  |  |  |  |  |  |  |  |
| **K12** | 第3次 |  |  |  |  |  |  |  |  |
| **K13** | 第4次 |  |  |  |  |  |  |  |  |
| **K14** | 您的孩子是否发生过意外伤害？ ⑴否**（跳至表L）** ⑵外伤（车祸、坠落、跌伤、滑倒、磕伤钝器伤等）（3）烧烫伤（4）利器伤（5）化学品损伤或中毒（6）气管异物伤或窒息（7）触电（8）动物咬伤（9）溺水（10）其他（请注明） （99）不详 | | | | | | | |  |

# 摘录（自填）部分

# 表L：孕产妇孕期家庭居住环境情况 (注：★表示需要询问的部分)

| **L01** | 您孕期家庭中房子是否租赁：（1）否 （2）是 |
| --- | --- |
| **L02** | 您孕期家庭住房类型： （1）平房 （2）普通楼房 （3）电梯房 （4）别墅 （5）其他,请注明 |
| **L03** | 您孕期家庭居住楼层： 层 |
| **L04★** | 您孕期家庭居住面积： **m^2^** |
| **L05** | 您孕期家庭住房采光： ⑴差 ⑵一般 ⑶好 |
| **L06** | 您孕期家庭住房通风： ⑴差 ⑵一般 ⑶好 |
| **L07** | 您孕期家庭住房保暖： ⑴差 ⑵一般 ⑶好 |
| **L08** | 您孕期家庭住房湿度： ⑴潮湿 ⑵适中 ⑶干燥 |
| **L09** | 您孕期家庭厨房类型： ⑴单独厨房 ⑵混用 |
| **L10★** | 您孕期家庭烹饪燃料： ⑴管道煤气（天然气） ⑵液化气 ⑶煤炭 ⑷木柴 ⑸电 ⑹其他,请注明 |
| **L11★** | 您孕期家庭排风设施**(可多选）**： ⑴否 ⑵油烟机 ⑶换气扇 ⑷烟囱 ⑸其他（请注明）： |
| **L12** | 您孕期家庭排烟情况： ⑴差 ⑵一般 ⑶好 |
| **L13★** | 您孕期家庭饮用水源**(可多选）**： ⑴自来水 ⑵井水 ⑶河水 ⑷纯水或桶装水 ⑸直饮水 ⑹其他,请注明 |
| **L14** | 您孕期家庭中的厕所类型是**(可多选）**： ⑴户外公厕 ⑵户内坑式 ⑶马桶 ⑷其他,请注明 |

# 表M：孕期产检记录

| **M01** | 孕期随访次数 次 | | | | | | | | | |
| --- | --- | --- | --- | --- | --- | --- | --- | --- | --- | --- |
|  |  | **M02**  **第1次** | **M03**  **第2次** | **M04**  **第3次** | **M05**  **第4次** | **M06**  **第5次** | **M07**  **第6次** | **M08**  **第7次** | **M09**  **第8次** | **M10**  **第9次** |
| **01** | 随访日期： |  |  |  |  |  |  |  |  |  |
| **02** | 随访时的孕周： |  |  |  |  |  |  |  |  |  |
| **03** | 孕期随访体重(**Kg)**： |  |  |  |  |  |  |  |  |  |
| **04** | 孕期随访时胎心(次/分)： |  |  |  |  |  |  |  |  |  |
| **05** | 孕期随访时血压(**mmHg**)： |  |  |  |  |  |  |  |  |  |
| **06** | 孕期随访时宫高(**cm**)： |  |  |  |  |  |  |  |  |  |
| **07** | 孕期随访时腹围(**cm**)： |  |  |  |  |  |  |  |  |  |
| **08** | 孕前高危因素： |  |  |  |  |  |  |  |  |  |
| **09** | 高危评分： |  |  |  |  |  |  |  |  |  |
| **10** | 孕期随访时胎位： |  |  |  |  |  |  |  |  |  |
| **11** | 孕期随访时先露：  ⑴不清 ⑵头 ⑶臀位 ⑷肩 |  |  |  |  |  |  |  |  |  |
| **12** | 先露入盆程度：  ⑴浮动 ⑵半固定 ⑶固定 |  |  |  |  |  |  |  |  |  |
| **13** | 孕期随访时浮肿：  ⑴未测 ⑵－ ⑶+ ⑷++ ⑸+++ |  |  |  |  |  |  |  |  |  |
| **14** | 孕产妇孕期随访时尿蛋白：  ⑴未测 ⑵－ ⑶+ ⑷++ ⑸+++ |  |  |  |  |  |  |  |  |  |
| **15** | 孕期随访时血红蛋白(**g/L**)： |  |  |  |  |  |  |  |  |  |
| **16** | 孕期随访时血糖(**mmol/L**)： |  |  |  |  |  |  |  |  |  |
| **17** | 孕期随访时主诉： |  |  |  |  |  |  |  |  |  |
| **18** | 孕期随访时处理： |  |  |  |  |  |  |  |  |  |
| **19** | 孕期随访时检查医院： |  |  |  |  |  |  |  |  |  |
| **20** | 孕期随访时其他检查（唐氏筛查）：  ⑴未查 ⑵正常 ⑶异常,请注明 |  |  |  |  |  |  |  |  |  |
| **21** | 孕期随访时四维彩超结果：  ⑴未查 ⑵正常 ⑶异常,请注明 |  |  |  |  |  |  |  |  |  |
| **22** | 产前诊断超声结果：  ⑴未查 ⑵正常 ⑶异常,请注明 |  |  |  |  |  |  |  |  |  |
| **23** | 胎儿染色体分析结果：  ⑴未查 ⑵正常 ⑶异常,请注明 |  |  |  |  |  |  |  |  |  |
| **24** | 产检是否正常？  （1）正常（2）异常，请注明 |  |  |  |  |  |  |  |  |  |

# 表A：基本信息

| **A01** | 母亲姓名： | | |
| --- | --- | --- | --- |
| **A02** | 母亲出生日期： 年 月 日 | | |
| **A03** | 年龄： 岁 | | |
| **A04** | 母亲身份证号 |  |  |
| **A05** | 母亲民族： | | |
| **A06** | 母亲户口地址： 省 市 | | |
| **A07** | 母亲的居住地址： 市 区（县） 街道 社区 | | |
| **A08** | 联系方式：电话 |  |  |
| **A09** | 母亲的文化程度： ⑴小学及以下 ⑵初中 ⑶高中/职高/中专 ⑷大学、大专 ⑸硕士及以上 ⑹其他（请注明）： | | |
| **A10** | 母亲的职业：⑴国家公务员 ⑵教师 ⑶医生 ⑷护士 ⑸其他专业技术人员 ⑹职员 ⑺企业管理人员 ⑻工人 ⑼农民 ⑽学生 ⑾现役军人 ⑿自由职业者 ⒀个体经营者 ⒁无业人员 ⒂离退休人员 ⒃其他（请注明）： | | |
| **A11** | 母亲的医疗保险：⑴城镇职工基本医疗保险 ⑵城镇居民基本医疗保险 ⑶新型农村合作医疗 ⑷贫困救助 ⑸商业医疗保险⑹全公费 ⑺全自费 ⑻其他（请注明）： | | |
| **A12** | 母亲的身高： **cm** | | |
| **A13** | 母亲的ABO血型： ⑴A型 ⑵B型 ⑶O型 ⑷AB型 (99)不详 | | |
| **A14** | 母亲的RH血型： ⑴阴性 ⑵阳性(99)不详 | | |
| **A15** | 父亲姓名： | | |
| **A16** | 父亲出生日期： 年 月 日 | | |
| **A17** | 年龄： 岁 | | |
| **A18** | 父亲身份证号： | | |
| **A19** | 父亲民族： | | |
| **A20** | 父亲的文化程度： ⑴小学及以下 ⑵初中 ⑶高中/职高/中专 ⑷大学、大专 ⑸硕士及以上 ⑹其他（请注明）： | | |
| **A21** | 父亲的职业： ⑴国家公务员 ⑵教师 ⑶医生 ⑷护士 ⑸其他专业技术人员 ⑹职员 ⑺企业管理人员 ⑻工人 ⑼农民 ⑽学生 ⑾现役军人 ⑿自由职业者 ⒀个体经营者 ⒁无业人员 ⒂离退休人员 ⒃其他（请注明）： | | |
| **A22★** | 儿童父亲身高： **cm** | | |
| **A23★** | 儿童父亲体重： **Kg** | | |
| **A24** | ABO血型： ⑴A型 ⑵B型 ⑶O型 ⑷AB型 (99)不详 | | |
| **A25** | RH血型： ⑴阴性 ⑵阳性 (99)不详 | | |

# 表B：孕期情况

| **B18★** | 您在怀孕前是否具有既往史**（可多选）**： ⑴否 ⑵高血压 ⑶糖尿病 ⑷冠心病 ⑸慢性阻塞性肺疾病 ⑹恶性肿瘤 ⑺脑卒中 ⑻重性精神疾病 ⑼结核病 ⑽肝炎 ⑾其他法定传染病 ⑿过敏性疾病 ⒀职业病 ⒁其他 |
| --- | --- |
| **B19** | 您本次怀孕前是否做过手术：⑴否 ⑵是（请注明次数和类型） |
| **B20** | 您本次怀孕前是否具有外伤史：⑴否 ⑵是（请注明次数和原因） |
| **B21** | 您本次怀孕前是否输过血： ⑴否 ⑵是（原因） |
| **B22** | 您本次怀孕前是否有药物过敏史：⑴否 ⑵是（请注明次数和类型） |
| **B23** | 您本次受孕的方式： ⑴自然受孕 ⑵人工辅助生殖技术 |
| **B24** | 您的孕次 次；产次 次 |
| **B25** | 末次月经日期： 年 月 日 |
| **B26** | 预产期： 年 月 日 |

# 表C：家族史

| **C01★** | 儿童**父亲**既往史**（可多选）**： ⑴否 ⑵心脏病 ⑶结核病 ⑷肝病 ⑸肾病 ⑹慢性高血压 ⑺贫血 ⑻血液病 ⑼精神病 ⑽糖尿病 ⑾甲状腺功能异常 ⑿药物成瘾史 ⒀过敏性疾病 ⒁其他,请注明 . |
| --- | --- |
| **C02** | 儿童**爷爷**是否患有以下疾病**（可多选）**：   1. 否 ⑵高血压 ⑶糖尿病 ⑷冠心病 ⑸慢性阻塞性肺疾病 ⑹脑卒中 ⑺重性精神疾病 ⑻结核病 ⑼肝炎 ⑽恶性肿瘤,请注明 ⑾先天畸形,请注明 ⑿其他,请注明 |
| **C03** | 儿童**奶奶**是否患有以下疾病**（可多选）**：   1. 否 ⑵高血压 ⑶糖尿病 ⑷冠心病 ⑸慢性阻塞性肺疾病 ⑹脑卒中 ⑺重性精神疾病 ⑻结核病 ⑼肝炎 ⑽恶性肿瘤,请注明 ⑾先天畸形,请注明 ⑿其他,请注明 |
| **C04** | 儿童**外公**是否患有以下疾病**（可多选）**：   1. 否 ⑵高血压 ⑶糖尿病 ⑷冠心病 ⑸慢性阻塞性肺疾病 ⑹脑卒中 ⑺重性精神疾病 ⑻结核病 ⑼肝炎 ⑽恶性肿瘤,请注明 ⑾先天畸形,请注明 ⑿其他,请注明 |
| **C05** | 儿童**外婆**是否患有以下疾病**（可多选）**：   1. 否 ⑵高血压 ⑶糖尿病 ⑷冠心病 ⑸慢性阻塞性肺疾病 ⑹脑卒中 ⑺重性精神疾病 ⑻结核病 ⑼肝炎 ⑽恶性肿瘤,请注明 ⑾先天畸形,请注明 ⑿其他,请注明 |
| **C06** | 您是否有遗传病家族史： ⑴否 ⑵是（请注明疾病名称） |
| **C07** | 儿童父亲是否有遗传病家族史： ⑴否 ⑵是（请注明疾病名称） |
| **C08** | 您与孩子父亲是否为近亲： ⑴否 ⑵是（请具体注明） . |

# 表H：新生儿情况调查表

| **H01** | 新生儿出生时孕周： 周 天 |  |
| --- | --- | --- |
| **H02** | 助产机构： 医院/妇幼保健院 |  |
| **H03** | 新生儿出生时体重： **Kg** |  |
| **H04** | 新生儿出生身长： **cm** |  |
| **H05** | 新生儿的分娩方式是： ⑴自然分娩（有侧切） ⑵自然分娩（无侧切）⑶剖宫产（4）助产（抬头吸引口、产前助产口、臀牵引口） |  |
| **H06★** | 请问剖宫产原因**（可多选）**：  ⑴害怕疼痛 ⑵认为剖宫产更安全 ⑶维持身材 ⑷医学指征（医生建议） ⑸社会原因 ⑹其他 |  |
| **H07** | 若为医学指征（医生建议），则理由是**（可多选）**：   1. 头盆不称 ⑵胎儿宫内窘迫 ⑶脐带绕颈 ⑷臀位 ⑸过期产 ⑹胎膜早破 ⑺产程延长 ⑻巨大儿 ⑼高龄初产妇 ⑽妊娠合并内科疾病 ⑾双胎 ⑿前置胎盘 ⒀胎盘植入 ⒁胎盘早剥 ⒂横位 ⒃羊水异常 ⒄妊娠合并妇科肿瘤 ⒅其他   请注明 （99）不详 |  |
| **H08** | 新生儿是否有畸形：⑴否 ⑵是，（请注明）：­­­­_______ |  |
| **H09** | 新生儿疾病筛查结果：（1）正常 ⑵异常，请注明：­­­­ _____ |  |
| **H10** | 新生儿听力筛查： ⑴通过 ⑵未通过 ⑶未筛查 （99）不详 |  |
| **H11** | 新生儿窒息（Apgar评分）： ⑴未做 ⑵是， 分（99）不详 |  |
