# Supplementary material for: Effect of feeding patterns on growth and nutritional status of children aged 0-24 months: A Chinese cohort study
Source: PLoS One. 2019 Nov 19;14(11):e0224968. doi: 10.1371/journal.pone.0224968 (PMC6863544; doi:10.1371/journal.pone.0224968)
Supplement: S2 Text — (ZIP) [file pone.0224968.s002.zip › 3 months old.docx]

**2015 Kaifu District Community Maternal and child health information collection（3 month-old）**

**number： □□□□□□**

Kaifu District Community Health Service center

Residential Address： District(country) Street(road) Community

Mother’s name： Telephone number：

Father’s name： Telephone number：

Name of child： Gender of child：

Date of birth of the child：

Information collection time： year month day Information collector：

#

# Tab 3D：Follow-up record form for children aged 3 months

| **3D01** | Check date： year month day |
| --- | --- |
| **3D02** | Real term age： month-old day |
| **3D03** | Feeding style：（1）Exclusive breastfeeding（2）Mixed feeding （3）Formula feeding **(skip to 3D07)** |
| **3D04** | Number of breast milk： （ times/day） |
| **3D05** | Is the baby now out of breast milk？（1）No **(skip to 3D07)** （2）Yes，the month age of breast milk disconnection is month-old day |
| **3D06** | What the reason why you weaned your child? **（can be more selected）**⑴Job requirements ⑵Fall ill ⑶No breast milk ⑷Feel the hassle of breastfeeding ⑸Worry about figure or image ⑹Think formula powder id more nutritious ⑺The child is ill ⑻Children refuse to suck ⑼Else，please specify |
| **3D07** | Do you child currently use a bottle with nipple to drink water, milk or juice? ⑴No ⑵Yes |
| **3D08** | Do you add formula milk to your child?（1）No**（skip to 3D10）**（2）Yes，the first time you add formula milk to your child is： month-old day |
| **3D09** | Formula milk feeding situation： （ times/day）， ml at every time |
| **3D10** | Your child’s sleep：⑴Normal ⑵Abnormal，such as difficult to fall asleep, frequent night wake, sleep rhythm disorder） |
| **3D11** | Sleep time: （ hours/day） |
| **3D12** | Outdoor activities： （ hours/day） |
| **3D13** | Take vitamin D：（ **IU/**day） |
| **3D14** | weight： （  **Kg**） |
| **3D15** | length： （ **cm**） |
| **3D16** | Head circumference： （ **cm**） |
| **3D17** | Number of teeth： （ ） |
| **3D18** | Former fontanel： （ **cm**× **cm**） |
| **3D19** | Physical examination：⑴Normal ⑵Abnormal，please specify ______ |
| **3D20** | Externalia**（can be more selected）**：⑴Normal ⑵Cryptorchidism ⑶Sheath effusion ⑷Phimosis ⑸Else |
| **3D21** | Arms and legs：⑴Normal ⑵Horseshoe inside and outside flip ⑶Multi-finger toe ⑷O-legged ⑸Type x legs ⑹Else |
| **3D22** | Suspected rickets symptoms**（can be more selected）**：⑴No ⑵Night terrors ⑶Hyperhidrosis ⑷Irritable |
| **3D23** | Your child’s sleep：⑴Normal ⑵Abnormal，such as difficult to fall asleep, frequent night wake, sleep rhythm disorder） |
