# Supplementary material for: Effect of feeding patterns on growth and nutritional status of children aged 0-24 months: A Chinese cohort study
Source: PLoS One. 2019 Nov 19;14(11):e0224968. doi: 10.1371/journal.pone.0224968 (PMC6863544; doi:10.1371/journal.pone.0224968)
Supplement: S2 Text — (ZIP) [file pone.0224968.s002.zip › 3╘┬┴Σ.docx]

2015年开福区社区母婴健康信息采集（3月龄）

**编号： □□□□□□**

开福区 社区卫生服务中心

居住地址： 区（县） 街道（路） 社区

母亲姓名： 联系电话：

父亲姓名： 联系电话：

子女姓名： 子女性别：

子女出生日期：

信息收集时间： 年 月 日 信息收集者：

目录

[3月龄调查表 3](#_Toc417993497)

[表3A：产妇产后营养与膳食情况调查表 3](#_Toc417993498)

[表3B：产后情况调查表 3](#_Toc417993499)

[表3C：爱丁堡产后抑郁量表（过去分娩至今的感受） 4](#_Toc417993500)

[表3D：3月龄儿童随访记录表 5](#_Toc417993501)

[表3E：儿童患病情况 6](#_Toc417993502)

[表3F：婴儿添加辅食情况表 7](#_Toc417993503)

# 3月龄调查表

# 表3A：产妇产后营养与膳食情况调查表

| **3A01** | 您在现在选择食物时最先考虑**（可多选）**？  ⑴根据自己的口味或爱好 ⑵考虑食物的营养价值 ⑶自身的营养需要 ⑷食物价格 ⑸其他（请注明）： . |  |
| --- | --- | --- |
| **3A02** | 食物名称 食用频率 |  |
| **01** | 谷薯类: ⑴基本不吃 ⑵每月吃1~2次 ⑶每周吃1~2次 ⑷每周吃3~4次 ⑸每周吃≥5次 |  |
| **02** | 奶类及制品: ⑴基本不吃 ⑵每月吃1~2次 ⑶每周吃1~2次 ⑷每周吃3~4次 ⑸每周吃≥5次 |  |
| **03** | 蛋类: ⑴基本不吃 ⑵每月吃1~2次 ⑶每周吃1~2次 ⑷每周吃3~4次 ⑸每周吃≥5次 |  |
| **04** | 新鲜水果: ⑴基本不吃 ⑵每月吃1~2次 ⑶每周吃1~2次 ⑷每周吃3~4次 ⑸每周吃≥5次 |  |
| **05** | 新鲜蔬菜: ⑴基本不吃 ⑵每月吃1~2次 ⑶每周吃1~2次 ⑷每周吃3~4次 ⑸每周吃≥5次 |  |
| **06** | 禽畜肉类: ⑴基本不吃 ⑵每月吃1~2次 ⑶每周吃1~2次 ⑷每周吃3~4次 ⑸每周吃≥5次 |  |
| **07** | 豆类及制品 ⑴基本不吃 ⑵每月吃1~2次 ⑶每周吃1~2次 ⑷每周吃3~4次 ⑸每周吃≥5次 |  |
| **08** | 动物内脏: ⑴基本不吃 ⑵每月吃1~2次 ⑶每周吃1~2次 ⑷每周吃3~4次 ⑸每周吃≥5次 |  |
| **09** | 坚果: ⑴基本不吃 ⑵每月吃1~2次 ⑶每周吃1~2次 ⑷每周吃3~4次 ⑸每周吃≥5次 |  |
| **10** | 水产品: ⑴基本不吃 ⑵每月吃1~2次 ⑶每周吃1~2次 ⑷每周吃3~4次 ⑸每周吃≥5次 |  |
| **11** | 菌藻类: ⑴基本不吃 ⑵每月吃1~2次 ⑶每周吃1~2次 ⑷每周吃3~4次 ⑸每周吃≥5次 |  |
| **12** | 血制品: ⑴基本不吃 ⑵每月吃1~2次 ⑶每周吃1~2次 ⑷每周吃3~4次 ⑸每周吃≥5次 |  |
| **13** | 酒类: ⑴基本不吃 ⑵每月吃1~2次 ⑶每周吃1~2次 ⑷每周吃3~4次 ⑸每周吃≥5次 |  |
| **14** | 饮料: ⑴基本不吃 ⑵每月吃1~2次 ⑶每周吃1~2次 ⑷每周吃3~4次 ⑸每周吃≥5次 |  |
| **15** | 油炸食品: ⑴基本不吃 ⑵每月吃1~2次 ⑶每周吃1~2次 ⑷每周吃3~4次 ⑸每周吃≥5次 |  |
| **16** | 腌制食品: ⑴基本不吃 ⑵每月吃1~2次 ⑶每周吃1~2次 ⑷每周吃3~4次 ⑸每周吃≥5次 |  |

# 表3B：产后情况调查表

| **3B01** | 您产后3月的体重： Kg |
| --- | --- |
| **3B02** | 目前家庭人均月收入： 元 |
| **3B03** | 您现在是否锻炼: ⑴否**(跳至3B05)**  ⑵是，平均每天锻炼 分钟 |
| **3B04** | 您最常用的锻炼方式是哪一种: ⑴步行锻炼 ⑵中等强度体育锻炼（如慢跑、慢速游泳、太极拳、乒乓球、扇子舞、扭秧歌等）  ⑶大强度体育锻炼（如中速跑步、中速游泳、足球、篮球、羽毛球等） |
| **3B05** | 目前家庭中的吸烟人数： 人 |
| **3B06** | 儿童父亲现在吸烟吗： ⑴不吸烟 ⑵每天吸烟 ⑶不是每天吸烟 |
| **3B07** | 儿童父亲现在是否饮酒？ ⑴否 ⑵是 |
| **3B08** | 您现在是否吸烟: ⑴不吸烟 ⑵每天吸烟 ⑶不是每天吸烟 |
| **3B09** | 您现在是否饮酒？ ⑴否 ⑵是 |
| **3B10** | 目前，您平均每周被动吸烟的天数（每天超过15分钟）： ⑴0天 ⑵1-2天 ⑶3-5天 ⑷6-7天 （99）不详 |
| **3B11** | 目前，孩子平均每周被动吸烟的天数（每天超过15分钟）： ⑴0天 ⑵1-2天 ⑶3-5天 ⑷6-7天 （99）不详 |

# 表3C：爱丁堡产后抑郁量表（过去分娩至今的感受）

| **3C01** | 我开心,也能看到事物有趣的一面 | （1）像以前一样 （2）不如以前多 （3）明显比以前少 （4）完全不能 |
| --- | --- | --- |
| **3C02** | 我对未来保持乐观态度 | （1）像以前一样 （2）不如以前多 （3）明显比以前少 （4）完全不能 |
| **3C03** | 当事情出错时，我毫无必要地责备我自己 | （1）从来没有 （2）偶尔这样 （3）有时候这样 （4）经常这样 |
| **3C04** | 我无缘无故感到焦虑和担心 | （1）从来没有 （2）偶尔这样 （3）有时候这样 （4）经常这样 |
| **3C05** | 我无缘无故感到惊慌和害怕 | （1）从来没有 （2）偶尔这样 （3）有时候这样 （4）经常这样 |
| **3C06** | 事情发展到我无法应付的地步 | （1）从来没有 （2）偶尔这样 （3）有时候这样 （4）经常这样 |
| **3C07** | 我因心情不好而影响睡眠 | （1）从来没有 （2）偶尔这样 （3）有时候这样 （4）经常这样 |
| **3C08** | 我感到难过和悲伤 | （1）从来没有 （2）偶尔这样 （3）有时候这样 （4）经常这样 |
| **3C09** | 我因心情不好而哭泣 | （1）从来没有 （2）偶尔这样 （3）有时候这样 （4）经常这样 |
| **3C10** | 我有伤害自己的想法 | （1）从来没有 （2）偶尔这样 （3）有时候这样 （4）经常这样 |

# 表3D：3月龄儿童随访记录表

| **3D01** | 检查日期： （ 年 月 日） |
| --- | --- |
| **3D02** | 实足月龄： （ 月龄 天） |
| **3D03** | 喂养方式：（1）纯母乳喂养 （2）人工喂养**（跳至3D07）** （3）混合喂养 |
| **3D04** | 母乳次数： （ 次/日） |
| **3D05** | 婴儿是否已断母乳？（1）否**（跳至3D07）**  （2）是，断母乳的月龄是 月 天 |
| **3D06** | 您给孩子断奶的原因是什么？**（可多选）** ⑴工作需求 ⑵生病 ⑶无母乳 ⑷觉得母乳喂养麻烦 ⑸担心影响身材或形象 ⑹认为配方粉更有营养 ⑺孩子生病 ⑻孩子拒绝吸吮 ⑼其他，（请注明）： |
| **3D07** | 目前孩子是否使用带奶嘴的奶瓶喝水、奶类或果汁等？ ⑴否 ⑵是 |
| **3D08** | 目前您是否给孩子添加配方奶或鲜奶？（1）未添加**（跳至3D10）** （2）已添加，首次添加时间为： 月龄 天 |
| **3D09** | 配方奶情况： （ 次/天），每次 **ml** |
| **3D10** | 孩子的睡眠：（⑴正常 ⑵异常，如入睡困难，频繁夜醒，睡眠节律紊乱） |
| **3D11** | 睡眠时间: （ 小时/日） |
| **3D12** | 户外活动： （ 小时/日） |
| **3D13** | 服用维生素**D**：（ **IU**/日） |
| **3D14** | 体重： （ **Kg**） |
| **3D15** | 身长： （ **cm**） |
| **3D16** | 头围： （ **cm**） |
| **3D17** | 孩子的出牙数： （ 颗） |
| **3D18** | 前囟： （ **cm**× **cm**） |
| **3D19** | 体格检查：⑴正常 ⑵异常,请注明 ­­­________ |
| **3D20** | 外生殖器**（可多选）**：⑴正常 ⑵睾丸下降 ⑶鞘膜积液 ⑷包茎 ⑸其他 |
| **3D21** | 四肢**（可多选）**：⑴正常 ⑵马蹄内外翻 ⑶多指趾 ⑷O型腿 ⑸X型腿 ⑹其他 |
| **3D22** | 可疑佝偻病症状**（可多选）**：⑴无 ⑵夜惊 ⑶多汗 ⑷烦躁 |
| **3D23** | 佝偻病体征**（可多选）**：⑴否 ⑵颅骨软化 ⑶乒乓头 ⑷方颅 ⑸肋串珠 ⑹肋外翻 ⑺肋软沟 ⑻鸡胸 ⑼漏斗胸 ⑽手镯 ⑾下肢畸形 ⑿脊柱弯曲 ⒀ O型腿 ⒁X型腿 ⒂其他 |

# 表3E：儿童患病情况

| **3E01** | 您的孩子自出生到1月龄对以下哪些物质有过敏情况**（可多选）**： ⑴无 （2）牛奶 （3）奶酪 （4）全蛋 （5）尘螨 （6）家尘  （7）牛肉 （8）鱼虾蟹贝蛤 （9）狗毛鸡毛 （10）蟑螂 （11）橙（12）各类真菌 （13）黄豆 （14）各类花粉 （15）花生  （16）青胡椒 （17）小麦 （18）鸡肉 （19）蘑菇 （20）其他（请注明） （99）不详 | | | | | | | | |
| --- | --- | --- | --- | --- | --- | --- | --- | --- | --- |
| **3E02** | 您的孩子1月龄到3月龄有无药物过敏情况**（可多选）**：（1）无**（跳至3E04）**（2）有（请注明） | | | | | | | | |
| **3E03** | 您的孩子发生药物过敏反应的给药途径：（1）静脉滴注 （2）皮下注射 （3）静脉注射（4）肌肉注射（5）口服给药（99）不详 | | | | | | | |  |
| **3E04** | 您的孩子1月龄到3月龄患过以下哪些过敏性疾病：  （1）无（2）过敏性皮炎 （3）过敏性鼻炎（4）过敏性哮喘（5过敏性紫癜（6）过敏性休克 （7）其他 | | | | | | | |  |
| **3E05** | 您的孩子1月龄到3月龄患过以下哪些传染病：  ⑴无 ⑵水痘 （3）腮腺炎 （4）风疹 （5）麻疹 （6）手足口病 （7）其他传染病（请注明） （99）不详 | | | | | | | |  |
| **3E06** | 您的孩子1月龄到3月龄是否患过下列疾病**（可多选）：**   1. 无 ⑵感冒 ⑶腹泻 ⑷支气管炎 ⑸肺炎 ⑹哮喘 ⑺佝偻病 ⑻贫血 ⑼体弱儿 ⑽其他(请注明) | | | | | | | |  |
| **3E07** | 若患过感冒，感冒次数为： 次 | | | | | | | |  |
| **3E08** | 若患过腹泻，腹泻次数为： 次 | | | | | | | |  |
| **3E09** | 您的孩子1月龄到3月龄是否有因病就医行为： ⑴否**（跳至3E14）** ⑵是 | | | | | | | |  |
|  | 就诊次数 | 01.  就诊月龄 | 02.  就诊单位名称 | 03.  就诊单位类型：⑴药店⑵诊所⑶医院（4）其他 | 04.  最终诊断 | 05.  主要治疗药物 | 06.  其他处理 | 07.  疾病转归：⑴痊愈 ⑵好转⑶无变化 ⑷恶化 ⑸其他 | |
| **3E10** | 第1次 |  |  |  |  |  |  |  | |
| **3E11** | 第2次 |  |  |  |  |  |  |  | |
| **3E12** | 第3次 |  |  |  |  |  |  |  | |
| **3E13** | 第4次 |  |  |  |  |  |  |  | |
| **3E14** | 您的孩子是否发生过意外伤害？ ⑴否**（跳至表3F）** ⑵外伤（车祸、坠落、跌伤、滑倒、磕伤钝器伤等）（3）烧烫伤（4）利器伤（5）化学品损伤或中毒（6）气管异物伤或窒息（7）触电（8）动物咬伤（9）溺水（10）其他（请注明） （99）不详 | | | | | | | | |

# 表3F：婴儿添加辅食情况表

| **3F01** | 目前孩子是否已经添加辅食：⑴否 ⑵是，添加辅食的月龄 月 | |  |  |
| --- | --- | --- | --- | --- |
|  | **食物品种** | **食用频率** | **3F02**  **食用频率** | **3F03**  **开始添加月龄** |
| **01** | 谷薯类: | ⑴未添加 ⑵每天吃1次 ⑶每天吃≥2次 |  |  |
| **02** | 奶类及制品: | ⑴未添加 ⑵每月吃1~2次 ⑶每周吃1~2次 ⑷每周吃3~4次 ⑸每周吃≥5次 |  |  |
| **03** | 蛋类: | ⑴未添加 ⑵每月吃1~2次 ⑶每周吃1~2次 ⑷每周吃3~4次 ⑸每周吃≥5次 |  |  |
| **04** | 禽畜肉类: | ⑴未添加 ⑵每月吃1~2次 ⑶每周吃1~2次 ⑷每周吃3~4次 ⑸每周吃≥5次 |  |  |
| **05** | 鱼类: | ⑴未添加 ⑵每月吃1~2次 ⑶每周吃1~2次 ⑷每周吃3~4次 ⑸每周吃≥5次 |  |  |
| **06** | 豆类及制品 | ⑴未添加 ⑵每月吃1~2次 ⑶每周吃1~2次 ⑷每周吃3~4次 ⑸每周吃≥5次 |  |  |
| **07** | 新鲜水果: | ⑴未添加 ⑵每月吃1~2次 ⑶每周吃1~2次 ⑷每周吃3~4次 ⑸每周吃≥5次 |  |  |
| **08** | 新鲜蔬菜: | ⑴未添加 ⑵每月吃1~2次 ⑶每周吃1~2次 ⑷每周吃3~4次 ⑸每周吃≥5次 |  |  |
| **09** | 动物内脏: | ⑴未添加 ⑵每月吃1~2次 ⑶每周吃1~2次 ⑷每周吃3~4次 ⑸每周吃≥5次 |  |  |
| **10** | 坚果: | ⑴未添加 ⑵每月吃1~2次 ⑶每周吃1~2次 ⑷每周吃3~4次 ⑸每周吃≥5次 |  |  |
| **11** | 食用油： | ⑴未添加 ⑵每月吃1~2次 ⑶每周吃1~2次 ⑷每周吃3~4次 ⑸每周吃≥5次 |  |  |
