# Supplementary material for: Effect of feeding patterns on growth and nutritional status of children aged 0-24 months: A Chinese cohort study
Source: PLoS One. 2019 Nov 19;14(11):e0224968. doi: 10.1371/journal.pone.0224968 (PMC6863544; doi:10.1371/journal.pone.0224968)
Supplement: S2 Text — (ZIP) [file pone.0224968.s002.zip › 12 months old.doc]

**2015 Kaifu District Community Maternal and child health information collection（12 month-old）**

**number： □□□□□□**

Kaifu District	Community Health Service center

Residential Address：	District(country)	Street(road)	Community

Mother’s name：	Telephone number：

Father’s name：	Telephone number：

Name of child：	Gender of child：

Date of birth of the child：

Information collection time：	year	month	day	Information collector：

# Tab 12A：Follow-up record form for children aged 12 months

Check date：	year	month	day

Real term age：	month-old	day

Feeding style：（1）Breastfeeding（2）Mixed feeding （3）Formula feeding

Number of breast milk： （	times/day）

Is the baby now out of breast milk？（1）No （2）Yes，the month age of breast milk disconnection is	month-old	day

What the reason why you weaned your child? **（ can be more selected）**⑴Job requirements ⑵Fall ill ⑶No breast milk ⑷Feel the hassle of breastfeeding ⑸Worry about figure or image ⑹Think formula powder id more nutritious ⑺The child is ill ⑻Children refuse to suck ⑼Else，please specify

How long are you going to breastfeed？	month

Do you child currently use a bottle with nipple to drink water, milk or juice? ⑴No ⑵Yes

Do you add formula milk to your child?（1）No（2）Yes，the first time you add formula milk to your child is：	month-old	day

Formula milk feeding situation： （	times/day），	ml at every time

Outdoor activities： （	hours/day）

weight：	（	**Kg**）

length：	（	**cm**）

Head circumference： （	**cm**）

Number of teeth： （	）

Former fontanel：	（	**cm**×	**cm**）

Physical examination：⑴Normal ⑵Abnormal，please specify	______
