# Supplementary material for: Effect of feeding patterns on growth and nutritional status of children aged 0-24 months: A Chinese cohort study
Source: PLoS One. 2019 Nov 19;14(11):e0224968. doi: 10.1371/journal.pone.0224968 (PMC6863544; doi:10.1371/journal.pone.0224968)
Supplement: S2 Text — (ZIP) [file pone.0224968.s002.zip › 12╘┬┴Σ.doc]

**编号： □□□□□□**

**2015年开福区社区母婴健康信息采集（12月龄）**

长沙市开福区	社区卫生服务中心

居住地址：	区（县）	街道（路）	社区

母亲姓名：	联系电话：

父亲姓名：	联系电话：

子女姓名：	子女性别：

子女出生日期：

信息收集时间：	年	月	日	信息收集人：	信息核查人：

目录

表12A：12月龄儿童随访记录表	3

表12B:儿童睡眠、视力、口腔等情况调查表	4

表12C：儿童患病情况	4

表12D：宝宝添加辅食情况表	6

表12E： 儿童环境危险因素调查表	7

表12F：儿童计划免疫表	7

表12G：0~1岁宝宝家庭养育环境量表	8

表12H：0~2岁儿童父母育儿评估量表	9

# 表12A：12月龄儿童随访记录表

| **12A01** | 实足月龄： （ 月龄 天） |  |
| --- | --- | --- |
| **12A02** | 喂养方式： （1）纯母乳喂养 （2）人工喂养**（跳至12A08）** （3）混合喂养 |  |
| **12A03** | 母乳次数： （ 次/日） |  |
| **12A04** | 宝宝是否已断母乳？（1）否**（跳至12A08）** （2）是，断母乳的月龄是 月 天 |  |
| **12A05** | 您给宝宝断奶的原因是什么？**（可多选，任一选项均跳至12A08）** （1）工作需求 （2）生病 ⑶无母乳 （4）觉得母乳喂养麻烦 （5）担心影响身材或形象 ⑹认为配方奶粉更有营养 ⑺宝宝生病 ⑻宝宝拒绝吸吮 ⑼其他，（请注明）： |  |
| **12A06** | 打算母乳喂养多久？ 月 |  |
| **12A07** | 目前宝宝是否使用带奶嘴的奶瓶喝水、奶类或果汁等？ （1）否 （2）是 |  |
| **12A08** | 目前您是否给宝宝添加配方奶粉或鲜奶？ （1）未添加**（跳至12A10）** （2）已添加，首次添加时间为： 月龄 天 |  |
| **12A09** | 配方奶粉或鲜奶情况： （ 次/天），每次 **ml** |  |
| **12A10** | 宝宝是否做过微量元素的检测？（1）否**（跳至12A12）** （2）是,请注明月龄： |  |
| **12A11** | 微量元素检测结果是否正常？（1）正常 （2）缺钙 （3）缺铁 （4）缺锌 （5）缺镁 （6）铜超标 （7）铅超标 （8）其他 |  |
| **12A12** | 宝宝是否补充钙剂？（1）否**（跳至12A14）** （2）是，首次添加时间为： 月龄 天 |  |
| **12A13** | 目前，宝宝补充钙剂的频率：（1）基本不吃 （2）每月吃1~2次 （3）每周吃1~2次 （4）每周吃3~4次 （5）每周吃≥5次 |  |
| **12A14** | 目前，宝宝服用维生素**D**：（1）基本不吃 （2）每月吃1~2次 （3）每周吃1~2次 （4）每周吃3~4次 （5）每周吃≥5次 |  |
| **12A15** | 宝宝是否补充其他营养补充剂? （1）否（2）是,请注明： |  |
| **12A16** | 目前，宝宝平均每周户外活动的天数：（1）0天 （2）1-2天 （3）3-5天 （4）6-7天 （99）不详 |  |
| **12A17** | 宝宝平均每天户外活动的时间： 小时/日 |  |
| **12A18** | 宝宝父母是否参加过育儿班？（1）否 （2）是 |  |
| **12A19** | 是否带宝宝参加过早教班？（1）否 （2）是 |  |
| **12A20** | 是否给宝宝做耐寒训练？（1）否 （2）是 |  |
| **12A21** | 儿童父亲现在吸烟吗： （1）不吸烟 （2）每天吸烟 （3）不是每天吸烟 |  |
| **12A22** | 儿童父亲现在是否饮酒？ （1）否 （2）是 |  |
| **12A23** | 您现在是否吸烟: （1）不吸烟 （2）每天吸烟 （3）不是每天吸烟 |  |
| **12A24** | 您现在是否饮酒？ （1）否 （2）是 |  |
| **12A25** | 目前，宝宝平均每周被动吸烟的天数（每天超过15分钟）： （1）0天 （2）1-2天 （3）3-5天 （4）6-7天 （99）不详 |  |
| **12A26** | 体重： （ **Kg**） |  |
| **12A27** | 身长： （ **cm**） |  |
| **12A28** | 头围： （ **cm**） |  |
| **12A29** | 宝宝的出牙数： （ 颗） |  |
| **12A30** | 前囟： （ **cm**× **cm**） |  |
| **12A31** | 体格检查： （1）正常 （2）异常,请注明 ­­­________ |  |
| **12A32** | 检查日期： （ 年 月 日） |  |
| **12A33** | 宝宝目前的主要看护人是？  （1）母亲 （2）父亲（3）（外）祖母（4）（外）祖父（5）保姆或亲戚（全天候）（6）钟点工（7）其他，请注明________________ |  |

# 表12B:儿童睡眠、视力、口腔等情况调查表

| **12B01** | 宝宝精神、行为是否存在异常行为？（1）无 （2）有，请注明 ­­­______________________ |  |
| --- | --- | --- |
| **12B02** | 宝宝的睡眠： （1）正常 （2）异常，(如入睡困难，频繁夜醒，睡眠节律紊乱） |  |
| **12B03** | 睡眠总时间: 小时/日 ，夜间睡眠时间： 小时/日 |  |
| **12B04** | 宝宝入睡方式？ （1）自行入睡 （2）依赖外界帮助，如抱睡、摇晃入睡、吃奶入睡等。 |  |
| **12B05** | 宝宝睡前情绪是否稳定？（1）否 （2）（基本）稳定 （3）不稳定 |  |
| **12B06** | 宝宝是否对手机、iPad、电视等电子产品感兴趣 （1）否 （2）偶尔 （3）经常 |  |
| **12B07** | 宝宝每天注视电子屏幕的时间： 小时/日 |  |
| **12B08** | 宝宝是否测过视力？（1）否**(跳至12B10)** （2）是，请注明多久检查一次 ­­­________ |  |
| **12B09** | 视力是否正常：（1）正常 （2）近视（3）远视（4）斜视（5）弱视 （6）其他，请注明­­­________ |  |
| **12B10** | 是否到医院给宝宝检查过口腔（牙齿）问题？（1）否（2）是，第一次看牙的年龄，请注明________ |  |
| **12B11** | 宝宝是否有含奶嘴入睡的习惯？（1）否（2）偶尔（3）经常 |  |
| **12B12** | 宝宝含奶嘴睡觉持续的时间？（1）无（2）0.5小时以内（3）0.5~1小时（4）1小时以上（5）整晚 |  |
| **12B13** | 家长是否给宝宝喂嚼碎的食物？（1）否（2）偶尔（3）经常 |  |
| **12B14** | 宝宝每日吃零食次数？（1）无（2）1~2次（3）3次及以上 |  |
| **12B15** | 宝宝是否每日定时吃零食？（1）每日定时 （2）每日不定时 |  |
| **12B16** | 宝宝睡前是否吃甜食？（1）否 （2）有时 （3）经常 |  |
| **12B17** | 宝宝是否已经开始刷牙？（1）否 **(跳至表12C)**（2）是，开始刷牙的年龄，请注明________ |  |
| **12B18** | 宝宝每天刷牙次数？（1）0次 （2）早或晚一次 （3）早晚刷牙 |  |
| **12B19** | 宝宝是否使用用含氟儿童牙膏？（1）否（2）偶尔（3）经常 |  |

# 表12C：儿童患病情况

| **12C01** | 您的宝宝自8月龄到12月龄对以下哪些物质有过敏情况**（可多选）**：  （1）无 （2）牛奶 （3）奶酪 （4）全蛋 （5）尘螨 （6）家尘 （7）牛肉 （8）鱼虾蟹贝蛤 （9）狗毛鸡毛 （10）蟑螂  （11）橙（12）各类真菌 （13）黄豆 （14）各类花粉 （15）花生 （16）青胡椒 （17）小麦 （18）鸡肉 （19）蘑菇  （20）其他（请注明） （99）不详 | | | | | | | | | | | | |  |
| --- | --- | --- | --- | --- | --- | --- | --- | --- | --- | --- | --- | --- | --- | --- |
| **12C02** | 您的宝宝8月龄到12月龄有无药物过敏情况**（可多选）**：（1）无**（跳至12C04）**（2）有（请注明） | | | | | | | | | | | | |  |
| **12C03** | 您的宝宝发生药物过敏反应的给药途径：（1）静脉滴注 （2）皮下注射 （3） | | | | | | | | 静脉注射（4）肌肉注射（5）口服给药（99）不详 | | | | |  |
| **12C04** | 您的宝宝8月龄到12月龄患过以下哪些过敏性疾病：  （1）无（2）过敏性皮炎 （3）过敏性鼻炎（4）过敏性哮喘（5）过敏性紫癜（6）过敏性休克 （7）其他 | | | | | | | | | | | | |  |
| **12C05** | 您的宝宝8月龄到12月龄患过以下哪些传染病： （1）无 （2）水痘 （3）腮腺炎 （4）风疹 （5）麻疹 （6）手足口病 （7）肝炎 （8）肺结核（9）其他传染病（请注明） （99）不详 | | | | | | | | | | | | |  |
| **12C06** | 您的宝宝8月龄到12月龄是否患过下列疾病**（可多选）：**  （1）无 （2）上感（感冒）（3）腹泻（4）支气管炎（5）肺炎（6）哮喘（7）佝偻病（8）贫血（9）龋齿（10）其他(请注明) | | | | | | | | | | | | |  |
| **12C07** | 若患过感冒，患感冒次数： 次 | | | | | | | |  | | | | |  |
| **12C08** | 若患过腹泻，患腹泻次数： 次 | | | | | | | |  | | | | |  |
| **12C09** | 您的宝宝8月龄到12月龄是否有因病就医行为： （1）否**（跳至12C14）** （2） 是 | | | | | | | | | | | | |  |
|  | 就诊次数 | 01.  就诊月龄 | | 02.  就诊单位名称 | | 03.  就诊单位类型：（1）药店（2）诊所（3）医院（4）其他 | | 04.  最终诊断 | 05.  主要治疗药物 | | 06.  其他处理 | 07.  疾病转归：（1）痊愈（2）好转（3）无变化 （4）恶化 （5）其他 | |  |
| **12C10** | 第1次 |  | |  | |  | |  |  | |  |  | |  |
| **12C11** | 第2次 |  | |  | |  | |  |  | |  |  | |  |
| **12C12** | 第3次 |  | |  | |  | |  |  | |  |  | |  |
| **12C13** | 第4次 |  | |  | |  | |  |  | |  |  | |  |
| **12C14** | 宝宝是否发生过意外伤害？（1）没有**（跳至表12D）** （2）发生过 | | | | | | | | | | | | |  |
| **12C15** | 如果发生过，是哪种或哪些？**(可多选，并分别将代码填入下表中)**  （1）车祸（2）跌落（3）钝器伤（4）烧烫伤（5）利器伤（6）意外中毒（7）异物窒息（8）触电（9）动物咬伤（10）溺水  （11）其他（请注明） （99）不详 | | | | | | | | | | | | |  |
| 0-12月龄内的宝宝意外伤害基本情况? | | | | | | | | | | | | | | |
|  | 伤害次数 | | 01  发生伤害的种类 | | 02  伤害发生的月龄 | | 03  处理伤害方式  （1）家庭处理（2）送医处理（3）未加处理（99）不详 | | | 04  伤害发生时的看护人  （1）无（2）父母亲（3）（外）祖父母（4）保姆或护工（5）亲属或朋友（6）其他（99）不详 | | | 05  现在宝宝恢复情况？  （1）恢复情况尚不乐观（2）有明显好转，但尚未完全恢复（3）已经完全恢复（99）不详 |  |
| **12C16** | 第1次 | |  | |  | |  | | |  | | |  |  |
| **12C17** | 第2次 | |  | |  | |  | | |  | | |  |  |
| **12C18** | 第3次 | |  | |  | |  | | |  | | |  |  |
| **12C19** | 第4次 | |  | |  | |  | | |  | | |  |  |
| **12C20** | 第5次 | |  | |  | |  | | |  | | |  |  |
| **12C21** | 第6次 | |  | |  | |  | | |  | | |  |  |
| **12C22** | 第7次 | |  | |  | |  | | |  | | |  |  |
| **12C23** | 第8次 | |  | |  | |  | | |  | | |  |  |
| **12C24** | 第9次 | |  | |  | |  | | |  | | |  |  |
| **12C25** | 第10次 | |  | |  | |  | | |  | | |  |  |

# 表12D：宝宝添加辅食情况表

| **12D01** | 目前宝宝食用的主食是什么？ | |  |  |
| --- | --- | --- | --- | --- |
| **12D02** | 目前宝宝是否已经添加辅食：（1）否 （2）是，添加辅食的月龄 月 | |  |  |
| **12D03** | 昨天（包括白天和夜里），宝宝吃过辅食的种类： 种。 | |  |  |
| **12D04** | 昨天（包括白天和夜里），宝宝吃过 餐次辅食 (填次数，辅食指正餐和零食） | |  |  |
|  | **食物品种** | **食用频率** | **12D05**  **食用频率** | **12D06**  **开始添加月龄** |
| **01** | 谷薯类 | （1）未添加 （2）每天吃1次 （3）每天吃≥2次 |  |  |
| **02** | 奶类及制品 | （1）未添加 （2）每月吃1~2次 （3）每周吃1~2次 （4）每周吃3~4次 （5）每周吃≥5次 |  |  |
| **03** | 蛋类 | （1）未添加 （2）每月吃1~2次 （3）每周吃1~2次 （4）每周吃3~4次 （5）每周吃≥5次 |  |  |
| **04** | 禽畜肉类 | （1）未添加 （2）每月吃1~2次 （3）每周吃1~2次 （4）每周吃3~4次 （5）每周吃≥5次 |  |  |
| **05** | 鱼类 | （1）未添加 （2）每月吃1~2次 （3）每周吃1~2次 （4）每周吃3~4次 （5）每周吃≥5次 |  |  |
| **06** | 豆类及制品 | （1）未添加 （2）每月吃1~2次 （3）每周吃1~2次 （4）每周吃3~4次 （5）每周吃≥5次 |  |  |
| **07** | 新鲜水果 | （1）未添加 （2）每月吃1~2次 （3）每周吃1~2次 （4）每周吃3~4次 （5）每周吃≥5次 |  |  |
| **08** | 新鲜蔬菜 | （1）未添加 （2）每月吃1~2次 （3）每周吃1~2次 （4）每周吃3~4次 （5）每周吃≥5次 |  |  |
| **09** | 动物内脏 | （1）未添加 （2）每月吃1~2次 （3）每周吃1~2次 （4）每周吃3~4次 （5）每周吃≥5次 |  |  |
| **10** | 坚果 | （1）未添加 （2）每月吃1~2次 （3）每周吃1~2次 （4）每周吃3~4次 （5）每周吃≥5次 |  |  |
| **11** | 食用油 | (1)未添加 （2）每月吃1~2次 （3）每周吃1~2次 （4）每周吃3~4次 （5）每周吃≥5次 |  |  |

# 表12E： 儿童环境危险因素调查表

| **家庭内部环境调查** | | |
| --- | --- | --- |
| 12E01 | 是否存在以下室内污染现象？**（可多选）**  （1）无 （2）每天清晨起床时，感到[憋闷](http://zhidao.baidu.com/search?word=憋闷&fr=qb_search_exp&ie=utf8)、恶心、甚至头晕目眩。  （3）家里小孩常咳嗽、打喷嚏、免疫力下降，新装修的房子宝宝不愿回家。  （4）家人共有一种疾病，而且离开这个环境后，症状就有明显变化和好转。  （5）家人常有皮肤过敏等毛病，而且是群发性的。 （6）其他 |  |
| 12E02 | 是否存在以下家庭环境污染的因素？**（可多选）**  （1）无 （2）自怀孕前半年以来，进行过房屋装修或添置新家具 （3）采暖、烹饪产生油烟 （4）（基本）不进行房屋通风换气  （5）家中宠物（基本）不进行定期健康检查 （6）其他，（请注明） |  |
| 12E03 | 您的宝宝在室内是否受到噪音的困扰？ （1）否 （2）是 （99）不详 |  |
| 12E04 | 您的宝宝生活环境中是否存在辐射？ （1）否 （2）是 （99）不详 |  |
| 12E05 | 宝宝生活范围内，是否有人患传染病？ （1）否 （2）是 （99）不详 |  |
| 12E06 | 是何种传染病？**（可多选）** （1）流感 （2）流行性腮腺炎 （3）百日咳 （4）水痘 （5）麻疹 （6）乙型脑炎  （7）菌痢 （8）流脑 （9）肝炎（10）结核病 （11）其他，请注明 （99）不详 |  |

# 表12F：儿童计划免疫表

| 12F01 | 目前您的宝宝是否接种了以下哪些疫苗：（1）否（跳至表12G）（2）乙肝疫苗（3）卡介苗 （4）脊灰疫苗（5）百白破疫苗 （6）白破疫苗 （7）麻风疫苗（8）麻腮风疫苗（9）麻腮疫苗（10）麻疹疫苗（11）A群流脑疫苗（12）A+C群流脑疫苗（13）乙脑（减毒）活疫苗（14）乙脑灭活疫苗（15）甲肝减毒活疫苗（16）甲肝灭活疫苗（17）水痘疫苗（18）B型流感嗜血杆菌疫苗（19）7价肺炎球菌疫苗（20）口服轮状病毒疫苗（14）其他疫苗 | | | | |  |
| --- | --- | --- | --- | --- | --- | --- |
| 12F02 | 宝宝是否在规定时间内接种相应疫苗？（1）否（2）是 | | | | |  |
| 12F03 | 宝宝未按期接种疫苗的原因是？ （1）宝宝身体不适，不能接种（2）家长忘记接种日期（3）家长认为可不必按期接种 | | | | |  |
| 12F04 | 宝宝是否有接种禁忌： （1）无 （2）有 （99）不详 | | | | |  |
| 12F05 | 宝宝是否有传染病史: （1）无 （2）有 （99）不详 | | | | |  |
| 12F06 | 有无疫苗异常反应史： （1）无**（跳至表12G）** （2）有（共发生过 次） （99）不详 | | | | | |
| 编号 | 异常次数 | 疫苗名称 | 接种月龄 | 异常反应类型 | 处理措施 | |
| 12F07 | 第1次异常 |  |  |  |  | |
| 12F08 | 第2次异常 |  |  |  |  | |
| 12F09 | 第3次异常 |  |  |  |  | |

# 表12G：0~1岁宝宝家庭养育环境量表

| **因素** | **条目** | （1） 是（2）否 | **因素** | **条目** | （1）是（2）否 |
| --- | --- | --- | --- | --- | --- |
| 感知**/**  认知 | G01引导宝宝翻身爬行，探索周围环境 |  | 忽视/限制 | G20凭自己心情对宝宝忽冷忽热 |  |
|  | G02给宝宝做被动体操、主动体操 |  |  | G21为了家庭或宝宝的事情而争吵、闹别扭 |  |
|  | G03看护人了解各种玩具的玩法及功用 |  |  | G22不太喜欢宝宝，不远和宝宝在一起 |  |
|  | G04带宝宝户外活动 |  |  | G23感到带宝宝很累、很烦 |  |
|  | G05宝宝房间光线明亮，布置得丰富多彩 |  |  | G24把宝宝包裹捆扎得很紧，四肢手足难以自主活动 |  |
|  | G06让宝宝参加早期潜能开发活动 |  |  | G25终日在外面忙碌，很少有时间见到宝宝 |  |
|  | G07有意识训练宝宝发音 |  |  | G26总担心出事，把宝宝活动限制的很死 |  |
|  | G08训练宝宝手的操控动作 |  | 人际互动/喂养 | G27给宝宝接触其他儿童一起游戏的机会 |  |
|  | G09家里拥有适合宝宝年龄的玩具 |  |  | G28给宝宝及时、合理地添加各种辅食 |  |
|  | G10与宝宝一起进行玩具游戏 |  |  | G29注重学习了解宝宝发育和保健知识 |  |
|  | G11拥有适合宝宝年龄的各种画书图片 |  |  | G30宝宝由亲生父母亲自带养看护 |  |
|  | G12宝宝活动的房间保持清洁、整齐 |  |  | G31有意识播放或哼唱音乐给宝宝听 |  |
|  | G13有意识让宝宝接触认识各种物品 |  |  | G32关注宝宝维生素/微量元素的营养问题 |  |
| 情感  温暖 | G14与宝宝有肢体接触和目光交流 |  |  | | |
|  | G15有意识的和宝宝说话 |  |  |  |  |
|  | G16亲吻、拥抱、爱抚宝宝 |  |  |  |  |
|  | G17当宝宝出现与平常不一样的表现便很着急 |  |  |  |  |
|  | G18对宝宝需求及时作出回应 |  |  |  |  |
|  | G19对导致宝宝精神状态不佳的原因十分清楚 |  |  |  |  |

# 表12H：0~2岁儿童父母育儿评估量表

| **因素** | **条目** | （1）是（2）否 | **因素** | **条目** | （1）是（2）否 |
| --- | --- | --- | --- | --- | --- |
| 育儿观念 | H01家长是否知道什么样的宝宝是健康的 |  | 语言刺激 | H21家长是否有意识地播放或哼唱音乐给宝宝听 |  |
|  | H02家长是否能够发现自己宝宝的特点 |  |  | H22家长对宝宝的发生用语音进行回应 |  |
|  | H03家长是否能够在日常生活中为宝宝提供学习的机会 |  |  | H23告诉宝宝物体名称或来访客人的称谓 |  |
|  | H04家长是否认为父母应该担负起养育宝宝的主要责任 |  |  | H24家长对宝宝说话时，是否能使用正确的语句，发音清晰 |  |
|  | H05家长是否能够认识到自己在育儿过程中有哪些不足，并自觉改进 |  | 外出活动 | H25家长是否经常让宝宝与亲友相处，培养人际交往的能力 |  |
| 关注 | H06访谈期间，家长是否注视自己的宝宝并有话语交流、爱抚动作 |  |  | H26家长是否为宝宝提供与其他小朋友接触、玩耍的机会 |  |
|  | H07当家长做家务劳动时是否和宝宝进行交谈 |  |  | H27家长是否带宝宝去郊游或旅行 |  |
|  | H08家长是否关注、欣赏并鼓励宝宝的进步和成长 |  |  | H28家长是否经常带宝宝进行户外活动 |  |
|  | H09宝宝哭闹时，家长是否及时查找原因，并适当处理 |  | 喂养 | H29家长是否在宝宝6月龄时及时添加辅食 |  |
|  | H10家长是否了解宝宝生病的表现 |  |  | H30家长是否知道辅食添加的顺序 |  |
| 亲子关系 | H11访谈期间，家长自然地赞扬宝宝至少2次 |  |  | H31家长给宝宝添加的辅食是否营养全面 |  |
|  | H12宝宝需要临时看护时，是否由熟悉的人来看护 |  |  | H32家长是否采用积极的喂养方式 |  |
|  | H13家长每天与宝宝相处时间不少于2小时 |  | 安全 | H33家长是否注意居住环境的环保 |  |
|  | H14全家经常在一起吃饭并有良好的气氛 |  |  | H34室内是否有安全防护措施 |  |
|  | H15父亲每天也照顾宝宝 |  |  | H35药物、清洁剂、化妆品、锐器、电插座等是否在宝宝够不到的地方 |  |
| 学习材料 | H16宝宝是否拥有能帮助学习颜色、形状、大小的玩具 |  |  | H36家长是否知道紧急救护儿童的知识 |  |
|  | H17宝宝是否拥有锻炼大动作及精细动作的玩具 |  |  | | |
|  | H18家中是否有宝宝自己的图画书 |  |  |  |  |
|  | H19室内是否有符合儿童特点的装饰、图画和用具 |  |  |  |  |
|  | H20宝宝有专门存放玩具或物品的地方 |  |  |  |  |
