# Supplementary material for: Effect of feeding patterns on growth and nutritional status of children aged 0-24 months: A Chinese cohort study
Source: PLoS One. 2019 Nov 19;14(11):e0224968. doi: 10.1371/journal.pone.0224968 (PMC6863544; doi:10.1371/journal.pone.0224968)
Supplement: S2 Text — (ZIP) [file pone.0224968.s002.zip › 18╘┬┴Σ.doc]

**编号： □□□□□□**

**2016年开福区社区母婴健康信息采集（18月龄）**

长沙市开福区	社区卫生服务中心

居住地址：	区（县）	街道（路）	社区

母亲姓名：	联系电话：

父亲姓名：	联系电话：

子女姓名：	子女性别：

子女出生日期：

信息收集时间：	年	月	日	信息收集方式：	语音资料编号：

信息收集人：	信息核查人：

目录

表18A：18月龄儿童随访记录表	3

表18B：儿童睡眠、视力、口腔、行为等情况调查表	5

表18C：儿童患病情况	6

表18D：宝宝添加辅食及喂养行为情况	8

表18E：儿童环境危险因素调查表	9

表18F：儿童计划免疫表	9

表18G：1~3岁宝宝家庭养育环境量表	11

表18H：0~2岁儿童父母育儿评估量表	12

表18I：0~3岁儿童心智发育进程记录	13

# 表18A：18月龄儿童随访记录表

| **18A01** | 检查日期： （ 年 月 日） |
| --- | --- |
| **18A02** | 实足月龄：（ 月龄 天） |
| **18A03** | 体重：（ **Kg**） |
| **18A04** | 身长：（ **cm**） |
| **18A05** | 头围：（ **cm**） |
| **18A06** | 宝宝前囟是否闭合？（1）否 （2）是**（跳至18A08）** |
| **18A07** | 前囟： cm× cm |
| **18A08** | 宝宝前囟闭合时间： 月龄 |
| **18A09** | 宝宝的出牙数：（ 颗） |
| **18A10** | 体格检查： （1）正常**（跳至18A12）** （2）异常 |
| **18A11** | 体格检查异常为： |
| **18A12** | 目前家里有 个孩子 |
| **18A13** | 宝宝是第 个孩子 |
| **18A14** | 宝宝12月龄到18月龄的主要看护人是**（可多选）**？  （1）母亲 （2）父亲（3）（外）祖母（4）（外）祖父（5）保姆（钟点工）（6）亲戚或朋友（7）其他，请注明________________ |
| **18A15** | 父亲平均每天照顾宝宝的时间：（1）少于1小时 （2）1~3小时 （3）3~5小时 （4）5~8小时 （5）8~12小时 （6）12小时以上 |
| **18A16** | 母亲平均每天照顾宝宝的时间：（1）少于1小时 （2）1~3小时 （3）3~5小时 （4）5~8小时 （5）8~12小时 （6）12小时以上 |
| **18A17** | 喂养方式： （1）纯母乳喂养 （2）人工喂养**（跳至18A19）** （3）混合喂养 |
| **18A18** | 母乳次数： （ 次/日） |
| **18A19** | 宝宝是否已断母乳？（1）否**（跳至18A21）** （2）是，断母乳的月龄是 月 天 |
| **18A20** | 您给宝宝断奶的原因是什么？**（可多选，选任一项均跳至18A22）**（1）工作需求 （2）生病 （3）无母乳（4）觉得母乳喂养麻烦 （5）担心影响身材或形象 （6）认为配方奶粉更有营养（7）宝宝生病（8）宝宝拒绝吸吮（9）觉得宝宝已到断乳年龄（10）哺乳时宝宝会咬乳头（11）其他，请注明： |
| **18A21** | 打算母乳喂养多久？ 月 |
| **18A22** | 宝宝12月龄到18月龄是否使用带奶嘴的奶瓶喝水、奶类或果汁等？ （1）否 （2）偶尔 （3）经常 |
| **18A23** | 宝宝开始使用带奶嘴的奶瓶喝水、奶类或果汁等的时间： 月龄 |
| **18A24** | 宝宝12月龄到18月龄期间，是否有含奶嘴（安抚奶嘴除外）入睡的习惯？ （1）否**（跳至18A27）** （2）偶尔 （3）经常 |
| **18A25** | 宝宝何时开始含奶嘴（安抚奶嘴除外）入睡？ 月龄 |
| **18A26** | 宝宝含奶嘴睡觉持续的时间？（1）0.5小时以内 （2）0.5~1小时 （3）1小时以上 （4）整晚 |
| **18A27** | 宝宝12月龄到18月龄是否使用安抚奶嘴？ （1）否**（跳至18A30）** （2）是 |
| **18A28** | 宝宝开始使用安抚奶嘴的时间： 月龄 |
| **18A29** | 宝宝通常在什么情况下使用安抚奶嘴**（可多选）**？（1）宝宝哭闹时 （2）宝宝睡觉时 （3）宝宝咬手指时 （4）帮助宝宝锻炼口腔吸吮和吞咽能力时  （5）其他，（请注明）： |
| **18A30** | 目前您是否给宝宝添加配方奶粉或鲜奶？ （1）未添加**（跳至18A32）** （2）已添加，首次添加时间为： 月龄 天 |
| **18A31** | 配方奶粉或鲜奶情况： （ 次/天），每次 **ml** |
| **18A32** | 宝宝12月龄到18月龄是否做过微量元素的检测？（1）否**（跳至18A33）** （2）是 |
| **18A32-1** | 检测月龄： ，微量元素检测结果是否正常？（1）正常 （2）缺钙 （3）缺铁 （4）缺锌 （5）缺镁 （6）铜超标 （7）铅超标 （8）其他 |
| **18A32-2** | 检测月龄： ，微量元素检测结果是否正常？（1）正常 （2）缺钙 （3）缺铁 （4）缺锌 （5）缺镁 （6）铜超标 （7）铅超标 （8）其他 |
| **18A32-3** | 检测月龄： ，微量元素检测结果是否正常？（1）正常 （2）缺钙 （3）缺铁 （4）缺锌 （5）缺镁 （6）铜超标 （7）铅超标 （8）其他 |
| **18A33** | 宝宝12月龄到18月龄补充钙剂的频率：（1）基本不吃 （2）每月吃1~2次 （3）每周吃1~2次 （4）每周吃3~4次 （5）每周吃≥5次 |
| **18A34** | 宝宝12月龄到18月龄服用维生素D的频率：（1）基本不吃 （2）每月吃1~2次 （3）每周吃1~2次 （4）每周吃3~4次 （5）每周吃≥5次 |
| **18A35** | 宝宝12月龄到18月龄是否补充其他营养补充剂? （1）否**（跳至18A37）**（2）是，请注明： |
| **18A36** | 宝宝开始补充其他营养补充剂的时间： 月龄 |
| **18A37** | 宝宝12月龄到18月龄平均每周户外活动的天数：（1）0天**（跳至18A39）** （2）1~2天 （3）3~5天 （4）6~7天 （99）不详 |
| **18A38** | 宝宝平均每天户外活动的时间： 小时/日 |
| **18A39** | 在宝宝12月龄到18月龄间，宝宝父母是否参加过育儿方面的培训？（1）否**（跳至18A41）** （2）是 |
| **18A40** | 宝宝父母参加育儿培训的原因是**（可多选）**： （1）学习科学的育儿知识和方法 （2）可以解决育儿方面的困惑 （3）有利于宝宝的教育和成长  （4）有利于更好地做好父母的角色 （5）可以减少育儿过程中的心理焦虑 （6）其他，请注明 |
| **18A41** | 宝宝是否参加早教班？（1）否**（跳至18A46）** （2）是 |
| **18A42** | 宝宝开始参加早教班的时间： 月 |
| **18A43** | 宝宝参加早教班的原因是**（可多选）：**（1）有利于提高宝宝的认知、语言表达、运动、社会交往等能力 （2）有利于开发宝宝的潜能（3）早教的课程内容可以作为教养的指导 （4）可以为宝宝上幼儿园打基础 （5）没有时间照看宝宝 （6）其他，请注明 |
| **18A44** | 宝宝12月龄到18月龄间，参加早教班的次数： 次 |
| **18A45** | 您觉得宝宝参加早教班有效果吗？（1）没有 （2）有，请注明 |
| **18A46** | 宝宝12月龄到18月龄间，宝宝父亲是否吸烟？ （1）否**（跳至18A48）** （2）是 |
| **18A47** | 宝宝父亲吸烟量： 支/日**（1包烟=20支烟）** |
| **18A48** | 宝宝12月龄到18月龄间，宝宝父亲是否饮酒？ （1）否**（跳至18A51）** （2）是 |
| **18A49** | 宝宝父亲饮酒频率： 次/周 |
| **18A50** | 宝宝父亲的饮酒量： **g**/次  （各类酒的饮酒量均折算成高度白酒计算，1瓶啤酒或1斤米酒≈1两高度白酒，1斤葡萄酒或黄酒≈2.5两高度白酒，2两低度白酒 ≈1.5两高度白酒，1两谷酒≈1两高度白酒，1两=50g，高度白酒或谷酒45~60%（v/v），低度白酒35~44%（v/v），葡萄酒或黄酒12~18%（v/v），啤酒或米酒4~6%（v/v）） |
| **18A51** | 宝宝12月龄到18月龄间，您是否吸烟？ （1）否**（跳至18A53）** （2）是 |
| **18A52** | 您的吸烟量： 支/日**（1包烟=20支烟）** |
| **18A53** | 宝宝12月龄到18月龄间，您是否饮酒？ （1）否**（跳至18A56）** （2）是 |
| **18A54** | 您的饮酒频率： 次/周 |
| **18A55** | 您的饮酒量： **g**/次  **（**各类酒的饮酒量均折算成高度白酒计算，1瓶啤酒或1斤米酒≈1两高度白酒，1斤葡萄酒或黄酒≈2.5两高度白酒，2两低度白酒 ≈1.5两高度白酒，1两谷酒≈1两高度白酒，1两=50g，高度白酒或谷酒45~60%（v/v），低度白酒35~44%（v/v）葡萄酒或黄酒12~18%（v/v），啤酒或米酒4~6%（v/v）） |
| **18A56** | 目前，宝宝平均每周被动吸烟的天数（每天超过15分钟）：（1）0天**（跳至18B）** （2）1~2天 （3）3~5天 （4）6~7天 （99）不详 |
| **18A57** | 宝宝被动吸烟的主要来源是**（可多选）:** （1）家里 （2）小区 （3）公园 （4）游乐园 （5）其他，请注明 ： （99）不详 |

# 表18B：儿童睡眠、视力、口腔、行为等情况调查表

| **18B01** | 宝宝的睡眠：（1）正常 （2）异常，(如入睡困难，频繁夜醒，睡眠节律紊乱) |
| --- | --- |
| **18B02** | 睡眠总时间: 小时/日 ，夜间睡眠时间： 小时/日 |
| **18B03** | 宝宝入睡方式？ （1）自行入睡 （2）依赖外界帮助，如抱睡、摇晃入睡、吃奶入睡等。 |
| **18B04** | 宝宝晚上与谁一起睡**（可多选）**？（1）母亲（2）父亲（3）（外）祖母（4）（外）祖父（5）保姆（钟点工）（6）亲戚或朋友（7）其他，请注明 |
| **18B05** | 宝宝睡前情绪是否稳定？（1）否 （2）是 |
| **18B06** | 宝宝是否对手机、iPad、电视等电子产品感兴趣？ （1）否**（跳至18B10）** （2）偶尔 （3）经常 |
| **18B07** | 宝宝通常会看哪些电子产品**（可多选）**？（1）手机 （2）电视 （3）iPad （4）游戏机 （5）计算机 （6）其他，请注明________________ |
| **18B08** | 宝宝通常会在什么情况下看手机、iPad、电视等电子产品**（可多选）?** （1）宝宝学习巧虎等早教视频 （2）宝宝哭闹时，用于安抚 （3）看护人为了省心，主动让宝宝使用 （4）大人在使用这类电子产品时，宝宝在旁边看 （5）其他，请注明________________________ |
| **18B09** | 宝宝每天注视电子屏幕的时间： 小时/日 |
| **18B10** | 宝宝12月龄到18月龄是否测过视力？（1）否**（跳至18B11）** （2）是 |
| **18B10-1** | 检测月龄： ，视力是否正常：（1）正常 （2）近视（3）远视（4）斜视（5）弱视 （6）散光 （7）其他，请注明________ |
| **18B10-2** | 检测月龄： ，视力是否正常：（1）正常 （2）近视（3）远视（4）斜视（5）弱视 （6）散光 （7）其他，请注明________ |
| **18B10-3** | 检测月龄： ，视力是否正常：（1）正常 （2）近视（3）远视（4）斜视（5）弱视 （6）散光 （7）其他，请注明________ |
| **18B11** | 宝宝12月龄到18月龄是否检查过口腔（牙齿）问题？（1）否**（跳至18B12）** （2）是 |
| **18B11-1** | 检测月龄： 口腔是否正常：（1）正常 （2）龋齿 （3）其他，请注明________ |
| **18B11-2** | 检测月龄： 口腔是否正常：（1）正常 （2）龋齿 （3）其他，请注明________ |
| **18B11-3** | 检测月龄： 口腔是否正常：（1）正常 （2）龋齿 （3）其他，请注明________ |
| **18B12** | 是否给宝宝喂嚼碎的食物？（1）否（2）偶尔（3）经常 |
| **18B13** | 宝宝每日吃零食次数？（1）0次**（跳至18B16）** （2）1~2次（3）3次及以上 **（****零食：指非正餐时间食用的各种少量的食物和 /或饮料（不包括水））** |
| **18B14** | 宝宝是否每日定时吃零食？（1）定时 （2）不定时 |
| **18B15** | 宝宝吃零食的种类是**（可多选）**：（1）膨化食品 （2）糖果类 （3）谷类 （4）饮料类（5）新鲜水果蔬菜 （6）坚果类 （7）肉蛋类 （8）海产品  （9）油炸类 （10）薯类 （11）其他，请注明_______ |
| **18B16** | 宝宝睡前是否吃含糖甜食？（1）否 （2）有时 （3）经常 |
| **18B17** | 宝宝是否已经开始刷牙？（1）否**（跳至表18B21）** （2）是 |
| **18B18** | 宝宝开始刷牙的时间：_____ 月龄 |
| **18B19** | 宝宝每天刷牙次数？（1）早或晚一次 （2）早晚刷牙 （3）不固定 |
| **18B20** | 宝宝是否使用含氟儿童牙膏？（1）否 （2）偶尔 （3）经常 |
| **18B21** | 宝宝12月龄到18月龄是否吸吮手指？（1）否**（跳至18C）** （2）有时 （3）经常 |
| **18B22** | 宝宝开始吸吮手指的时间：_____月龄 |
| **18B23** | 宝宝吸吮手指的习惯已持续多久？ 月 |
| **18B24** | 宝宝通常会在什么情况下吸吮手指？（1）宝宝一个人单独玩时 （2）宝宝饥饿时 （3）宝宝睡觉时 （4）习惯性动作 （5）其他，请注明 |
| **18B25** | 宝宝吸吮手指时，您是否会阻止？（1）否 （2）偶尔会 （3）每次都会 |

# 表18C：儿童患病情况

| **18C01** | 宝宝12月龄到18月龄对以下哪些物质有过敏情况**（可多选）**：  （1）无 （2）牛奶 （3）奶酪 （4）全蛋 （5）尘螨 （6）家尘 （7）牛肉 （8）鱼虾蟹贝蛤 （9）狗毛鸡毛 （10）蟑螂  （11）橙（18）各类真菌 （13）黄豆 （14）各类花粉 （15）花生 （16）青胡椒 （17）小麦 （18）鸡肉 （19）蘑菇  （20）其他（请注明） （99）不详 | | | | | | | | | | | | | | | | |
| --- | --- | --- | --- | --- | --- | --- | --- | --- | --- | --- | --- | --- | --- | --- | --- | --- | --- |
| **18C02** | 宝宝12月龄到18月龄有无药物过敏情况：（1）无**（跳至18C03）**（2）有 | | | | | | | | | | | | | | | | |
|  | 发生次数 | | | 发生时间 | | | 药物名称 | | | 给药途径  （1）静脉滴注 （2）皮下注射 （3）静脉注射（4）肌肉注射（5）口服给药（99）不详 | | | | | | | |
| **18C02-1** | 第1次 | | |  | | |  | | |  | | | | | | | |
| **18C02-2** | 第2次 | | |  | | |  | | |  | | | | | | | |
| **18C02-3** | 第3次 | | |  | | |  | | |  | | | | | | | |
| **18C03** | 宝宝12月龄到18月龄患过以下哪些过敏性疾病**（可多选）**：  （1）无 （2）湿疹 （3）过敏性皮炎 （4）过敏性鼻炎 （5）过敏性哮喘 （6）过敏性紫癜 （7）过敏性休克 （8）其他(请注明) | | | | | | | | | | | | | | | | |
| **18C04** | 宝宝12月龄到18月龄患过以下哪些传染病**（可多选）**： （1）无 （2）水痘 （3）腮腺炎 （4）风疹 （5）麻疹 （6）手足口病 （7）病毒性肝炎  （8）肺结核 （9）疱疹性咽峡炎 （10）其他传染病（请注明） （99）不详 | | | | | | | | | | | | | | | | |
| **18C05** | 宝宝12月龄到18月龄是否患过下列疾病**（可多选）：**  （1）无 （2）急性上呼吸道感染（感冒） （3）腹泻（4）支气管炎（5）肺炎（6）哮喘（7）佝偻病（8）贫血（9）龋齿（10）其他(请注明) | | | | | | | | | | | | | | | | |
| **18C06** | 若患过感冒，患感冒次数： 次 | | | | | | | | | |  | | | | | | |
| **18C07** | 若患过腹泻，患腹泻次数： 次 | | | | | | | | | |  | | | | | | |
| **18C08** | 宝宝12月龄到18月龄是否有因病就医行为： （1）否**（跳至18C09）** （2） 是 | | | | | | | | | | | | | | | | |
|  | 就诊次数 | 就诊月龄 | | | 就诊单位名称 | | | 就诊单位类型  （1）药店（2）诊所（3）医院  （4）社区卫生服务中心（5）其他 | | | | | 最终诊断 | 主要治疗药物 | 其他处理 | | 疾病转归  （1）痊愈（2）好转（3）无变化 （4）恶化 （5）其他 |
| **18C08-1** | 第1次 |  | | |  | | |  | | | | |  |  |  | |  |
| **18C08-2** | 第2次 |  | | |  | | |  | | | | |  |  |  | |  |
| **18C08-3** | 第3次 |  | | |  | | |  | | | | |  |  |  | |  |
| **18C08-4** | 第4次 |  | | |  | | |  | | | | |  |  |  | |  |
| **18C09** | 宝宝12月龄到18月龄是否发生过意外伤害**（凡具有下列两种情况之一者一种情况者可作为伤害：①到医疗单位诊断为某一类损伤；②因伤休息（休工或休学）一日以上）**？ （1）否**（跳至表18D）** （2）是 | | | | | | | | | | | | | | | | |
| **18C10** | 如果发生过，是哪种或哪些？**（可多选，并分别将代码按发生时间填入伤害类别中）**  （1）车祸 （2）跌落 （3）钝器伤 （4）烧烫伤 （5）利器伤 （6）意外中毒 （7）异物窒息 （8）触电（9）动物咬伤（10）溺水  （11）其他（请注明） （99）不详 | | | | | | | | | | | | | | | | |
|  | 发生  次数 | | 伤害类别 | | | 发生月龄 | | | 处理伤害方式  （1）家庭处理（2）送医处理  （3）未加处理（99）不详 | | | 伤害发生时的看护人  （1）无（2）父母亲（3）（外）祖父母（4）保姆或护工（5）亲属或朋友（6）其他（99）不详 | | | | 现在宝宝恢复情况？  （1）尚不乐观（2）明显好转  （3）完全恢复（99）不详 | |
| **18C10-1** | 第1次 | |  | | |  | | |  | | |  | | | |  | |
| **18C10-2** | 第2次 | |  | | |  | | |  | | |  | | | |  | |
| **18C10-3** | 第3次 | |  | | |  | | |  | | |  | | | |  | |
| **18C10-4** | 第4次 | |  | | |  | | |  | | |  | | | |  | |
| **18C10-5** | 第5次 | |  | | |  | | |  | | |  | | | |  | |
| **18C10-6** | 第6次 | |  | | |  | | |  | | |  | | | |  | |
| **18C10-7** | 第7次 | |  | | |  | | |  | | |  | | | |  | |
| **18C10-8** | 第8次 | |  | | |  | | |  | | |  | | | |  | |
| **18C10-9** | 第9次 | |  | | |  | | |  | | |  | | | |  | |

# 表18D：宝宝添加辅食及喂养行为情况

| **18D01** | 宝宝12月龄到18月龄食用的主食是什么？ | | | |
| --- | --- | --- | --- | --- |
| **18D02** | 昨天（包括白天和夜里），宝宝吃过辅食的种类： 种。 | | | |
| **18D03** | 昨天（包括白天和夜里），宝宝吃过 餐次辅食 (填次数，辅食指正餐和零食） | | | |
|  | **食物品种** | **食用频率** | **频率** | **开始添加月龄** |
| **18D03-1** | 谷薯类 | （1）未添加 （2）每天吃1次 （3）每天吃2次 （4）每天吃≥3次 |  |  |
| **18D03-2** | 奶类及制品 | （1）未添加 （2）每月吃1~2次 （3）每周吃1~2次 （4）每周吃3~4次 （5）每周吃≥5次 |  |  |
| **18D03-3** | 蛋类 | （1）未添加 （2）每月吃1~2次 （3）每周吃1~2次 （4）每周吃3~4次 （5）每周吃≥5次 |  |  |
| **18D03-4** | 禽畜肉类 | （1）未添加 （2）每月吃1~2次 （3）每周吃1~2次 （4）每周吃3~4次 （5）每周吃≥5次 |  |  |
| **18D03-5** | 鱼类 | （1）未添加 （2）每月吃1~2次 （3）每周吃1~2次 （4）每周吃3~4次 （5）每周吃≥5次 |  |  |
| **18D03-6** | 豆类及制品 | （1）未添加 （2）每月吃1~2次 （3）每周吃1~2次 （4）每周吃3~4次 （5）每周吃≥5次 |  |  |
| **18D03-7** | 新鲜水果 | （1）未添加 （2）每月吃1~2次 （3）每周吃1~2次 （4）每周吃3~4次 （5）每周吃≥5次 |  |  |
| **18D03-8** | 新鲜蔬菜 | （1）未添加 （2）每月吃1~2次 （3）每周吃1~2次 （4）每周吃3~4次 （5）每周吃≥5次 |  |  |
| **18D03-9** | 动物内脏 | （1）未添加 （2）每月吃1~2次 （3）每周吃1~2次 （4）每周吃3~4次 （5）每周吃≥5次 |  |  |
| **18D03-10** | 坚果 | （1）未添加 （2）每月吃1~2次 （3）每周吃1~2次 （4）每周吃3~4次 （5）每周吃≥5次 |  |  |
| **18D03-11** | 食用油 | （1）未添加 （2）每月吃1~2次 （3）每周吃1~2次 （4）每周吃3~4次 （5）每周吃≥5次 |  |  |
| **18D04** | 平时单独给宝宝做饭吗？ （1）不会 （2）偶尔 （3）有时 （4）经常 （5）总是 | | | |
| **18D05** | 同一种食物您会用不同的做法来促进宝宝食欲吗？ （1）没有 （2）偶尔 （3）有时 （4）经常 （5）总是 | | | |
| **18D06** | 您会将食物捣成糊状或切成小块后再给宝宝喂食吗？ （1）不会 （2）偶尔 （3）有时 （4）经常 （5）总是 | | | |
| **18D07** | 您会根据宝宝饥饿饱足信号进行喂食吗？ （1）不会 （2）偶尔 （3）有时 （4）经常 （5）总是 | | | |
| **18D08** | 宝宝进食前，您会给宝宝洗手吗? （1）不会 （2）偶尔 （3）有时 （4）经常 （5）总是 | | | |
| **18D09** | 宝宝进食时，您和宝宝有语言性鼓励和感情交流吗？ （1）没有 （2）偶尔 （3）有时 （4）经常 （5）总是 | | | |
| **18D10** | 您会训练宝宝自己拿勺子或者筷子吃饭吗？ （1）不会 （2）偶尔 （3）有时 （4）经常 （5）总是 | | | |
| **18D11** | 当宝宝拒绝某种食物时，您会： （1）不再喂食该食物 （2）暂时放弃，下次再尝试 （3）温柔地鼓励宝宝尝试 （4）强迫或追着宝宝喂食 | | | |
| **18D12** | 宝宝进食时，是否会出现以下行为**（可多选，选择选项（2）时请回答18D13，其余跳至18D14）**： （1）否 （2）对食物不感兴趣 （3）强烈偏爱某些食物 （4）拒绝某些食物 （5）进餐分心 （6）进餐地点不固定 （7）进餐时间超过30分钟 （8）其他，请注明 | | | |
| **18D13** | 宝宝对食物不感兴趣的原因是？ （1）宝宝过多吃零食 （2）宝宝活动量不足 （3）宝宝消化不良 （4）宝宝缺锌 （5）其他，请注明 | | | |
| **18D14** | 宝宝在进食时有无电视、玩具、手机等的干扰？ （1）无 （2）偶尔 （3）有时 （4）经常 （5）总是 | | | |

# 表18E：儿童环境危险因素调查表

| **家庭内部环境调查** | |
| --- | --- |
| **18E01** | 是否存在以下室内污染现象？**（可多选）**  （1）无 （2）每天清晨起床时，感到[憋闷](http://zhidao.baidu.com/search?word=憋闷&fr=qb_search_exp&ie=utf8)、恶心、甚至头晕目眩。  （3）家里小孩常咳嗽、打喷嚏、免疫力下降，新装修的房子宝宝不愿回家。  （4）家人共有一种疾病，而且离开这个环境后，症状就有明显变化和好转。  （5）家人常有皮肤过敏等毛病，而且是群发性的。 （6）其他 |
| **18E02** | 是否存在以下家庭环境污染的因素？**（可多选，选择选项（2）时请回答18E03，其余跳至18E04）**  （1）无 （2）宝宝12月龄到18月龄间进行过房屋装修或添置新家具 （3）采暖、烹饪产生油烟 （4）（基本）不进行房屋通风换气  （5）家中宠物（基本）不进行定期健康检查 （6）其他，请注明 |
| **18E03** | 房屋装修或添置新家具的时间： 年 月 日 |
| **18E04** | 宝宝在室内是否受到噪音的困扰？ （1）否 （2）是 （99）不详 |
| **18E05** | 宝宝生活环境中是否存在以下辐射来源？**（可多选）** （1）否 （2）卫星信号接收站 （3）广播发射塔 （4）电视发射台 （5）高压输变电线路 （6）其他，请注明 （99）不详 |
| **18E06** | 宝宝生活范围内，是否有人患传染病？ （1）否 （2）是 （99）不详 |
| **18E07** | 传染病患者与宝宝的关系是？ |
| **18E08** | 是何种传染病？**（可多选）** （1）流行性感冒 （2）流行性腮腺炎 （3）百日咳 （4）水痘 （5）麻疹 （6）乙型脑炎 （7）菌痢 （8）流行性脑脊髓膜炎（流脑） （9）病毒性肝炎 （10）结核病 （11）其他，请注明 （99）不详 |

# 表18F：儿童计划免疫表

| **18F01** | 宝宝12月龄到18月龄是否接种了以下疫苗**：**（1）否**（跳至表18F02）** （2）是 | | | | | | | |
| --- | --- | --- | --- | --- | --- | --- | --- | --- |
|  | 疫苗名称 | | 是否接种 | | | 接种日期 | | 接种次数 |
| **18F01-1** | 乙肝疫苗 | |  | | |  | |  |
| **18F01-2** | 卡介苗 | |  | | |  | |  |
| **18F01-3** | 脊灰疫苗 | |  | | |  | |  |
| **18F01-4** | 百白破疫苗 | |  | | |  | |  |
| **18F01-5** | 白破疫苗 | |  | | |  | |  |
| **18F01-6** | 麻风疫苗 | |  | | |  | |  |
| **18F01-7** | 麻腮风疫苗 | |  | | |  | |  |
| **18F01-8** | 麻腮疫苗 | |  | | |  | |  |
| **18F01-9** | 麻疹疫苗 | |  | | |  | |  |
| **18F01-10** | A群流脑疫苗 | |  | | |  | |  |
| **18F01-11** | A+C群流脑疫苗 | |  | | |  | |  |
| **18F01-12** | 乙脑（减毒）活疫苗 | |  | | |  | |  |
| **18F01-13** | 乙脑灭活疫苗 | |  | | |  | |  |
| **18F01-14** | 甲肝减毒活疫苗 | |  | | |  | |  |
| **18F01-15** | 甲肝灭活疫苗 | |  | | |  | |  |
| **18F01-16** | 水痘疫苗 | |  | | |  | |  |
| **18F01-17** | B型流感嗜血杆菌疫苗 | |  | | |  | |  |
| **18F01-18** | 7价肺炎球菌疫苗 | |  | | |  | |  |
| **18F01-19** | 口服轮状病毒疫苗 | |  | | |  | |  |
| **18F01-20** | 其他疫苗 | |  | | |  | |  |
| **18F02** | 宝宝是否在规定时间内接种相应疫苗？ （1）否 （2）是 **（跳至表18F04）** | | | | | | | |
| **18F03** | 宝宝未按期接种疫苗的原因是？ （1）宝宝身体不适，不能接种 （2）家长忘记接种日期 （3）家长认为可不必按期接种 | | | | | | | |
| **18F04** | 宝宝是否有接种禁忌：（1）无 （2）有 （99）不详 | | | | | | | |
| **18F05** | 宝宝是否有传染病史：（1）无 （2）有 （99）不详 | | | | | | | |
| **18F06** | 有无疫苗异常反应史：（1）无**（跳至表18G）** （2）有（共发生过 次） （99）不详 | | | | | | | |
|  | 异常次数 | 疫苗名称 | | 接种月龄 | 异常反应类型 | | 处理措施 | |
| **18F06-1** | 第1次异常 |  | |  |  | |  | |
| **18F06-2** | 第2次异常 |  | |  |  | |  | |
| **18F06-3** | 第3次异常 |  | |  |  | |  | |

# 表18G：1~3岁宝宝家庭养育环境量表

| **因素** | **条目** | （1）从不（2）很少（3）有时（4）经常（5）总是 | **因素** | **条目** | （1）从不（2）很少（3）有时（4）经常（5）总是 |
| --- | --- | --- | --- | --- | --- |
| 情感温暖/环境气氛 | G01对宝宝需求作出及时回应 |  | 语言/认知 | G25宝宝拥有足量适宜的图书 |  |
|  | G02觉得宝宝可爱，喜欢和宝宝在一起 |  |  | G26了解宝宝玩具的玩法与功用 |  |
|  | G03经常与宝宝肢体接触或目光交流 |  |  | G27与宝宝一起阅读 |  |
|  | G04亲吻、拥抱、爱抚宝宝 |  |  | G28带宝宝进商店、公园、展览 |  |
|  | G05宝宝房间明亮多彩 |  |  | G29有多个宝宝活动的房间 |  |
|  | G06了解宝宝的不开心的原因 |  |  | G30训练宝宝语言和发音 |  |
|  | G07对家庭生活感到满意 |  |  | G31宝宝拥有足够合适的玩具 |  |
|  | G08家庭气氛融洽 |  |  | G32引导宝宝认知形状、颜色、数字 |  |
|  | G09带宝宝外出活动 |  |  | G33参加专业的亲子活动 |  |
|  | G10重视宝宝的早期潜能开发活动 |  |  | G34和宝宝一起听音乐 |  |
|  | G11宝宝房间整洁 |  |  |  |  |
| 社会适应/自理 | G12让宝宝白己穿衣 |  | 忽视/惩罚 | G35惩罚宝宝 |  |
|  | G13鼓励宝宝做家务 |  |  | G36用恐怖意象吓唬孩子 |  |
|  | G14鼓励宝宝做事情 |  |  | G37家长吵架闹别扭 |  |
|  | G15引导宝宝做手工操作 |  |  | G38对宝宝忽冷忽热 |  |
|  | G16让宝宝自己吃饭 |  |  | G39限制宝宝各种活动 |  |
|  | G17鼓励宝宝用笔图画 |  |  | G40带宝宝很累很烦 |  |
|  | G18耐心解答宝宝提问 |  |  | G41不太理睬宝宝 |  |
|  | G19正确引导规范宝宝行为 |  |  | | |
|  | G20引导宝宝观察认识事物 |  |  |  |  |
|  | G21给宝宝讲故事 |  |  |  |  |
|  | G22 鼓励宝宝用语言表达需求 |  |  |  |  |
|  | G23 训练宝宝排便习惯 |  |  |  |  |
|  | G24和其他宝宝玩耍游戏 |  |  |  |  |

# 表18H：0~2岁儿童父母育儿评估量表

| **因素** | **条目** | （1）是（2）否  （3）不清楚 | **因素** | **条目** | （1）是（2）否  （3）不清楚 |
| --- | --- | --- | --- | --- | --- |
| 育儿观念 | H01家长是否知道什么样的宝宝是健康的 |  | 语言刺激 | H21家长是否有意识地播放或哼唱音乐给宝宝听 |  |
|  | H02家长是否能够发现自己宝宝的特点 |  |  | H22家长对宝宝的发生用语音进行回应 |  |
|  | H03家长是否能够在日常生活中为宝宝提供学习的机会 |  |  | H23告诉宝宝物体名称或来访客人的称谓 |  |
|  | H04家长是否认为父母应该担负起养育宝宝的主要责任 |  |  | H24家长对宝宝说话时，是否能使用正确的语句，发音清晰 |  |
|  | H05家长是否能够认识到自己在育儿过程中有哪些不足，并自觉改进 |  | 外出活动 | H25家长是否经常让宝宝与亲友相处，培养人际交往的能力 |  |
| 关注 | H06访谈期间，家长是否注视自己的宝宝并有话语交流、爱抚动作 |  |  | H26家长是否为宝宝提供与其他小朋友接触、玩耍的机会 |  |
|  | H07当家长做家务劳动时是否和宝宝进行交谈 |  |  | H27家长是否带宝宝去郊游或旅行 |  |
|  | H08家长是否关注、欣赏并鼓励宝宝的进步和成长 |  |  | H28家长是否经常带宝宝进行户外活动 |  |
|  | H09宝宝哭闹时，家长是否及时查找原因，并适当处理 |  | 喂养 | H29家长是否在宝宝6月龄时及时添加辅食 |  |
|  | H10家长是否了解宝宝生病的表现 |  |  | H30家长是否知道辅食添加的顺序 |  |
| 亲子关系 | H11访谈期间，家长自然地赞扬宝宝至少2次 |  |  | H31家长给宝宝添加的辅食是否营养全面 |  |
|  | H12宝宝需要临时看护时，是否由熟悉的人来看护 |  |  | H32家长是否采用积极的喂养方式 |  |
|  | H13家长每天与宝宝相处时间不少于2小时 |  | 安全 | H33家长是否注意居住环境的环保 |  |
|  | H14全家经常在一起吃饭并有良好的气氛 |  |  | H34室内是否有安全防护措施 |  |
|  | H15父亲每天也照顾宝宝 |  |  | H35药物、清洁剂、化妆品、锐器、电插座等是否在宝宝够不到的地方 |  |
| 学习材料 | H16宝宝是否拥有能帮助学习颜色、形状、大小的玩具 |  |  | H36家长是否知道紧急救护儿童的知识 |  |
|  | H17宝宝是否拥有锻炼大动作及精细动作的玩具 |  |  | | |
|  | H18家中是否有宝宝自己的图画书 |  |  |  |  |
|  | H19室内是否有符合儿童特点的装饰、图画和用具 |  |  |  |  |
|  | H20宝宝有专门存放玩具或物品的地方 |  |  |  |  |

# 表18I：0~3岁儿童心智发育进程记录

| **序号** | **心智发育项目** | **实际表现月龄** |
| --- | --- | --- |
| I01 | 伏卧抬头45度，能注意父母面部 |  |
| I02 | 伏卧抬头90度 |  |
| I03 | 笑出声、尖叫声、应答性发声 |  |
| I04 | 抱坐时头稳定，能翻身，能手握手、握住摇荡鼓 |  |
| I05 | 伸手抓物 |  |
| I06 | 独坐 |  |
| I07 | 会爬 |  |
| I08 | 听得懂自己的名字，叫名字有反应 |  |
| I09 | 扶东西站 |  |
| I10 | 咿呀学语，躲猫猫，会拍手、摇手表示再见等 |  |
| I11 | 用拇食指捏小物 |  |
| I12 | 有意识叫爸爸、妈妈 |  |
| I13 | 独立行走 |  |
| I14 | 用杯喝水 |  |
| I15 | 走的稳，能叠两块积木 |  |
| I16 | 能说三个字短语 |  |
| I17 | 能走梯 |  |
| I18 | 理解指出身体部分 |  |
| I19 | 会用语言表示大小便 |  |
| I20 | 两脚并跳 |  |
| I21 | 说出自己姓名 |  |
| I22 | 能双脚交替上楼 |  |
| I23 | 会扣纽扣，会折纸，能一页页翻书 |  |
