# Supplementary material for: Effect of feeding patterns on growth and nutritional status of children aged 0-24 months: A Chinese cohort study
Source: PLoS One. 2019 Nov 19;14(11):e0224968. doi: 10.1371/journal.pone.0224968 (PMC6863544; doi:10.1371/journal.pone.0224968)
Supplement: S2 Text — (ZIP) [file pone.0224968.s002.zip › 24╘┬┴Σ.doc]

**编号： □□□□□□**

**2016年开福区社区母婴健康信息采集（24月龄）**

长沙市开福区	社区卫生服务中心

居住地址：	区（县）	街道（路）	社区

母亲姓名：	联系电话：

父亲姓名：	联系电话：

子女姓名：	子女性别：

子女出生日期：

信息收集时间：	年	月	日	信息收集方式：

信息收集人：	信息核查人：

目录

表24A：24月龄儿童随访记录表	3

表24B：儿童睡眠、视力、口腔、行为等情况调查表	5

表24C：儿童患病情况	7

表24D：宝宝添加辅食及喂养行为情况	8

表24E：儿童环境危险因素调查表	9

表24F：儿童计划免疫表	10

表24G：1~3岁宝宝家庭养育环境量表	12

表24H：0~2岁儿童父母育儿评估量表	13

表24I：0~3岁儿童心智发育进程记录	14

表24J：孤独症儿童行为量表（ABC量表）	15

# 表24A：24月龄儿童随访记录表

| **24A01** | 检查日期： （ 年 月 日） |
| --- | --- |
| **24A02** | 实足月龄：（ 月龄 天） |
| **24A03** | 体重：（ **Kg**） |
| **24A04** | 身长：（ **cm**） |
| **24A05** | 头围：（ **cm**） |
| **24A06** | 宝宝前囟是否闭合？（1）否 （2）是**（跳至24A08）** |
| **24A07** | 前囟： cm× cm |
| **24A08** | 宝宝前囟闭合时间： 月龄至 月龄 |
| **24A09** | 宝宝的出牙数：（ 颗） |
| **24A10** | 体格检查： （1）正常**（跳至24A12）** （2）异常 |
| **24A11** | 体格检查异常为： |
| **24A12** | 目前家里有 个孩子 |
| **24A13** | 宝宝18月龄到24月龄的主要看护人是**（可多选）**？  （1）母亲 （2）父亲 （3）（外）祖母 （4）（外）祖父 （5）保姆（钟点工） （6）亲戚或朋友 （7）其他，请注明________________ |
| **24A14** | 父亲平均每天照顾宝宝的时间：（1）少于1小时 （2）1~3小时 （3）3~5小时 （4）5~8小时 （5）8~12小时 （6）12小时以上 |
| **24A15** | 母亲平均每天照顾宝宝的时间：（1）少于1小时 （2）1~3小时 （3）3~5小时 （4）5~8小时 （5）8~12小时 （6）12小时以上 |
| **24A16** | 喂养方式： （1）纯母乳喂养 （2）人工喂养**（跳至24A18）** （3）混合喂养 |
| **24A17** | 母乳次数： （ 次/日） |
| **24A18** | 宝宝是否已断母乳？（1）否**（跳至24A20）** （2）是，断母乳的月龄是 月 天 |
| **24A19** | 您给宝宝断奶的原因是什么？**（可多选，选任一项均跳至24A21）**（1）工作需求 （2）生病 （3）无母乳（4）觉得母乳喂养麻烦 （5）担心影响身材或形象 （6）认为配方奶粉更有营养（7）宝宝生病（8）宝宝拒绝吸吮（9）觉得宝宝已到断乳年龄（10）哺乳时宝宝会咬乳头（11）其他，请注明： |
| **24A20** | 打算母乳喂养多久？ 月 |
| **24A21** | 宝宝18月龄到24月龄是否使用带奶嘴的奶瓶喝水、奶类或果汁等？ （1）否**（跳至24A23）** （2）偶尔 （3）经常 |
| **24A22** | 宝宝开始使用带奶嘴的奶瓶喝水、奶类或果汁等的时间： 月龄 |
| **24A23** | 宝宝18月龄到24月龄期间，是否有含奶嘴（安抚奶嘴除外）入睡的习惯？ （1）否**（跳至24A26）** （2）偶尔 （3）经常 |
| **24A24** | 宝宝何时开始含奶嘴（安抚奶嘴除外）入睡？ 月龄 |
| **24A25** | 宝宝含奶嘴睡觉持续的时间？（1）0.5小时以内 （2）0.5~1小时 （3）1小时以上 （4）整晚 |
| **24A26** | 宝宝18月龄到24月龄是否使用安抚奶嘴？ （1）否**（跳至24A29）** （2）是 |
| **24A27** | 宝宝开始使用安抚奶嘴的时间： 月龄 |
| **24A28** | 宝宝通常在什么情况下使用安抚奶嘴**（可多选）**？（1）宝宝哭闹时 （2）宝宝睡觉时 （3）宝宝咬手指时 （4）帮助宝宝锻炼口腔吸吮和吞咽能力时  （5）防止宝宝“奶瓶龋” （6）帮助宝宝断母乳 （7）其他，（请注明）： |
| **24A29** | 宝宝18月龄到24月龄间，您是否给宝宝添加配方奶粉或鲜奶？ （1）未添加**（跳至24A31）** （2）已添加，首次添加时间为： 月龄 天 |
| **24A30** | 配方奶粉或鲜奶情况： （ 次/天），每次 **ml** |
| **24A31** | 宝宝18月龄到24月龄是否做过微量元素的检测？（1）否**（跳至24A32）** （2）是 |
| **24A31-1** | 检测月龄： ，微量元素检测结果是否正常？（1）正常 （2）缺钙 （3）缺铁 （4）缺锌 （5）缺镁 （6）铜超标 （7）铅超标 （8）其他 |
| **24A31-2** | 检测月龄： ，微量元素检测结果是否正常？（1）正常 （2）缺钙 （3）缺铁 （4）缺锌 （5）缺镁 （6）铜超标 （7）铅超标 （8）其他 |
| **24A31-3** | 检测月龄： ，微量元素检测结果是否正常？（1）正常 （2）缺钙 （3）缺铁 （4）缺锌 （5）缺镁 （6）铜超标 （7）铅超标 （8）其他 |
| **24A32** | 宝宝出生到24月龄是否做过维生素D的检测：（1）否 **（跳至24A33）** （2）是 |
| **24A32-1** | 检测月龄： ，检测结果是否正常？（1）正常 （2）缺乏 |
| **24A33** | 宝宝18月龄到24月龄补充钙剂的频率：（1）基本不吃 （2）每月吃1~2次 （3）每周吃1~2次 （4）每周吃3~4次 （5）每周吃≥5次 |
| **24A34** | 宝宝18月龄到24月龄服用维生素D的频率：（1）基本不吃 （2）每月吃1~2次 （3）每周吃1~2次 （4）每周吃3~4次 （5）每周吃≥5次 |
| **24A35** | 宝宝18月龄到24月龄是否补充其他营养补充剂? （1）否**（跳至24A36）**（2）是，请注明： |
| **24A35-1** | 宝宝补充其他营养补充剂的时间： 月龄至 月龄 |
| **24A36** | 宝宝18月龄到24月龄平均每周户外活动的天数：（1）0天**（跳至24A38）** （2）1~2天 （3）3~5天 （4）6~7天 （99）不详 |
| **24A37** | 宝宝平均每天户外活动的时间： 小时/日 |
| **24A38** | 在宝宝18月龄到24月龄间，宝宝父母是否参加过育儿方面的培训？（1）否**（跳至24A40）** （2）是 |
| **24A39** | 宝宝父母参加育儿培训的原因是**（可多选）**： （1）学习科学的育儿知识和方法 （2）可以解决育儿方面的困惑 （3）有利于宝宝的教育和成长  （4）有利于更好地做好父母的角色 （5）可以减少育儿过程中的心理焦虑 （6）其他，请注明 |
| **24A40** | 宝宝是否参加早教班？（1）否 （2）是**（跳至24A42）** |
| **24A41** | 宝宝未参加早教班的原因是**（可多选，选任一项均跳至24A46）：**（1）宝宝年龄太小，不想增加其负担 （2）早教班费用较高 （3）统一教学无法因人而异构 （4）早教班老师素质良莠不齐 （5）担心过度早教会影响宝宝未来的学习 （6）其他，请注明 |
| **24A42** | 宝宝开始参加早教班的时间： 月龄 |
| **24A43** | 宝宝参加早教班的原因是**（可多选）：**（1）有利于提高宝宝的认知、语言表达、运动、社会交往等能力 （2）有利于开发宝宝的潜能 （3）早教的课程内容可以作为教养的指导 （4）可以为宝宝上幼儿园打基础 （5）没有时间照看宝宝 （6）其他，请注明 |
| **24A44** | 宝宝18月龄到24月龄间，参加早教班的次数： 次 |
| **24A45** | 您觉得宝宝参加早教班有效果吗？（1）没有 （2）有，请注明 |
| **24A46** | 宝宝出生到24月龄间，宝宝父母是否对宝宝进行早期教育活动？（1）否**（跳至24A49）** （2）有 （**早期教育：指在出生至上学前阶段，家长以培养孩子感知、动作、语言、思维等为目标，结合孩子的 生理和心理发展特点，利用客观外界环境和教育训练方式对孩子开展相关教育。**） |
| **24A47** | 宝宝父母对宝宝开始早期教育的时间： 月龄 |
| **24A48** | 宝宝父母对宝宝进行早期早教的内容为**（可多选）**：（1）认知方面 （2）语言表达方面 （3）社交方面 （4）运动方面 （5）动手操作方面  （6）其他，请注明 |
| **24A49** | 宝宝18月龄到24月龄间，宝宝父亲是否吸烟？ （1）否**（跳至24A51）** （2）是 |
| **24A50** | 宝宝父亲吸烟量： 支/日**（1包烟=20支烟）** |
| **24A51** | 宝宝18月龄到24月龄间，宝宝父亲是否饮酒？ （1）否**（跳至24A54）** （2）是 |
| **24A52** | 宝宝父亲饮酒频率： 次/周 |
| **24A53** | 宝宝父亲的饮酒量： **g**/次  （各类酒的饮酒量均折算成高度白酒计算，1瓶啤酒或1斤米酒≈1两高度白酒，1斤葡萄酒或黄酒≈2.5两高度白酒，2两低度白酒 ≈1.5两高度白酒，1两谷酒≈1两高度白酒，1两=50g，高度白酒或谷酒45~60%（v/v），低度白酒35~44%（v/v），葡萄酒或黄酒12~24%（v/v），啤酒或米酒4~6%（v/v）） |
| **24A54** | 宝宝18月龄到24月龄间，您是否吸烟？ （1）否**（跳至24A56）** （2）是 |
| **24A55** | 您的吸烟量： 支/日**（1包烟=20支烟）** |
| **24A56** | 宝宝18月龄到24月龄间，您是否饮酒？ （1）否**（跳至24A59）** （2）是 |
| **24A57** | 您的饮酒频率： 次/周 |
| **24A58** | 您的饮酒量： **g**/次  **（**各类酒的饮酒量均折算成高度白酒计算，1瓶啤酒或1斤米酒≈1两高度白酒，1斤葡萄酒或黄酒≈2.5两高度白酒，2两低度白酒 ≈1.5两高度白酒，1两谷酒≈1两高度白酒，1两=50g，高度白酒或谷酒45~60%（v/v），低度白酒35~44%（v/v）葡萄酒或黄酒12~24%（v/v），啤酒或米酒4~6%（v/v）） |
| **24A59** | 目前，宝宝平均每周被动吸烟的天数（每天超过15分钟）：（1）0天**（跳至24B）** （2）1~2天 （3）3~5天 （4）6~7天 （99）不详 |
| **24A60** | 宝宝被动吸烟的主要来源是**（可多选）:** （1）家里 （2）小区 （3）公园 （4）游乐园 （5）其他，请注明 ： （99）不详 |

# 表24B：儿童睡眠、视力、口腔、行为等情况调查表

| **24B01** | 宝宝的睡眠：（1）正常 （2）异常，(如入睡困难，频繁夜醒，睡眠节律紊乱) |
| --- | --- |
| **24B02** | 睡眠总时间: 小时/日 ，夜间睡眠时间： 小时/日 |
| **24B03** | 宝宝入睡方式？ （1）自行入睡 （2）依赖外界帮助，如抱睡、摇晃入睡、吃奶入睡等。 |
| **24B04** | 宝宝晚上是否与他人一起睡？（1）否**（跳至24B06）** （2）是 |
| **24B05** | 宝宝晚上与谁一起睡**（可多选）**？（1）母亲（2）父亲（3）（外）祖母（4）（外）祖父（5）保姆（钟点工）（6）亲戚或朋友（7）其他，请注明 |
| **24B06** | 宝宝睡前情绪是否稳定？（1）否 （2）是 |
| **24B07** | 宝宝睡觉的房间是否有电视？（1）否 （2）是 |
| **24B08** | 宝宝是否对手机、iPad、电视等电子产品感兴趣？ （1）否**（跳至24B12）** （2）偶尔 （3）经常 |
| **24B09** | 宝宝通常会看哪些电子产品**（可多选）**？（1）手机 （2）电视 （3）iPad （4）游戏机 （5）计算机 （6）其他，请注明________________ |
| **24B10** | 宝宝通常会在什么情况下看手机、iPad、电视等电子产品**（可多选）?** （1）宝宝学习巧虎等早教视频 （2）宝宝哭闹时，用于安抚 （3）看护人为了省心，主动让宝宝使用 （4）大人在使用这类电子产品时，宝宝在旁边看 （5）宝宝入睡前 （6）其他，请注明________________________ |
| **24B11** | 宝宝每天注视电子屏幕的时间： 小时/日 |
| **24B12** | 宝宝18月龄到24月龄是否测过视力？（1）否**（跳至24B13）** （2）是 |
| **24B12-1** | 检测月龄： ，视力是否正常：（1）正常 （2）近视（3）远视（4）斜视（5）弱视 （6）散光 （7）其他，请注明________ |
| **24B12-2** | 检测月龄： ，视力是否正常：（1）正常 （2）近视（3）远视（4）斜视（5）弱视 （6）散光 （7）其他，请注明________ |
| **24B12-3** | 检测月龄： ，视力是否正常：（1）正常 （2）近视（3）远视（4）斜视（5）弱视 （6）散光 （7）其他，请注明________ |
| **24B13** | 宝宝18月龄到24月龄是否检查过口腔（牙齿）问题？（1）否**（跳至24B14）** （2）是 |
| **24B13-1** | 检测月龄： 口腔是否正常：（1）正常 （2）龋齿 （3）其他，请注明________ |
| **24B13-2** | 检测月龄： 口腔是否正常：（1）正常 （2）龋齿 （3）其他，请注明________ |
| **24B13-3** | 检测月龄： 口腔是否正常：（1）正常 （2）龋齿 （3）其他，请注明________ |
| **24B14** | 是否给宝宝喂嚼碎的食物？（1）否 （2）偶尔 （3）经常 |
| **24B15** | 宝宝每日吃零食次数？（1）0次**（跳至24B18）** （2）1~2次 （3）3次及以上 **（****零食：指非正餐时间食用的各种少量的食物和 /或饮料（不包括水））** |
| **24B16** | 宝宝是否每日定时吃零食？（1）定时 （2）不定时 |
| **24B17** | 宝宝吃零食的种类是**（可多选）**：（1）膨化食品 （2）糖果类 （3）谷类 （4）饮料类（5）新鲜水果蔬菜 （6）坚果类 （7）肉蛋类 （8）海产品  （9）油炸类 （10）薯类 （11）奶类及制品 （12）饼干 （13）其他，请注明_______ |
| **24B18** | 宝宝睡前是否吃含糖甜食？（1）否 （2）有时 （3）经常 |
| **24B19** | 宝宝是否已经开始刷牙？（1）否**（跳至表24B24）** （2）是 |
| **24B20** | 宝宝开始刷牙的时间：_____ 月龄 |
| **24B21** | 宝宝每天刷牙次数？（1）早或晚一次 （2）早晚刷牙 （3）不固定 |
| **24B22** | 宝宝是否使用含氟儿童牙膏？（1）否 （2）偶尔 （3）经常 |
| **24B23** | 宝宝18月龄到24月龄是否吸吮手指？（1）否**（跳至24C）** （2）有时 （3）经常 |
| **24B24** | 宝宝开始吸吮手指的时间：_____月龄 |
| **24B25** | 宝宝吸吮手指的习惯已持续多久？ 月 |
| **24B26** | 宝宝通常会在什么情况下吸吮手指？（1）宝宝一个人单独玩时 （2）宝宝饥饿时 （3）宝宝睡觉时 （4）习惯性动作 （5）其他，请注明 |
| **24B27** | 宝宝吸吮手指时，您是否会阻止？（1）否 （2）偶尔会 （3）每次都会 |

# 表24C：儿童患病情况

| **24C01** | 宝宝18月龄到24月龄间是否对某种或某类物质的过敏？（1）否**（跳至24C02）** （2）是 | | | | | | | | | | | | | | | | |
| --- | --- | --- | --- | --- | --- | --- | --- | --- | --- | --- | --- | --- | --- | --- | --- | --- | --- |
| **24C01-1** | 如果发生过物质过敏，是哪种或哪些物质**（可多选）？**  （1）牛奶 （2）奶酪 （3）全蛋 （4）尘螨 （5）家尘 （6）牛肉 （7）鱼虾蟹贝蛤 （8）狗毛鸡毛 （9）蟑螂  （10）橙 （11）各类真菌 （12）黄豆 （13）各类花粉 （14）花生 （15）青胡椒 （16）小麦 （17）鸡肉 （18）蘑菇  （19）其他（请注明） （99）不详 | | | | | | | | | | | | | | | | |
| **24C02** | 宝宝18月龄到24月龄间有无药物过敏情况：（1）无**（跳至24C03）** （2）有 | | | | | | | | | | | | | | | | |
|  | 发生次数 | | | 发生时间 | | | 药物名称 | | | 给药途径  （1）静脉滴注 （2）皮下注射 （3）静脉注射（4）肌肉注射（5）口服给药（99）不详 | | | | | | | |
| **24C02-1** | 第1次 | | |  | | |  | | |  | | | | | | | |
| **24C02-2** | 第2次 | | |  | | |  | | |  | | | | | | | |
| **24C02-3** | 第3次 | | |  | | |  | | |  | | | | | | | |
| **24C03** | 宝宝18月龄到24月龄间是否患过敏性疾病？ （1）否**（跳至24C04）** （2）是 | | | | | | | | | | | | | | | | |
| **24C03-1** | 如果患过过敏性疾病，是哪种或哪些**（可多选）**？  （1）湿疹 （2）过敏性皮炎 （3）过敏性鼻炎 （4）过敏性哮喘 （5）过敏性紫癜 （6）过敏性休克 （7）其他(请注明) | | | | | | | | | | | | | | | | |
| **24C04** | 宝宝18月龄到24月龄间是否患过传染病**?** （1）否**（跳至24C05）** （2）是 | | | | | | | | | | | | | | | | |
| **24C04-1** | 如果患过传染病，是哪种或哪些？**（可多选）** （1）水痘 （2）流行性腮腺炎 （3）风疹 （4）麻疹 （5）手足口病 （6）病毒性肝炎 （7）肺结核  （8）疱疹性咽峡炎 （9）幼儿急疹 （10）其他传染病（请注明） （99）不详 | | | | | | | | | | | | | | | | |
| **24C05** | 宝宝18月龄到24月龄间是否患过除过敏性疾病和传染病之外的其他疾病？ （1）否**（跳至24C06）** （2）是 | | | | | | | | | | | | | | | | |
| **24C05-1** | 如果患过，是哪种或哪些？**（可多选）**  （1）急性上呼吸道感染（感冒） （2）腹泻 （3）支气管炎（4）肺炎（5）哮喘（6）佝偻病（7）贫血（8）龋齿（9）中耳炎 （10）其他(请注明) | | | | | | | | | | | | | | | | |
| **24C05-2** | 若患过感冒，患感冒次数： 次 | | | | | | | | | |  | | | | | | |
| **24C05-3** | 若患过腹泻，患腹泻次数： 次 | | | | | | | | | |  | | | | | | |
| **24C06** | 宝宝18月龄到24月龄间是否有因病就医行为？ （1）否**（跳至24C07）** （2） 是 | | | | | | | | | | | | | | | | |
|  | 就诊次数 | 就诊月龄 | | | 就诊单位名称 | | | 就诊单位类型  （1）药店（2）诊所（3）医院  （4）社区卫生服务中心（5）其他 | | | | | 最终诊断 | 主要治疗药物 | 其他处理 | | 疾病转归  （1）痊愈（2）好转（3）无变化 （4）恶化 （5）其他 |
| **24C06-1** | 第1次 |  | | |  | | |  | | | | |  |  |  | |  |
| **24C06-2** | 第2次 |  | | |  | | |  | | | | |  |  |  | |  |
| **24C06-3** | 第3次 |  | | |  | | |  | | | | |  |  |  | |  |
| **24C06-4** | 第4次 |  | | |  | | |  | | | | |  |  |  | |  |
| **24C07** | 宝宝18月龄到24月龄是否发生过意外伤害**（凡具有下列两种情况之一者一种情况者可作为伤害：①到医疗单位诊断为某一类损伤；②因伤休息（休工或休学）一日以上）**？ （1）否**（跳至表24D）** （2）是 | | | | | | | | | | | | | | | | |
| **24C08** | 如果发生过，是哪种或哪些？**（可多选，并分别将代码按发生时间填入伤害类别中）**  （1）车祸 （2）跌落 （3）钝器伤 （4）烧烫伤 （5）利器伤 （6）意外中毒 （7）异物窒息 （8）触电（9）动物咬伤（10）溺水  （11）其他（请注明） （99）不详 | | | | | | | | | | | | | | | | |
|  | 发生  次数 | | 伤害类别 | | | 发生月龄 | | | 处理伤害方式  （1）家庭处理（2）送医处理  （3）未加处理（99）不详 | | | 伤害发生时的看护人  （1）无（2）父母亲（3）（外）祖父母（4）保姆或护工（5）亲属或朋友（6）其他（99）不详 | | | | 现在宝宝恢复情况？  （1）尚不乐观（2）明显好转  （3）完全恢复（99）不详 | |
| **24C08-1** | 第1次 | |  | | |  | | |  | | |  | | | |  | |
| **24C08-2** | 第2次 | |  | | |  | | |  | | |  | | | |  | |
| **24C08-3** | 第3次 | |  | | |  | | |  | | |  | | | |  | |
| **24C08-4** | 第4次 | |  | | |  | | |  | | |  | | | |  | |
| **24C08-5** | 第5次 | |  | | |  | | |  | | |  | | | |  | |
| **24C08-6** | 第6次 | |  | | |  | | |  | | |  | | | |  | |
| **24C08-7** | 第7次 | |  | | |  | | |  | | |  | | | |  | |
| **24C08-8** | 第8次 | |  | | |  | | |  | | |  | | | |  | |
| **24C08-9** | 第9次 | |  | | |  | | |  | | |  | | | |  | |

# 表24D：宝宝添加辅食及喂养行为情况

| **24D01** | 宝宝18月龄到24月龄食用的主食是什么？ | | | |
| --- | --- | --- | --- | --- |
| **24D02** | 昨天（包括白天和夜里），宝宝吃过辅食的种类： 种。 | | | |
| **24D03** | 昨天（包括白天和夜里），宝宝吃过 餐次辅食 (填次数，辅食指正餐和零食） | | | |
|  | **食物品种** | **食用频率** | **频率** | **开始添加月龄** |
| **24D03-1** | 谷薯类 | （1）基本不吃 （2）每天吃1次 （3）每天吃2次 （4）每天吃≥3次 |  |  |
| **24D03-2** | 奶类及制品 | （1）基本不吃 （2）每月吃1~2次（3）每周吃1~2次 （4）每周吃3~4次（5）每周吃≥5次 |  |  |
| **24D03-3** | 蛋类 | （1）基本不吃 （2）每月吃1~2次（3）每周吃1~2次 （4）每周吃3~4次（5）每周吃≥5次 |  |  |
| **24D03-4** | 禽畜肉类 | （1）基本不吃 （2）每月吃1~2次（3）每周吃1~2次 （4）每周吃3~4次（5）每周吃≥5次 |  |  |
| **24D03-5** | 鱼类 | （1）基本不吃 （2）每月吃1~2次（3）每周吃1~2次 （4）每周吃3~4次（5）每周吃≥5次 |  |  |
| **24D03-6** | 豆类及制品 | （1）基本不吃 （2）每月吃1~2次（3）每周吃1~2次 （4）每周吃3~4次（5）每周吃≥5次 |  |  |
| **24D03-7** | 新鲜水果 | （1）基本不吃 （2）每月吃1~2次（3）每周吃1~2次 （4）每周吃3~4次（5）每周吃≥5次 |  |  |
| **24D03-8** | 新鲜蔬菜 | （1）基本不吃 （2）每月吃1~2次（3）每周吃1~2次 （4）每周吃3~4次（5）每周吃≥5次 |  |  |
| **24D03-9** | 动物内脏 | （1）基本不吃 （2）每月吃1~2次（3）每周吃1~2次 （4）每周吃3~4次（5）每周吃≥5次 |  |  |
| **24D03-10** | 坚果 | （1）基本不吃 （2）每月吃1~2次（3）每周吃1~2次 （4）每周吃3~4次（5）每周吃≥5次 |  |  |
| **24D03-11** | 食用油 | （1）基本不吃 （2）每月吃1~2次（3）每周吃1~2次 （4）每周吃3~4次（5）每周吃≥5次 |  |  |
| **24D04** | 平时单独给宝宝做饭吗？ （1）不会 （2）偶尔 （3）有时 （4）经常 （5）总是 | | | |
| **24D05** | 同一种食物您会用不同的做法来促进宝宝食欲吗？ （1）没有 （2）偶尔 （3）有时 （4）经常 （5）总是 | | | |
| **24D06** | 宝宝进食前，您会给宝宝洗手吗? （1）不会 （2）偶尔 （3）有时 （4）经常 （5）总是 | | | |
| **24D07** | 宝宝进食时，您和宝宝有语言性鼓励和感情交流吗？ （1）没有 （2）偶尔 （3）有时 （4）经常 （5）总是 | | | |
| **24D08** | 您会训练宝宝自己拿勺子或者筷子吃饭吗？ （1）不会 （2）偶尔 （3）有时 （4）经常 （5）总是 | | | |
| **24D09** | 当宝宝拒绝某种食物时，您会： （1）不再喂食该食物 （2）暂时放弃，下次再尝试 （3）温柔地鼓励宝宝尝试 （4）强迫或追着宝宝喂食 | | | |
| **24D10** | 宝宝进食时，是否会出现以下行为**（可多选，选择选项（2）时请回答24D11，其余跳至24D12）**： （1）否 （2）对食物不感兴趣 （3）强烈偏爱某些食物 （4）拒绝某些食物 （5）进餐分心 （6）进餐地点不固定 （7）进餐时间超过30分钟 （8）其他，请注明 | | | |
| **24D11** | 宝宝对食物不感兴趣的原因是？ （1）宝宝过多吃零食 （2）宝宝活动量不足 （3）宝宝消化不良 （4）宝宝缺锌 （5）其他，请注明 | | | |
| **24D12** | 宝宝在进食时有无电视、玩具、手机等的干扰？ （1）无 （2）偶尔 （3）有时 （4）经常 （5）总是 | | | |

# 表24E：儿童环境危险因素调查表

| **家庭内部环境调查** | |
| --- | --- |
| **24E01** | 宝宝18月龄至24月龄间，是否存在室内污染现象？（1）否 **（跳至表24E02）** （2）是 |
| **24E01-1** | 如果存在室内污染现象，是哪种或哪些？**（可多选）**  （1）每天清晨起床时，感到[憋闷](http://zhidao.baidu.com/search?word=憋闷&fr=qb_search_exp&ie=utf8)、恶心、甚至头晕目眩。  （2）家里小孩常咳嗽、打喷嚏、免疫力下降，新装修的房子宝宝不愿回家。  （3）家人共有一种疾病，而且离开这个环境后，症状就有明显变化和好转。  （4）家人常有皮肤过敏等毛病，而且是群发性的。 （5）其他，请注明 |
| **24E02** | 宝宝18月龄至24月龄间，是否存在家庭环境污染的因素？（1）否 **（跳至表24E03）** （2）是 |
| **24E02-1** | 如果存在家庭环境污染因素，是哪种或哪些？**（可多选，选择选项（1）时请回答24E02-2，其余跳至24E03）**  （1）宝宝18月龄到24月龄间进行过房屋装修或添置新家具 （2）采暖、烹饪产生油烟 （3）（基本）不进行房屋通风换气  （4）家中宠物（基本）不进行定期健康检查 （5）其他，请注明 |
| **24E02-2** | 房屋装修或添置新家具的时间： 年 月 日 |
| **24E03** | 宝宝18月龄至24月龄间，在室内是否受到噪音的困扰？ （1）否 （2）是 （99）不详 |
| **24E04** | 宝宝18月龄至24月龄间，生活环境中是否存在辐射来源？（1）否 **（跳至表24E05）** （2）是 |
| **24E04-1** | 如果存在辐射来源，是哪种或哪些？**（可多选）**  （1）卫星信号接收站 （2）广播发射塔 （3）电视发射台 （4）高压输变电线路 （5）其他，请注明 （99）不详 |
| **24E05** | 宝宝18月龄至24月龄间，其生活范围内是否有人患传染病？ （1）否 （2）是 （99）不详 |
| **24E05-1** | 传染病患者与宝宝的关系是？ |
| **24E05-2** | 是何种传染病？**（可多选）** （1）流行性感冒 （2）流行性腮腺炎 （3）百日咳 （4）水痘 （5）麻疹 （6）乙型脑炎 （7）菌痢 （8）流行性脑脊髓膜炎（流脑） （9）病毒性肝炎 （10）结核病 （11）其他，请注明 （99）不详 |

# 表24F：儿童计划免疫表

| **24F01** | 目前您的孩子是否接种了以下哪些疫苗？ | | | | |
| --- | --- | --- | --- | --- | --- |
| **24F01-1** | 乙肝疫苗： ⑴否 ⑵是 接种日期1： 接种日期2：  接种日期3： 接种日期4： | | | | |
| **24F01-2** | 卡介苗： ⑴否 ⑵是 接种日期1： 接种日期2： | | | | |
| **24F01-3** | 脊灰疫苗： ⑴否 ⑵是 接种日期1： 接种日期2：  接种日期3： 接种日期4： | | | | |
| **24F01-4** | 百白破疫苗： ⑴否 ⑵是 接种日期1： 接种日期2：  接种日期3： 接种日期4： | | | | |
| **24F01-5** | 白破疫苗： ⑴否 ⑵是 接种日期1： 接种日期2： | | | | |
| **24F01-6** | 麻风疫苗： ⑴否 ⑵是 接种日期1： 接种日期2： | | | | |
| **24F01-7** | 麻腮风疫苗： ⑴否 ⑵是 接种日期1： 接种日期2： | | | | |
| **24F01-8** | 麻腮疫苗： ⑴否 ⑵是 接种日期1： 接种日期2： | | | | |
| **24F01-9** | 麻疹疫苗： ⑴否 ⑵是 接种日期1： 接种日期2： | | | | |
| **24F01-10** | A群流脑疫苗： ⑴否 ⑵是 接种日期1： 接种日期2： | | | | |
| **24F01-11** | A+C群流脑疫苗： ⑴否 ⑵是 接种日期1： 接种日期2： | | | | |
| **24F01-12** | 乙脑（减毒）活疫苗： ⑴否 ⑵是 接种日期1： 接种日期2： | | | | |
| **24F01-13** | 乙脑灭活疫苗： ⑴否 ⑵是 接种日期1： 接种日期2：  接种日期3： 接种日期4： | | | | |
| **24F01-14** | 甲肝减毒活疫苗： ⑴否 ⑵是 接种日期1： 接种日期2： | | | | |
| **24F01-15** | 甲肝灭活疫苗： ⑴否 ⑵是 接种日期1： 接种日期2： | | | | |
| **24F01-16** | 水痘疫苗: ⑴否 ⑵是 接种日期1： 接种日期2： | | | | |
| **24F01-17** | B型流感嗜血杆菌疫苗（HIB）：⑴否 ⑵是 接种日期1： 接种日期2：  接种日期3： 接种日期4： | | | | |
| **24F01-18** | 百白破、灭活脊灰和b型流感 ⑴否 ⑵是 接种日期1： 接种日期2：  嗜血杆菌联合疫苗： 接种日期3： 接种日期4： | | | | |
| **24F01-19** | 7价肺炎球菌疫苗： ⑴否 ⑵是 接种日期1： 接种日期2：  接种日期3： 接种日期4： | | | | |
| **24F01-20** | 口服轮状病毒疫苗： ⑴否 ⑵是 接种日期1： 接种日期2：  接种日期3： 接种日期4： | | | | |
| **24F01-21** | 流感疫苗： ⑴否 ⑵是 接种日期1： 接种日期2： | | | | |
| **24F01-22** | 其他疫苗： ⑴否 ⑵是 疫苗名称： 接种日期：  疫苗名称： 接种日期：  疫苗名称： 接种日期：  疫苗名称： 接种日期： | | | | |
| **24F02** | 宝宝是否在规定时间内接种相应疫苗？ （1）否 （2）是 **（跳至表24F04）** | | | | |
| **24F03** | 宝宝未按期接种疫苗的原因是？ （1）宝宝身体不适，不能接种 （2）家长忘记接种日期 （3）家长认为可不必按期接种 （4）与接种时间冲突 （5）其他，请注明 | | | | |
| **24F04** | 宝宝是否有接种禁忌：（1）无 （2）有 （99）不详 | | | | |
| **24F05** | 宝宝是否有传染病史：（1）无 （2）有 （99）不详 | | | | |
| **24F06** | 有无疫苗异常反应史：（1）无**（跳至表24G）** （2）有（共发生过 次） （99）不详 | | | | |
|  | 异常次数 | 疫苗名称 | 接种月龄 | 异常反应类型 | 处理措施 |
| **24F06-1** | 第1次异常 |  |  |  |  |
| **24F06-2** | 第2次异常 |  |  |  |  |
| **24F06-3** | 第3次异常 |  |  |  |  |

# 表24G：1~3岁宝宝家庭养育环境量表

| **因素** | **条目** | （1）从不（2）很少（3）有时（4）经常（5）总是 | **因素** | **条目** | （1）从不（2）很少（3）有时（4）经常（5）总是 |
| --- | --- | --- | --- | --- | --- |
| 情感温暖/环境气氛 | G01对宝宝需求作出及时回应 |  | 语言/认知 | G25宝宝拥有足量适宜的图书 |  |
|  | G02觉得宝宝可爱，喜欢和宝宝在一起 |  |  | G26了解宝宝玩具的玩法与功用 |  |
|  | G03经常与宝宝肢体接触或目光交流 |  |  | G27与宝宝一起阅读 |  |
|  | G04亲吻、拥抱、爱抚宝宝 |  |  | G28带宝宝进商店、公园、展览 |  |
|  | G05宝宝房间明亮多彩 |  |  | G29有多个宝宝活动的房间 |  |
|  | G06了解宝宝的不开心的原因 |  |  | G30训练宝宝语言和发音 |  |
|  | G07对家庭生活感到满意 |  |  | G31宝宝拥有足够合适的玩具 |  |
|  | G08家庭气氛融洽 |  |  | G32引导宝宝认知形状、颜色、数字 |  |
|  | G09带宝宝外出活动 |  |  | G33参加专业的亲子活动 |  |
|  | G10重视宝宝的早期潜能开发活动 |  |  | G34和宝宝一起听音乐 |  |
|  | G11宝宝房间整洁 |  |  |  |  |
| 社会适应/自理 | G12让宝宝白己穿衣 |  | 忽视/惩罚 | G35惩罚宝宝 |  |
|  | G13鼓励宝宝做家务 |  |  | G36用恐怖意象吓唬孩子 |  |
|  | G14鼓励宝宝做事情 |  |  | G37家长吵架闹别扭 |  |
|  | G15引导宝宝做手工操作 |  |  | G38对宝宝忽冷忽热 |  |
|  | G16让宝宝自己吃饭 |  |  | G39限制宝宝各种活动 |  |
|  | G17鼓励宝宝用笔图画 |  |  | G40带宝宝很累很烦 |  |
|  | G24耐心解答宝宝提问 |  |  | G41不太理睬宝宝 |  |
|  | G19正确引导规范宝宝行为 |  |  | | |
|  | G20引导宝宝观察认识事物 |  |  |  |  |
|  | G21给宝宝讲故事 |  |  |  |  |
|  | G22 鼓励宝宝用语言表达需求 |  |  |  |  |
|  | G23 训练宝宝排便习惯 |  |  |  |  |
|  | G24和其他宝宝玩耍游戏 |  |  |  |  |

# 表24H：0~2岁儿童父母育儿评估量表

| **因素** | **条目** | （1）是（2）否  （3）不清楚 | **因素** | **条目** | （1）是（2）否  （3）不清楚 |
| --- | --- | --- | --- | --- | --- |
| 育儿观念 | H01家长是否知道什么样的宝宝是健康的 |  | 语言刺激 | H21家长是否有意识地播放或哼唱音乐给宝宝听 |  |
|  | H02家长是否能够发现自己宝宝的特点 |  |  | H22家长对宝宝的发生用语音进行回应 |  |
|  | H03家长是否能够在日常生活中为宝宝提供学习的机会 |  |  | H23告诉宝宝物体名称或来访客人的称谓 |  |
|  | H04家长是否认为父母应该担负起养育宝宝的主要责任 |  |  | H24家长对宝宝说话时，是否能使用正确的语句，发音清晰 |  |
|  | H05家长是否能够认识到自己在育儿过程中有哪些不足，并自觉改进 |  | 外出活动 | H25家长是否经常让宝宝与亲友相处，培养人际交往的能力 |  |
| 关注 | H06访谈期间，家长是否注视自己的宝宝并有话语交流、爱抚动作 |  |  | H26家长是否为宝宝提供与其他小朋友接触、玩耍的机会 |  |
|  | H07当家长做家务劳动时是否和宝宝进行交谈 |  |  | H27家长是否带宝宝去郊游或旅行 |  |
|  | H08家长是否关注、欣赏并鼓励宝宝的进步和成长 |  |  | H28家长是否经常带宝宝进行户外活动 |  |
|  | H09宝宝哭闹时，家长是否及时查找原因，并适当处理 |  | 喂养 | H29家长是否在宝宝6月龄时及时添加辅食 |  |
|  | H10家长是否了解宝宝生病的表现 |  |  | H30家长是否知道辅食添加的顺序 |  |
| 亲子关系 | H11访谈期间，家长自然地赞扬宝宝至少2次 |  |  | H31家长给宝宝添加的辅食是否营养全面 |  |
|  | H12宝宝需要临时看护时，是否由熟悉的人来看护 |  |  | H32家长是否采用积极的喂养方式 |  |
|  | H13家长每天与宝宝相处时间不少于2小时 |  | 安全 | H33家长是否注意居住环境的环保 |  |
|  | H14全家经常在一起吃饭并有良好的气氛 |  |  | H34室内是否有安全防护措施 |  |
|  | H15父亲每天也照顾宝宝 |  |  | H35药物、清洁剂、化妆品、锐器、电插座等是否在宝宝够不到的地方 |  |
| 学习材料 | H16宝宝是否拥有能帮助学习颜色、形状、大小的玩具 |  |  | H36家长是否知道紧急救护儿童的知识 |  |
|  | H17宝宝是否拥有锻炼大动作及精细动作的玩具 |  |  | | |
|  | H24家中是否有宝宝自己的图画书 |  |  |  |  |
|  | H19室内是否有符合儿童特点的装饰、图画和用具 |  |  |  |  |
|  | H20宝宝有专门存放玩具或物品的地方 |  |  |  |  |

# 表24I：0~3岁儿童心智发育进程记录

| **序号** | **心智发育项目** | **实际表现月龄** |
| --- | --- | --- |
| I01 | 伏卧抬头45度，能注意父母面部 |  |
| I02 | 伏卧抬头90度 |  |
| I03 | 笑出声、尖叫声、应答性发声 |  |
| I04 | 抱坐时头稳定，能翻身，能手握手、握住摇荡鼓 |  |
| I05 | 伸手抓物 |  |
| I06 | 独坐 |  |
| I07 | 会爬 |  |
| I08 | 听得懂自己的名字，叫名字有反应 |  |
| I09 | 扶东西站 |  |
| I10 | 咿呀学语，躲猫猫，会拍手、摇手表示再见等 |  |
| I11 | 用拇食指捏小物 |  |
| I12 | 有意识叫爸爸、妈妈 |  |
| I13 | 独立行走 |  |
| I14 | 用杯喝水 |  |
| I15 | 走的稳，能叠两块积木 |  |
| I16 | 能说三个字短语 |  |
| I17 | 能走梯 |  |
| I24 | 理解指出身体部分 |  |
| I19 | 会用语言表示大小便 |  |
| I20 | 两脚并跳 |  |
| I21 | 说出自己姓名 |  |
| I22 | 能双脚交替上楼 |  |
| I23 | 会扣纽扣，会折纸，能一页页翻书 |  |

# 表24J：孤独症儿童行为量表（ABC量表）

填表说明：请仔细逐条阅读以下各项目，若您的孩子有该项表现请在得分内填具体分值，若无此项者则填0。量表维度说明：（1）感觉（S），（2）交往（R），（3）.躯体运动（B），（4）语言（L），（5）生活自理（S），评分说明：按每条目在量表中的负荷大小分别评“1”、“2”、“3”、“4”分，如第X项条目分值是“3”，只要儿童有该项表现，无论症状表现轻重都评“3”分，无该项表现者得0分，余类推，各分值相加为量表总得分，总分57分为筛查分，67分为诊断分。

| 条 目 | 维度及分值 | | | | | | | | | 选项 | |  | 维度及分值 | | | | | 得分 |
| --- | --- | --- | --- | --- | --- | --- | --- | --- | --- | --- | --- | --- | --- | --- | --- | --- | --- | --- |
|  | S | R | | B | | L | | S | |  |  |  | S | R | B | L | S |  |
| 1.喜欢长时间自身旋转 |  |  | | 4 | |  | |  | |  | | 21.有时对很大的声音不产生吃惊反应（可能让人想到他是聋子） | 3 |  |  |  |  |  |
| 2.学会做一件简单的事，但很快就忘记 |  |  | |  | |  | | 2 | |  | | 22.经常拍打手 |  |  | 4 |  |  |  |
| 3.经常没有接触环境或进行交往的要求 |  | 4 | |  | |  | |  | |  | | 23.大发脾气或经常发点脾气 |  |  |  |  | 3 |  |
| 4. 往往不能接受简单的指令（如坐下、过来等） |  |  | |  | | 1 | |  | |  | | 24.主动回避与别人的眼光接触 |  | 4 |  |  |  |  |
| 5. 不会玩玩具（如没完没了地转动、乱扔、揉等） |  |  | | 2 | |  | |  | |  | | 25.拒绝别人的接触或拥抱 |  | 4 |  |  |  |  |
| 6.视觉辨别能力差（如对一种物体的特征、大小、颜色、位置等辨别能力差） | 2 |  | |  | |  | |  | |  | | 26.有时对很痛苦的刺激如摔伤、割破或注射不引起反应 | 3 |  |  |  |  |  |
| 7 无交往性微笑（即不会与人点头、招呼、微笑） |  | 2 | |  | |  | |  | |  | | 27.身体表现很僵硬、很难抱住 |  | 3 |  |  |  |  |
| 8 代词运用颠倒或混乱（你、我分不清） |  |  | |  | | 3 | |  | |  | | 28.当抱看他时，感到他的肌肉松驰（即使他不紧贴抱他的人） |  | 2 |  |  |  |  |
| 9总是长时间拿着某种东西 |  |  | | 3 | |  | |  | |  | | 29.以姿势、手势表示所渴望得到的东西（而不倾向于语言表示） |  |  |  | 2 |  |  |
| 10.似乎不在听人说话，以至让人怀疑他有听力问题 | 3 |  | |  | |  | |  | |  | | 30.常用脚尖走路 |  |  | 2 |  |  |  |
| 11.说话不合音调、无节奏 |  |  | |  | | 4 | |  | |  | | 31.用咬人、撞人、踢人等行为伤害他人 |  |  |  |  | 2 |  |
| 12.长时间摇摆身体 |  |  | | 4 | |  | |  | |  | | 32.不断地重复短句 |  |  |  | 3 |  |  |
| 13.要去拿什么东西，但又不是身体所能达到的地方（即对自身与物体的距离估计不足) | 2 |  | |  | |  | |  | |  | | 33.游戏时不模仿其他儿童 |  | 3 |  |  |  |  |
| 14.对环境和日常生活规律的改变产生强烈反应 |  |  | |  | | 3 | |  | |  | | 34.当强光直接照射眼睛时常常不眨眼 | 1 |  |  |  |  |  |
| 15.当与其他人在一起时，呼唤他的名字，他没有反应 |  |  | | 2 | |  | |  | |  | | 35.以撞头、咬手等行为自伤 |  |  | 2 |  |  |  |
| 16.经常做出前冲、旋转、脚尖行走、手指轻掐轻弹等动作 |  | 4 | |  | |  | |  | |  | | 36.想要什么东西不能等待（一想要什么，就马上要得到） |  |  |  |  | 2 |  |
| 17.对其他人的面部表情没有反应 | 3 |  | |  | |  | |  | |  | | 37.不能指出5个以上物体的名称 |  |  |  | 1 |  |  |
| 18.说话时很少用“是”或“我”等词 |  |  | | 2 | |  | |  | |  | | 38.不能发展任何友谊（不会和小朋友来往交朋友） |  | 4 |  |  |  |  |
| 19.有某一方面的特殊能力，似乎与智力低下不相符合 |  |  | |  | |  | | 4 | |  | | 39.有许多声音的时候，常常捂着耳朵 | 4 |  |  |  |  |  |
| 20.不能执行简单的含有介词语句的指令（如把球放在盒子上或放在盒子里） |  |  | | 1 | |  | |  | |  | | 40.经常旋转碰撞物体 |  |  | 4 |  |  |  |
| 条 目 | 维度及分值 | | | | | | | | | 得分 | | 条 目 | 维度及分值 | | | | | 得分 |
|  | S | | R | | B | | L | | S | |  |  | S | R | B | L | S |  |
| 41.在训练大小便方面有困难（不会控制大小便） |  | |  | |  | |  | | 1 | |  | 50.特别喜欢摆弄、着迷于单调的东西或游戏、活动等（如来回地走或跑，没完没了地蹦、跳、拍、敲） |  |  |  |  | 4 |  |
| 42.一天只能提出5个以内的要求 |  | |  | |  | | 2 | |  | |  | 51.对周围东西喜欢嗅、摸或尝 |  |  | 3 |  |  |  |
| 43.经常受到惊吓或非常焦虑不安 |  | | 3 | |  | |  | |  | |  | 52.对生人常无视觉反应（对来人不看） | 3 |  |  |  |  |  |
| 44.在正常光线下斜眼、闭眼、皱眉 | 3 | |  | |  | |  | |  | |  | 53.纠缠在一些复杂的仪式行为上，就像缠在魔圈里（如走路要走一定的路线，饭前或做什么事前一定要把什么东西摆在什么位置，或做什么动作，否则就不睡不吃） |  |  | 4 |  |  |  |
| 45.不是经常被帮助的话，不会自己给自己穿衣 |  | |  | |  | |  | | 1 | |  | 54.经常毁坏东西（如玩具、家里的一切用具很快就给弄坏了） |  |  |  |  |  |  |
| 46.一遍遍重复一些声音或词 |  | |  | |  | | 3 | |  | |  | 55.在2岁以前就发现孩子发育延迟 |  |  | 2 |  | 1 |  |
| 47.瞪着眼看人，好像要看穿似的 |  | | 4 | |  | |  | |  | |  | 56.在日常生活中至少用15个但不超过30个短句进行交往（不到15句也打“∨”） |  |  |  | 3 |  |  |
| 48.重复别人的问话或回答 |  | |  | |  | | 4 | |  | |  | 57.长时间疑视一个地方（呆呆地看一处） | 4 |  |  |  |  |  |
| 49.经常不能意识所处的环境，并且可能对危险的环境不在意 |  | |  | |  | |  | | 2 | |  |  | | | | | | |
