# Supplementary material for: Effect of feeding patterns on growth and nutritional status of children aged 0-24 months: A Chinese cohort study
Source: PLoS One. 2019 Nov 19;14(11):e0224968. doi: 10.1371/journal.pone.0224968 (PMC6863544; doi:10.1371/journal.pone.0224968)
Supplement: S2 Text — (ZIP) [file pone.0224968.s002.zip › 6 months old.doc]

**2015 Kaifu District Community Maternal and child health information collection（6 month-old）**

**number： □□□□□□**

Kaifu District	Community Health Service center

Residential Address：	District(country)	Street(road)	Community

Mother’s name：	Telephone number：

Father’s name：	Telephone number：

Name of child：	Gender of child：

Date of birth of the child：

Information collection time：	year	month	day	Information collector：

# Tab 6B：Follow-up record form for children aged 6 months

| **6B01** | Check date： year month day |
| --- | --- |
| **6B02** | Real term age： month-old day |
| **6B03** | Feeding style：（1）Exclusive breastfeeding（2）Mixed feeding （3）Formula feeding **(skip to 6B07)** |
| **6B04** | Number of breast milk： （ times/day） |
| **6B05** | Is the baby now out of breast milk？（1）No **(skip to 6B07)** （2）Yes，the month age of breast milk disconnection is month-old day |
| **6B06** | What the reason why you weaned your child? **（ can be more selected）**⑴Job requirements ⑵Fall ill ⑶No breast milk ⑷Feel the hassle of breastfeeding ⑸Worry about figure or image ⑹Think formula powder id more nutritious ⑺The child is ill ⑻Children refuse to suck ⑼Else，please specify |
| **6B07** | How long are you going to breastfeed？ month |
| **6B08** | Do you child currently use a bottle with nipple to drink water, milk or juice? ⑴No ⑵Yes |
| **6B09** | Do you add formula milk to your child?（1）No**（skip to 6B11）**（2）Yes，the first time you add formula milk to your child is： month-old day |
| **6B10** | Formula milk feeding situation： （ times/day）， ml at every time |
| **6B11** | Your child’s sleep：⑴Normal ⑵Abnormal，such as difficult to fall asleep, frequent night wake, sleep rhythm disorder） |
| **6B12** | Sleep time: （ hours/day） |
| **6B13** | How your child sleeps？（1）Fall asleep by himself（2）Rely on outside help, such as holding sleep, shaking sleep, etc。 |
| **6B14** | Is your child in a stable mood before bedtime?（1）No（2）Basic stability（3）Instability |
| **6B15** | How many times your child eats at night:_______ |
| **6B16** | Does the child sleep with his or her parents?  (1)No (2)Sometimes (3)Always |
| **6B17** | Outdoor activities： （ hours/day） |
| **6B18** | Take vitamin D：（ **IU/**day） |
| **6B19** | weight： （ **Kg**） |
| **6B20** | length： （ **cm**） |
| **6B21** | Head circumference： （ **cm**） |
| **6B22** | Number of teeth： （ ） |
| **6B23** | Former fontanel： （ **cm**× **cm**） |
| **6B24** | Physical examination：⑴Normal ⑵Abnormal，please specify ______ |
