# Supplementary material for: Effect of feeding patterns on growth and nutritional status of children aged 0-24 months: A Chinese cohort study
Source: PLoS One. 2019 Nov 19;14(11):e0224968. doi: 10.1371/journal.pone.0224968 (PMC6863544; doi:10.1371/journal.pone.0224968)
Supplement: S2 Text — (ZIP) [file pone.0224968.s002.zip › 6╘┬┴Σ.doc]

2015年开福区社区母婴健康信息采集(6月龄)

**编号： □□□□□□**

开福区	社区卫生服务中心

居住地址：	区（县）	街道（路）	社区

母亲姓名：	联系电话：

父亲姓名：	联系电话：

子女姓名：	子女性别：

子女出生日期：

信息收集时间：	年	月	日	信息收集者：

目录

表6A：产后情况调查表	3

表6B：6月龄儿童随访记录表	3

表6C：儿童患病情况	4

表6D：婴儿添加辅食情况表	6

表6E： 儿童环境危险因素调查表	7

# 表6A：产后情况调查表

| **6A01** | 您产假时长 天? |
| --- | --- |
| **6A02** | 您现在的就业状况？（1）在岗（2）离职 （3）待业（4）其他（请注明） |
| **6A03** | 您每天的睡眠时长？ h，与孕前比较？（1）减少（2）增多（3）无差异 |
| **6A04** | 您产后6月的体重： Kg |
| **6A05** | 您现在是否锻炼: ⑴否**(跳至6A07)** ⑵是，平均每天锻炼 分钟 |
| **6A06** | 您最常用的锻炼方式是哪一种: ⑴步行锻炼 ⑵中等强度体育锻炼（如慢跑、慢速游泳、太极拳、乒乓球、扇子舞、扭秧歌等）  ⑶大强度体育锻炼（如中速跑步、中速游泳、足球、篮球、羽毛球等） |
| **6A07** | 您现在饮食是否仍注意自身营养的需要？⑴否 ⑵是 |
| **6A08** | 儿童父亲现在吸烟吗： ⑴不吸烟 ⑵每天吸烟 ⑶不是每天吸烟 |
| **6A09** | 儿童父亲现在是否饮酒？ ⑴否 ⑵是 |
| **6A10** | 您现在是否吸烟: ⑴不吸烟 ⑵每天吸烟 ⑶不是每天吸烟 |
| **6A11** | 您现在是否饮酒？ ⑴否 ⑵是 |
| **6A12** | 目前，您平均每周被动吸烟的天数（每天超过15分钟）： ⑴0天 ⑵1-2天 ⑶3-5天 ⑷6-7天 （99）不详 |
| **6A13** | 目前，孩子平均每周被动吸烟的天数（每天超过15分钟）： ⑴0天 ⑵1-2天 ⑶3-5天 ⑷6-7天 （99）不详 |
| **6A14** | 婴儿目前的主要看护人？（1）父母亲（2）（外）祖父母（3）保姆（钟点工）（4）亲属或朋友（5）其他 （99）不详 |

# 表6B：6月龄儿童随访记录表

| **6B01** | 检查日期： （ 年 月 日） |
| --- | --- |
| **6B02** | 实足月龄： （ 月龄 天） |
| **6B03** | 喂养方式：（1）纯母乳喂养 （2）人工喂养**（跳至6B07）** （3）混合喂养 |
| **6B04** | 母乳次数： （ 次/日） |
| **6B05** | 婴儿是否已断母乳？（1）否**（跳至6B07）** （2）是，断母乳的月龄是 月 天 |
| **6B06** | 您给孩子断奶的原因是什么？**（可多选，任一选项均跳至6B08）** ⑴工作需求 ⑵生病 ⑶无母乳 ⑷觉得母乳喂养麻烦 ⑸担心影响身材或形象 ⑹认为配方粉更有营养 ⑺孩子生病 ⑻孩子拒绝吸吮 ⑼其他，（请注明）： |
| **6B07** | 打算母乳喂养多久？ 月 |
| **6B08** | 目前孩子是否使用带奶嘴的奶瓶喝水、奶类或果汁等？ ⑴否 ⑵是 |
| **6B09** | 目前您是否给孩子添加配方奶或鲜奶？（1）未添加**（跳至6B11）** （2）已添加，首次添加时间为： 月龄 天 |
| **6B10** | 配方奶情况： （ 次/天），每次 **ml** |
| **6B11** | 孩子的睡眠：（⑴正常 ⑵异常，如入睡困难，频繁夜醒，睡眠节律紊乱） |
| **6B12** | 睡眠时间: 小时/日 |
| **6B13** | 孩子入睡方式？（1）自行入睡（2）依赖外界帮助，如抱睡、摇晃入睡、吃奶入睡等。 |
| **6B14** | 孩子睡前情绪是否稳定？（1）否（2）基本稳定（3）不稳定 |
| **6B15** | 孩子夜间进食次数： 次 |
| **6B16** | 孩子是否与父母同睡？（1）否（2）有时同睡 （3）一直同睡 |
| **6B17** | 户外活动： （ 小时/日） |
| **6B18** | 服用维生素**D**：（ **IU**/日） |
| **6B19** | 体重： （ **Kg**） |
| **6B20** | 身长： （ **cm**） |
| **6B21** | 头围： （ **cm**） |
| **6B22** | 孩子的出牙数： （ 颗） |
| **6B23** | 前囟： （ **cm**× **cm**） |
| **6B24** | 体格检查：⑴正常 ⑵异常,请注明 ­­­________ |

# 表6C：儿童患病情况

| **6C01** | | | 您的孩子自出生到1月龄对以下哪些物质有过敏情况**（可多选）**： ⑴无 （2）牛奶 （3）奶酪 （4）全蛋 （5）尘螨 （6）家尘  （7）牛肉 （8）鱼虾蟹贝蛤 （9）狗毛鸡毛 （10）蟑螂 （11）橙（12）各类真菌 （13）黄豆 （14）各类花粉 （15）花生  （16）青胡椒 （17）小麦 （18）鸡肉 （19）蘑菇 （20）其他（请注明） （99）不详 | | | | | | | | | | | | | | | |
| --- | --- | --- | --- | --- | --- | --- | --- | --- | --- | --- | --- | --- | --- | --- | --- | --- | --- | --- |
| **6C02** | | | 您的孩子3月龄到6月龄有无药物过敏情况**（可多选）**：（1）无**（跳至6C04）**（2）有（请注明） | | | | | | | | | | | | | | | |
| **6C03** | | | 您的孩子发生药物过敏反应的给药途径：（1）静脉滴注 （2）皮下注射 （3）静脉注射（4）肌肉注射（5）口服给药（99）不详 | | | | | | | | | | | | | |  | |
| **6C04** | | | 您的孩子3月龄到6月龄患过以下哪些过敏性疾病：  （1）无（2）过敏性皮炎 （3）过敏性鼻炎（4）过敏性哮喘（5过敏性紫癜（6）过敏性休克 （7）其他 | | | | | | | | | | | | | |  | |
| **6C05** | | | 您的孩子1月龄到3月龄患过以下哪些传染病：  ⑴无 ⑵水痘 （3）腮腺炎 （4）风疹 （5）麻疹 （6）手足口病 （7）其他传染病（请注明） （99）不详 | | | | | | | | | | | | | |  | |
| **6C06** | | | 您的孩子3月龄到6月龄是否患过下列疾病**（可多选）：**   1 无 ⑵感冒 ⑶腹泻 ⑷支气管炎 ⑸肺炎 ⑹哮喘 ⑺佝偻病 ⑻贫血 ⑼体弱儿 ⑽其他(请注明) | | | | | | | | | | | | | |  | |
| **6C07** | | | 若患过感冒，感冒次数为： 次 | | | | | | | | | | | | | |  | |
| **6C08** | | | 若患过腹泻，腹泻次数为： 次 | | | | | | | | | | | | | |  | |
| **6C09** | | | 您的孩子3月龄到6月龄是否有因病就医行为： ⑴否**（跳至6C14）** ⑵是 | | | | | | | | | | | | | |  | |
|  | | | 就诊次数 | | 01.  就诊月龄 | 02.  就诊单位名称 | | 03.  就诊单位类型：⑴药店⑵诊所⑶医院（4）其他 | | 04.  最终诊断 | | 05.  主要治疗药物 | 06.  其他处理 | | 07.  疾病转归：⑴痊愈 ⑵好转⑶无变化 ⑷恶化 ⑸其他 | | | |
| **6C10** | | | 第1次 | |  |  | |  | |  | |  |  | |  | | | |
| **6C11** | | | 第2次 | |  |  | |  | |  | |  |  | |  | | | |
| **6C12** | | | 第3次 | |  |  | |  | |  | |  |  | |  | | | |
| **6C13** | | | 第4次 | |  |  | |  | |  | |  |  | |  | | | |
| **6C14** | 您的孩子是否发生过意外伤害？ ⑴没有**（跳至表6D）** ⑵发生过 | | | | | | | | | | | | | | |  | |  |
| **6C15** | 如果发生过，是哪种或哪些呢？**(可多选，并分别将代码填入下表中)**  （1）外伤（车祸、坠落、跌伤、滑倒、磕伤钝器伤等）（2）烧烫伤（3）利器伤（4）化学品损伤或中毒（5）气管异物伤或窒息（6）触电（7）动物咬伤（8）溺水（9）其他（请注明） （99）不详 | | | | | | | | | | | | | | |  | |  |
| 0-12月龄内的孩子意外伤害基本情况? | | | | | | | | | | | | | | | | | |  |
|  | | 伤害的次数 | | 01  发生伤害的种类？ | | | 02  伤害发生的月龄？ | | 03  处理伤害的方式？  （1）家庭处理（2）送医处理（3）未加处理（99）不详 | | 04  伤害发生时的看护人？  （1）无（2）父母亲（3）爷爷奶奶（4）保姆或护工（5）亲属或朋友（6）其他（99）不详 | | | 05  现在孩子恢复情况？  （1）恢复情况尚不乐观（2）有明显好转，但尚未完全恢复（3）已经完全恢复（99）不详 | | | |  |
| **6C16** | | 第1次伤害 | |  | | |  | |  | |  | | |  | | | |  |
| **6C17** | | 第2次伤害 | |  | | |  | |  | |  | | |  | | | |  |
| **6C18** | | 第3次伤害 | |  | | |  | |  | |  | | |  | | | |  |
| **6C19** | | 第4次伤害 | |  | | |  | |  | |  | | |  | | | |  |
| **6C20** | | 第5次伤害 | |  | | |  | |  | |  | | |  | | | |  |
| **6C21** | | 第6次伤害 | |  | | |  | |  | |  | | |  | | | |  |
| **6C22** | | 第7次伤害 | |  | | |  | |  | |  | | |  | | | |  |
| **6C23** | | 第8次伤害 | |  | | |  | |  | |  | | |  | | | |  |
| **6C24** | | 第9次伤害 | |  | | |  | |  | |  | | |  | | | |  |
| **6C25** | | 第10次伤害 | |  | | |  | |  | |  | | |  | | | |  |

# 表6D：婴儿添加辅食情况表

| **6D01** | 目前孩子是否已经添加辅食：⑴否 ⑵是，添加辅食的月龄 月 | | | |
| --- | --- | --- | --- | --- |
| **6D02** | 昨天（包括白天和夜里），孩子吃过辅食的种类： 种。 | | | |
| **6D03** | 昨天（包括白天和夜里），孩子吃过 餐次辅食 (填次数，辅食指正餐和零食） | | | |
|  | **食物品种** | **食用频率** | **6D04**  **食用频率** | **6D05**  **开始添加月龄** |
| **01** | 谷薯类 | (1)未添加 ⑵每天吃1次 ⑶每天吃≥2次 |  |  |
| **02** | 奶类及制品 | (1)未添加 ⑵每月吃1~2次 ⑶每周吃1~2次 ⑷每周吃3~4次 ⑸每周吃≥5次 |  |  |
| **03** | 蛋类 | (1)未添加 ⑵每月吃1~2次 ⑶每周吃1~2次 ⑷每周吃3~4次 ⑸每周吃≥5次 |  |  |
| **04** | 禽畜肉类 | (1)未添加 ⑵每月吃1~2次 ⑶每周吃1~2次 ⑷每周吃3~4次 ⑸每周吃≥5次 |  |  |
| **05** | 鱼类 | (1)未添加 ⑵每月吃1~2次 ⑶每周吃1~2次 ⑷每周吃3~4次 ⑸每周吃≥5次 |  |  |
| **06** | 豆类及制品 | (1)未添加 ⑵每月吃1~2次 ⑶每周吃1~2次 ⑷每周吃3~4次 ⑸每周吃≥5次 |  |  |
| **07** | 新鲜水果 | (1)未添加 ⑵每月吃1~2次 ⑶每周吃1~2次 ⑷每周吃3~4次 ⑸每周吃≥5次 |  |  |
| **08** | 新鲜蔬菜 | (1)未添加 ⑵每月吃1~2次 ⑶每周吃1~2次 ⑷每周吃3~4次 ⑸每周吃≥5次 |  |  |
| **09** | 动物内脏 | (1)未添加 ⑵每月吃1~2次 ⑶每周吃1~2次 ⑷每周吃3~4次 ⑸每周吃≥5次 |  |  |
| **10** | 坚果 | (1)未添加 ⑵每月吃1~2次 ⑶每周吃1~2次 ⑷每周吃3~4次 ⑸每周吃≥5次 |  |  |
| **11** | 食用油 | (1)未添加 ⑵每月吃1~2次 ⑶每周吃1~2次 ⑷每周吃3~4次 ⑸每周吃≥5次 |  |  |

# 表6E： 儿童环境危险因素调查表

| **所居住社区外环境调查** | | |
| --- | --- | --- |
| 6E01 | 自怀孕起，社区外环境中是否存在空气污染？ （1）否**（跳至6E04）** （2）是 （99）不详 |  |
| 6E02 | 如果存在空气污染请选择是何种污染**（可多选）** （1）汽车尾气污染 （2）工业废气污染 （3）生活污染源污染  （4）其他常见污染，(请注明) （99）不详 |  |
| 6E03 | 污染程度如何？（1）非常严重 （2）严重 （3）一般 （4）轻微 （99）不详 |  |
| 6E04 | 自怀孕起，社区周围环境存在哪些噪音？**（可多选）**  （1）无 （2）生产噪音：工矿企业，建筑施工等噪音 （3）生活（社会）噪音:人类生活活动产生的噪音 （4）交通噪音：机动车辆，火车，飞机等产生的噪音 （5）其他噪音，（请注明） （99）不详 |  |
| 6E05 | 您居住社区周围是否有基站？ （1）否（2）是 （99）不详 |  |
| **家庭室内环境调查** | | |
| 6E06 | 是否存在以下室内污染现象？**（可多选）**  （1）无**（跳至6E07）** （2）每天清晨起床时，感到[憋闷](http://zhidao.baidu.com/search?word=憋闷&fr=qb_search_exp&ie=utf8)、恶心、甚至头晕目眩。  （3）家里小孩常咳嗽、打喷嚏、免疫力下降，新装修的房子孩子不愿回家。  （4）家人共有一种疾病，而且离开这个环境后，症状就有明显变化和好转。  （5）家人常有皮肤过敏等毛病，而且是群发性的。 （6）其他 |  |
| 6E07 | 是否存在以下家庭环境污染的因素？**（可多选）**  （1）无 （2）自怀孕前半年以来，进行过房屋装修或添置新家具 （3）采暖、烹饪产生油烟 （4）（基本）不进行房屋通风换气 （5）家中宠物（基本）不进行定期健康检查 （6）其他，（请注明） |  |
| 6E08 | 您的孩子在室内受噪音困扰的程度？ （1）否 **（2）**轻微（3）一般（4）严重（5）非常严重 （99）不详 |  |
| 6E09 | 您的孩子生活环境中是否存在辐射？（1）否 （2）是 （99）不详 |  |
| 6E10 | 有孩子以来，孩子生活范围内，是否有人患传染病？（1）否（2）是 （99）不详 |  |
| 6E11 | 是何种传染病？**（可多选）** （1）流感 （2）流行性腮腺炎 （3）百日咳 （4）水痘 （5）麻疹 （6）乙型脑炎  （7）菌痢 （8）流脑 （9）其他，请注明 （99）不详 |  |
